# Untargeted analysis of first trimester serum to reveal biomarkers of pregnancy complications: a case–control discovery phase study

**DOI:** 10.1038/s41598-021-82804-1

**Published:** 2021-02-10

**Authors:** E. W. Harville, Y.-Y. Li, K. Pan, S. McRitchie, W. Pathmasiri, S. Sumner

**Affiliations:** 1 Department of Epidemiology, Tulane University School of Public Health and Tropical Medicine, Epidemiology #8318, 1440 Canal St. Ste. 2001, New Orleans, LA 70112 USA; 2 Department of Nutrition, Nutrition Research Institute, University of North Carolina at Chapel Hill School of Public Health, CB#74612, Chapel Hill, NC 27599-7461 USA

**Keywords:** Biomarkers, Reproductive disorders

## Abstract

Understanding of causal biology and predictive biomarkers are lacking for hypertensive disorders of pregnancy (HDP) and preterm birth (PTB). First-trimester serum specimens from 51 cases of HDP, including 18 cases of pre-eclampsia (PE) and 33 cases of gestational hypertension (GH); 53 cases of PTB; and 109 controls were obtained from the Global Alliance to Prevent Prematurity and Stillbirth repository. Metabotyping was conducted using liquid chromatography high resolution mass spectroscopy and nuclear magnetic resonance spectroscopy. Multivariable logistic regression was used to identify signals that differed between groups after controlling for confounders. Signals important to predicting HDP and PTB were matched to an in-house physical standards library and public databases. Pathway analysis was conducted using GeneGo MetaCore. Over 400 signals for endogenous and exogenous metabolites that differentiated cases and controls were identified or annotated, and models that included these signals produced substantial improvements in predictive power beyond models that only included known risk factors. Perturbations of the aminoacyl-tRNA biosynthesis, l-threonine, and renal secretion of organic electrolytes pathways were associated with both HDP and PTB, while pathways related to cholesterol transport and metabolism were associated with HDP. This untargeted metabolomics analysis identified signals and common pathways associated with pregnancy complications.

## Introduction

Hypertensive disorders of pregnancy (HDP) and preterm birth (PTB) are significant causes of obstetric morbidity^1^. It remains unclear whether the subtypes of HDP, pre-eclampsia (PE) and gestational hypertension (GH), are distinct conditions or on a spectrum, though they share common risk factors^2,3^. The subtypes of PTB, spontaneous or medically indicated^4^, also share risk factors^5^. Knowledge about the causes of both HDP and PTB is limited^6^; HDP has been related to placentation, inflammation and progressive endothelial damage^1^, and PTB to activation of the hypothalamic–pituitary–adrenal axis and exaggerated inflammatory response^7,8^. Although several biomarkers have been linked to HDP and PTB, none are clinically useful in early pregnancy^9,10^.

Untargeted metabolomics captures signals for low molecular weight compounds from exogenous exposures (e.g., environmental chemicals, medication, and food), and endogenous metabolites produced by the host system that map to biochemical pathways. Untargeted metabolomic approaches have been used to investigate PE^11–19^ (few studies have examined GH specifically^14^); however, the key metabolites identified across studies have not been consistent^3,6^ and the pooled sensitivity of all single biomarkers is low^10^. Untargeted metabolomics has also been used to investigate PTB, and, again, the key metabolites have not been consistent across studies^20^. Differences in study design, population, biospecimens, and included/overlooked confounders likely contribute to this variability^20^.

This study therefore performed an untargeted metabolomic analysis of first-trimester serum to further reveal biomarkers for both HDP and PTB. We extend previous analyses by incorporating two types of spectroscopic analysis, providing the evidence for metabolite identification and annotation, and using modelling approaches to determine the predictive value that metabolites add over known risk factors.

## Methods

### Study population

Serum specimens were obtained from the Global Alliance to Prevent Prematurity and Stillbirth (GAPPS) repository, taken between 2011 and 2016 (demographic and lifestyle information on the registry, Table S1). Two overall case groups were identified: HDP and PTB. 51 cases of HDP (including 18 cases of PE and 33 cases of GH) and 53 cases of PTB (42 spontaneous) were frequency-matched for gravidity to 109 controls. Pregnant women ≥ 14 years of age can be enrolled in GAPPS Repository; exclusion criteria include: received narcotics in the previous 12 h, in active labor, or multiple gestations. Participants were enrolled during pregnancy, usually at a prenatal care appointment, from the University of Washington Medical Center, Seattle; Swedish Medical Center, Seattle; and Yakima Valley Memorial Hospital, Yakima, WA. All participants were followed throughout pregnancy, delivery and up to 10 weeks postpartum.

### Clinical definitions

Participant medical records were abstracted by GAPPS study personnel, and recorded HDP (PE and GH) and PTB (births < 37 weeks gestation) were used to define case groups. Two overall case groups were examined, then their subgroups: all HDP, then PE and GH separately; all PTB, then limited to spontaneous PTB (sPTB; dataset did not allow for distinguishing other subtypes of PTB). HDP and PTB cases were selected separately and did not overlap, but according to the medical records abstraction, 7 PE cases gave birth preterm and 1 case of PTB had GH. A sensitivity analysis including these in the other respective case groups was conducted and did not change the results, so the original case groups are used in this report.

### Covariate collection

Questionnaires were used to collect information on demographics, health history, diet, and home and work environment. Three questionnaires were collected on participants who enrolled before May 20, 2014; afterwards, 5 questionnaires were collected. When necessary, covariate definitions were harmonized across questionnaires.

### Metabolomics analysis

Details of the sample preparation, data acquisition, data preprocessing, metabolite identification and annotation, and statistical analysis are provided in the Supplementary material.

Serum samples were collected from participants during the first trimester of pregnancy (gestational age range: 6^+1^–13^+6^ weeks), and prepared according to published methods^21,22^. Untargeted metabolomics data was acquired using a Vanquish UHPLC system coupled with a Q Exactive HF-X Hybrid Quadrupole-Orbitrap Mass Spectrometer (UPLC-HR-MS; Thermo Fisher Scientific) and using a Bruker Avance III 700 MHz NMR spectrometer The UPLC-HR-MS data was processed using Progenesis QI (Waters Corporation), and the NMR data was processed using Chenomx NMR Suite 8.4. UPLC-HR-MS signals were identified or annotated through matching with an in-house physical standards library or public databases.

### Statistical analysis

The Caret R package (version 6.0-84) and RStudio 3.6.1 were used for the UPLC-HR-MS model selection procedure based on cross validation with SAS software 9.4 for the remaining data analysis. All demographic, behavioral, medical, and lifestyle factors available or harmonizable across questionnaires and potentially associated with exposure and the outcomes were examined as possible confounders. Covariates that were distributed differently in cases and controls with *p* value < 0.2, with the exception of previous history of complications (because causes of previous events might also cause events in the current pregnancy^23^), were included in the initial stepwise models. Due to the sample selection criteria, “gravidity” was included in each of the stepwise models regardless of significance level.

Each case group was modeled separately using univariate and multivariable logistic regression models. All signals meeting the selection criterion, regardless of being identified/annotated, were considered in the analysis. The first set of multivariable regression models utilized all 3,122 signals, and due to the high dimensionality of the data we utilized a multi-step approach based on a fivefold cross validation (supplementary materials)^24^. In addition, the 12 exogenous metabolites that were identified or annotated based on the untargeted LC–MS in-house physical standards library and differed by group were modeled using stepwise multivariable regression with *p* < 0.05 for retention; the covariates identified above were included regardless of the *p* value. The broad spectrum NMR data (195 bins) was modeled using stepwise multivariable regression. Stepwise regression was used for the exogenous signals and the NMR data due to the lower dimensionality of the data.

The area under the receiver operating characteristic curve (AUC) was used to evaluate the performance of the prediction models. The strength and precision of the associations with individual metabolites were compared based on the odds ratios and widths of the confidence interval respectively.

### Pathway enrichment analysis

GeneGo MetaCore (Clarivate Analytics, PA) was used to assess the enrichment of perturbed metabolic pathways. For this analysis, metabolites were included that had an ontology level (OL) of OL1 (RT, Mass, and MS/MS), OL2a (RT and Mass), or were determined by NMR (Tables S2–S11). Metacore uses the hypergeometric test, which represents the enrichment of certain metabolites in a pathway, together with the false discovery rate (FDR). A *p* value < 0.01 is considered indicative of significant enrichment in pathways.

This secondary analysis of de-identified data and samples was ruled not human subjects research by the Tulane Institutional Review Board. All participants provided informed consent to recruitment into the GAPPS repository^25^.

## Results

The largest proportion of included samples were from Yakima Valley Memorial Hospital, with 14.1% of participants from the University of Washington Medical Center and 14.1% from Swedish Medical Center; there was no statistically significant association between pregnancy complications and center where participants were enrolled. The mean participant age was between 29 and 31 for all case groups and for the controls (Table 1). A large majority had been pregnant before (78% of controls, 72–79% depending on case group). The majority of the participants were white (72% of controls, 69% of HDP cases, 53% of PTB cases). Early-pregnancy BMI of those with hypertensive disorders (mean for HDP cases, 34.7, SD 9.6) was higher than controls (mean 29.3, SD 7.8). Cases of overall PTB (29% ever smokers) and GH (39% ever smokers) were more likely to have smoked than controls (17%). Cases were more likely to have used street drugs prior to pregnancy (overall HDP 17%, overall PTB 15%) than controls (6%). Other variables that differed from controls for at least one case group are listed in Table 1. Besides gravidity, for the stepwise modeling, BMI and illegal drug use were included in models of HDP, GH, and PE; obesity and illegal drug use were selected for the PTB model; and no covariates remained in the sPTB model.

**Table 1:** Demographic, medical, and lifestyle characteristics of cases and controls.

	Uncomplicated (n = 109)	HDP (n = 51)	GH (N = 33)	PE (N = 18)	PTB (n = 53)	sPTB (n = 42)
	Mean (SD)/N (%)	Mean (SD)/N (%)	Mean (SD)/N (%)	Mean (SD)/N (%)	Mean (SD)/N (%)	Mean (SD)/N (%)
Age	29.87 (5.08)	29.33 (5.73)	29.36 (4.76)	29.59 (7.43)	30.9 (6.89)	30.32 (7.04)
BMI at first prenatal visit^f,i^	29.29 (7.83)	34.69 (9.56)	35.38 (10.23)	33.74 (8.35)	30.14 (8.47)	29.91 (7.84)
Gestational age when blood sample were collected	10.51 (1.83)	10.74 (1.72)	10.53 (1.65)	11.02 (1.84)	10.63 (2.07)	10.78 (2.06)
**Gravidity**						
1	24 (22.02)	13 (25.49)	8 (24.24)	5 (27.78)	11 (20.75)	9 (21.43)
2	30 (27.52)	15 (29.41)	10 (30.30)	5 (27.78)	15 (28.3)	13 (30.95)
3	30 (27.52)	15 (29.41)	11 (33.33)	4 (22.22)	12 (22.64)	8 (18.05)
> 3	25 (22.94)	8 (15.69)	4 (12.12)	4 (22.22)	15 (28.3)	12 (28.57)
**Race**^g^						
White	78 (71.56)	36 (69.23)	25 (75.76)	11 (61.11)	28 (52.83)	26 (61.90)
Non-White	31 (28.44)	16 (30.77)	8 (24.24)	7 (38.89)	25 (47.17)	16 (38.10)
Previous gestational hypertension^d,f,h^	4 (3.67)	8 (15.38)	6 (18.18)	2 (11.11)	1 (1.89)	1 (2.38)
Previous eclampsia^f,i^	3 (2.75)	7 (13.46)	1 (3.03)	6 (33.33)	3 (5.66)	3 (7.14)
Pre-pregnancy obesity (medical records) ^b,e,f^	31 (29.25)	27 (51.92)	19 (57.58)	8 (44.44)	23 (43.4)	18 (42.86)
Gestational age at delivery^b,c,e,f,i^	39.44 (0.93)	38.25 (2.63)	39.16 (1.03)	36.65 (3.79)	34.29 (3.79)	34.28 (3.81)
Previous GDM^a,b,d,e^	2 (1.83)	4 (7.69)	2 (6.06)	2 (11.11)	4(7.55)	4 (9.52)
Smoked more than 100 cigarettes (about 5 packs) in your lifetime^b, f,h^	17 (16.67)	16 (32)	13 (39.39)	3 (18.75)	14 (28.57)	10 (25.64)
**Frequency exposed to second-hand smoke**^a,d^						
Never	29 (29)	10 (21.74)	8 (27.59)	2 (12.50)	11 (23.91)	8 (22.22)
Rarely	47 (47)	17 (36.96)	10 (34.48)	7 (43.75)	20 (43.48)	15 (41.67)
Almost/everyday	24 (24)	19 (41.3)	11 (37.93)	7 (43.75)	15 (32.61)	13 (36.11)
Use of marijuana or street drugs in the year before pregnancy^d,e,f,g,h^	5 (5.59)	9 (17.31)	6 (18.18)	3 (16.67)	8 (15.09)	5 (11.90)
Night shift work during pregnancy^a,h^	10 (16.13)	10 (33.33)	8 (38.1)	2 (22.22)	4 (17.39)	2 (10.53)

Mean (SD): for continuous variables, mean and standard deviation are displayed; N (%): for categorical variables, number and percentage in case/control are displayed.

HDP, any hypertensive disorder of pregnancy; GH, gestational hypertension; PE, pre-eclampsia, PTB, preterm birth; sPTB, spontaneous preterm birth; GDM, gestational diabetes mellitus.

^a^*p* < 0.2 (criterion for further inclusion in models) comparing HDP cases to controls.

^b^*p* < 0.2, PTB cases vs. controls.

^c^*p* < 0.2 GH cases vs. controls.

^d^*p* < 0.2 PE cases vs. controls.

^e^*p* < 0.2 sPTB cases vs. controls.

^f^*p* < 0.05, HDP cases vs. controls.

^g^*p* < 0.05, PTB cases vs. controls.

^h^*p* < 0.05, GH cases vs. controls.

^i^*p* < 0.05, PE cases vs. controls.

When examined one at a time, 337 signals were associated with HDP (*p* < 0.1) with 173 metabolites being identified or annotated (Table S2). When GH and PE were examined individually, 344 signals (with 173 being identified or annotated) were associated with GH (*p* < 0.1, Table S3), while 446 (with 189 being identified or annotated) were associated with PE (*p* < 0.1, Table S4).

Models including signals/metabolites determined by UPLC-HRMS (Table 2) showed significant improvements in the AUC over models constructed using only covariates. Among the signals/metabolites retained in the HDP models, the most precise associations were with an unknown signal with an neutral mass of 746.6045 Da and retention time at 0.59 min (0.59_746.6045n, reduced odds), and a signal annotated as pilocarpine (PDc, increased odds). The strongest effect sizes were for an unidentified signal at 8.66_762.1452 m/z and 12.74_412.2842 m/z, both of which were associated with reduced odds. A signal that annotated as 2,6-Di-*tert*-butyl-4-hydroxymethylphenol (BHT-OH) through matching with public database by exact mass and MS/MS spectra (PDa) was strongly associated with GH. For the PE model, 4 signals were included; among them, an unidentified signal at 6.30_477.7721 m/z was most precise, while cerasinone (PDb) had the strongest effect size and bolasterone the most definite annotation (PDa) (Table 2). The signals/metabolites included in the overall HDP model were not the same as those included for models of each type of HDP, but signals/metabolites included in the final HDP model were associated with either GH or PE, and usually both, when examined individually (Tables S2–S4).

**Table 2:** Metabolites/signals that predicted HDP, GH, PE, PTB, and sPTB in multiple logistic regression models (LC–MS).

Annotated metabolite/signal^a^			Odds ratio	95% CI	*p* value	AUC	Difference from baseline model AUC^b^ (difference, 95%CI)
*Signal*	*Annotation/identification*	*Ontology*					
**HDP**							
13.34_590.3324m/z	Glycochenodeoxycholic acid 3-glucuronide	PDd^c^	5.43	(2.03, 14.52)	0.001	0.954	0.217 (0.132, 0.301)
14.10_507.2291n	N/A	N/A	0.29	(0.12, 0.70)	0.006		
15.97_541.3860m/z	Ganoderiol C	PDd	0.24	(0.11, 0.56)	0.001		
3.07_164.0740m/z	S-ethyl-dl-Homocysteine	PDd	0.14	(0.04, 0.42)	0.001		
13.84_541.2983m/z	Thr-Pro-Pro-Val-Gln	PDc	3.83	(1.87, 7.83)	0.0002		
7.21_327.1227m/z	Gibberellin A87	PDd	5.57	(2.49, 12.44)	< .0001		
15.48_557.3022n		N/A	4.11	(2.03, 8.30)	< .0001		
2.77_285.1021m/z	Phosphonic acid, 1,2-ethanediylbis-, tetraethyl ester	PDd	4.37	(1.89, 10.11)	0.001		
**GH**							
0.59_746.6045n	N/A	N/A	0.34	(0.14, 0.81)	0.015	0.945	0.202 (0.101, 0.303)
8.66_762.1452m/z	N/A	N/A	0.10	(0.01, 0.83)	0.033		
14.20_687.3556m/z	Neomenthol-glucuronide	PDb	4.58	(1.75, 11.98)	0.002		
12.74_412.2842m/z	N/A	N/A	0.12	(0.03, 0.53)	0.006		
4.44_180.1181m/z	Dibenzylamine	PDd	0.31	(0.11, 0.89)	0.029		
7.16_208.1212n	Pilocarpine	PDc	2.74	(1.29, 5.81)	0.009		
11.28_219.1743m/z	2,6-Di-*tert*-butyl-4-hydroxymethylphenol	PDa	2.57	(1.05, 6.32)	0.040		
**PE**							
6.30_477.7721m/z	N/A	N/A	3.82	(1.53, 9.52)	0.004	0.95	0.3362 (0.1711, 0.5013)
11.36_299.2368m/z	Bolasterone	PDa	9.53	(1.79, 50.80)	0.008		
15.48_549.3277n	N/A	N/A	4.36	(1.68, 11.31)	0.002		
5.86_313.1070m/z	Cerasinone	PDb	3.14	(1.46, 6.75)	0.004		
**PTB**							
1.48_269.0264m/z	{3-[(2E)-3-phenylprop-2-enoyl]phenyl}oxidanesulfonic acid	PDd	0.56	(0.35, 0.89)	0.014	0.821	0.157 (0.068, 0.246)
0.77_259.0050m/z	1-Propenyl 1-(1-propenylthio)propyl disulfide	PDd	0.61	(0.39, 0.94)	0.026		
3.07_162.0551m/z	Indole-2-carboxylic acid	PDd	0.57	(0.36, 0.90)	0.017		
15.66_770.4609n	N/A	N/A	2.36	(1.44, 3.87)	0.001		
10.49_471.3162m/z	N/A	N/A	1.82	(1.18, 2.80)	0.007		
**sPTB**							
15.97_203.1430m/z	Anisoxide	PDc	2.06	(1.27, 3.35)	0.003	0.865	0.307 (0.196, 0.419)
4.80_178.0863m/z)	5-[2H-Pyrrol-4-(3H)-ylidenemethyl]-2-furanmethanol	PDc	0.46	(0.24, 0.90)	0.023		
8.66_1.99.1309m/z	Di(propylene glycol) propyl ether	PDc	0.24	(0.10, 0.55)	0.001		
15.66_770.4609n	N/A	PDc	2.64	(1.47, 4.75)	0.001		
5.53_230.0424m/z	5-Phosphoribosylamine	PDc	0.26	(0.11, 0.63)	0.003		
2.77_285.1021m/z	Phosphonic acid, 1,2-ethanediylbis-, tetraethyl ester	PDd	2.11	(1.34, 3.32)	0.001		

HDP, any hypertensive disorder of pregnancy; GH, gestational hypertension; PE, pre-eclampsia, PTB, preterm birth; sPTB, spontaneous preterm birth.

^a^UPLC-HRC-MS signal (signal) with predictive value was described with retention time (RT) and exact mass (m/z, or n). The exact neutral mass (n) for a signal was calculated based on two or more than two adducts that were captured for the same molecule. Signals were identified or annotated via matching to an In-house Experimental Standards Library generated by acquiring data for over 1000 compounds under identical conditions to study samples, as well as to public database (including HMDB, NIST, Metlin). N/A, signal was not matched with any of the libraries or database.

^b^Baseline model includes only covariates. HDP: BMI at first prenatal care visit, illegal drug use in the year before pregnancy, gravidity; GH: BMI at first prenatal care visit, illegal drug use in the year before pregnancy, gravidity; PE: illegal drug use in the year before pregnancy, gravidity; PTB: obesity, and illegal drug use in the year before pregnancy, gravidity; sPTB: gravidity.

^c^Ontology levels: PDa, annotation based on matching with public database via exact mass (MS) and experimental tandem mass (MS/MS), it could be the listed compound, or the isomer or derivatives of the listed compound; PDb, annotation based on matching with public database via MS and predict MS/MS; PDc, annotation for the listed compound based on matching with public database via MS and isotopic similarity or adducts; PDd annotation for listed compound based on matching with public database via MS; N/A, signal was not matched with any of the libraries or database.

Over 246 signals were individually associated with PTB (*p* < 0.1), with 189 metabolites identified or annotated (Table S5); 298 signals were individually associated with sPTB (*p* < 0.1) and 135 metabolites identified or annotated (Table S6). In multiple logistic regression analysis, 5 signals were included in the PTB model, while 6 signals were included in the sPTB model (Table 2), all with similar precision (variance) and effect size (odds ratio). A common signal was retained in both models with a RT at 15.66 min and an exact neutral mass at 770.4609 Da. All of these metabolites were annotated with an evidence bases of PDc or below.

In the NMR analysis (Table 3; unadjusted results in tables S6–S10), bins containing signals that could be derived from asparagine/albumin was associated with HDP (OR 0.17, 95% CI 0.04–0.74), and from asparagine/*N*,*N*-dimethylglycine/trimethylamine were associated with PE (OR 0.16, 95% CI 0.05–0.52). Threonine and urea were associated with reduced risk of PTB and SPTB, respectively, but did not add significantly to the predictive value of the model.

**Table 3:** NMR metabolites associated with HDP and PTB in cross-validated multiple logistic regression model (NMR).

	Odds ratios	95% CI	*p* value	Difference in AUC from baseline model^a^
**HDP**				
Asparagine | Albumin (Lysyl)	0.17	(0.04, 0.74)	0.018	0.0412 (0.002, 0.082)
**GH (no NMR selected)**				
**PE**				
Asparagine | *N*,*N*-Dimethylglycine | Trimethylamine	0.16	(0.05, 0.52)	0.003	0.177 (0.013, 0.341)
**PTB**				
Threonine	0.14	(0.03, 0.78)	0.025	0.031 (− 0.036, 0.099)
**sPTB**				
Urea	0.13	(0.02, 0.94)	0.043	0.064 (− 0.038, 0.167)

Adjustment factors: HDP: BMI at first prenatal care visit, illegal drug use in the year before pregnancy, gravidity; GH: BMI at first prenatal care visit, illegal drug use in the year before pregnancy, gravidity; PE: illegal drug use in the year before pregnancy, gravidity; PTB: obesity, and illegal drug use in the year before pregnancy, gravidity; sPTB: gravidity.

HDP, any hypertensive disorder of pregnancy; GH, gestational hypertension; PE, pre-eclampsia, PTB, preterm birth; sPTB, spontaneous preterm birth; GDM, gestational diabetes mellitus.

^a^Baseline model includes only adjustment variables.

An additional aim of our study was to evaluate the correlation between environmental exposures and pregnancy complications. Over 20 metabolites derived from exogenous compounds were identified or annotated (OL1, OL2a, and OL2b), and over a dozen metabolites that are derived from exogenous exposures differentiated the case–control status (univariable logistic regression analysis, *p* < 0.1). This included metabolites of bisphenols, parabens, phthalates, polyphenol metabolites, and medications (Table S2–S6). Monohexyl phthalate was associated with HDP and GH (Table 4), while salicylamide was associated with PE. (R,S)-*N*-Acetyl-S-(2-hydroxy-3-buten-1-yl)-l-cysteine was associated with reduced odds of sPTB.

**Table 4:** Association between exposure of exogenous chemicals and pregnancy complications by stepwise modeling.

Identified/annotated exogenous metabolite^a^		Odds ratio	95% CI	*p* value	AUC	Odds ratio	95% CI	*p* value	AUC	Difference from baseline model† (difference, 95% CI)
**HDP**	*Single metabolite, adjusted for covariates*^b^					*Stepwise*				
3,4,5-trimethoxybenzaldehyde	OL1^c^	1.42	(0.96, 2.10)	0.081	0.753					
Monohexyl phthalate	OL2a	1.50	(1.06, 2.14)	0.024	0.759	1.50	(1.06, 2.14)	0.024	0.759	0.022 (− 0.028, 0.071)
**GH**										
Monohexyl phthalate	OL2a	1.80	(1.19, 2.73)	0.006	0.790	1.80	(1.19, 2.73)	0.006	0.790	0.047 (− 0.022, 0.116)
**PE**										
Salicylamide	OL2a	1.85	(1.11, 3.09)	0.019	0.749	1.85	(1.11, 3.09)	0.019	0.749	0.135 (− 0.006, 0.276)
Ethylparaben	OL2b	0.58	(0.33, 1.00)	0.05	0.723					
**PTB**										
2,6-Dimethoxyphenol	OL2b	0.70	(0.45, 1.06)	0.093	0.684	^d^				
**sPTB**										
Salicylamide	OL2a	0.70	(0.46, 1.06)	0.090	0.607					
Hydrocinnamic acid	OL1	0.65	(0.40, 1.07)	0.092	0.624					
2,6-Dimethoxyphenol	OL2b	0.63	(0.39, 1.02)	0.060	0.618					
*N*-Heptylparaben	OL2a	0.66	(0.41, 1.05)	0.077	0.584					
(R,S)-*N*-Acetyl-S-(2-hydroxy-3-buten-1-yl)-l-cysteine	OL2a	0.65	(0.43, 0.99)	0.047	0.626	0.65	(0.43, 0.99)	0.047	0.626	0.068 (− 0.035, 0.171)

HDP, any hypertensive disorder of pregnancy; GH, gestational hypertension; PE, pre-eclampsia, PTB, preterm birth; sPTB, spontaneous preterm birth; GDM, gestational diabetes mellitus.

^a^The exogenous metabolites include the exposed parent chemicals and their metabolites or conjugates that are formed in vivo after the exposures. The exogenous metabolite was identified or annotated via matching to an In-house Experimental Standards Library that contains over 300 exogenous metabolite standards, which were prioritized for data acquisition based on previous findings in human exposure studies.

^b^*p* < 0.10 after adjustment for covariates. Single metabolite models include only one metabolite as well as covariates; stepwise models include all predictive metabolites simultaneously and retain those *p* < 0.05.

Adjustment factors: HDP: BMI at first prenatal care visit, illegal drug use in the year before pregnancy, gravidity; GH: BMI at first prenatal care visit, illegal drug use in the year before pregnancy, gravidity; PE: illegal drug use in the year before pregnancy, gravidity; PTB: obesity, and illegal drug use in the year before pregnancy, gravidity; sPTB: gravidity.

^c^Ontology levels: Identification or annotation of exogenous metabolites are supported by evidences from chromatography, e.g., retention time (RT), and/or mass spectrometry, e.g., exact mass (MS) and/or tandem mass spectra (MS/MS). OL1, highly confident identification based on matching with In-house physical standard library (IPSL) via retention time (RT, with RT error ≤|0.5|), exact mass (MS, with mass error < 5 ppm), and tandem mass similarity (MS/MS, with similarity ≥ 30); OL2a, confident identification based on matching with IPSL via MS and RT; OL2b, annotation for the isomer or derivatives of the compound listed, based on matching with IPSL via MS and MS/MS.

^d^No variables *p* < 0.05.

**Figure 1:**
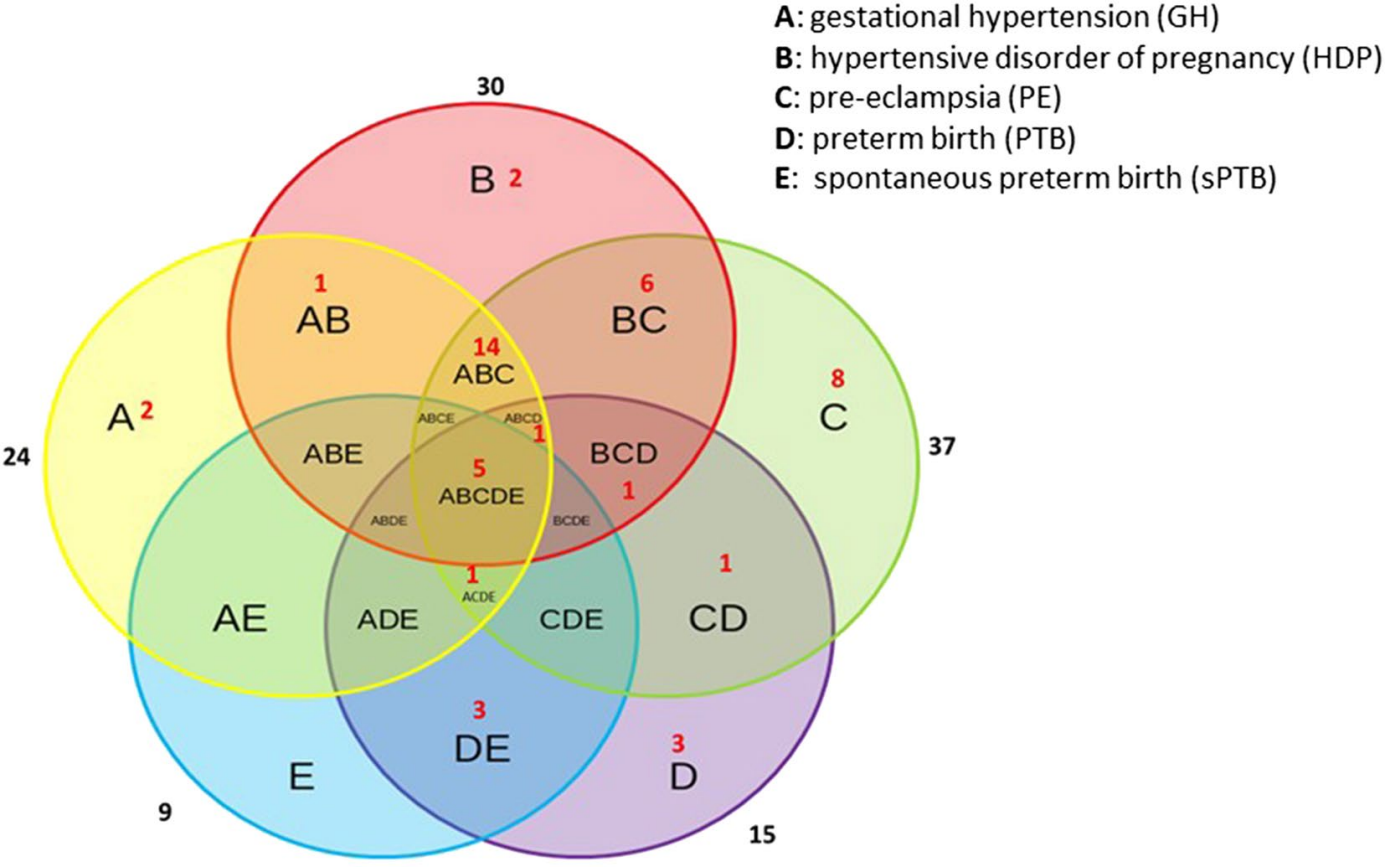
Overlap of pathways by complications under study. Venn diagram of metabolic pathways perturbed between cases (e.g., GH, HPD, PE, PTB, and sPTB) and controls. Pathway enrichment was conducted by Genego Metacore using Enrichment by Pathway Map, and the cut-off for pathway enrichment is *p* < 0.01. Each section of the diagram is labeled by capital letters (A, B, C, D, E), and the numbers of pathways that were specific to a certain phenotype (in the region with single capital letter) or overlapping between different phenotypes (in the region with combination of letters). The list of pathways corresponding to each section are shown in Table 5.

For pathway analysis, metabolites that were perturbed between cases and controls with the evidence bases of OL1 (RT, MS, MS/MS) or OL2a (RT, MS) included individual steroid hormones, acetylcarnitines, nucleosides, hydroxyl short-chain fatty acids, and exogenous metabolites. Thirty pathways were found to be associated with HDP, with 24 associated with GH and 37 associated with PE; while 15 pathways were associated with PTB and 9 with sPTB (Fig. 1 and Table 5). Five perturbed pathways were associated with all the investigated complications: aminoacyl-tRNA biosynthesis, l-threonine, renal secretion of organic electrolytes, and urea cycle. HDP, GH and PE were also highly overlapping in pathways related to cortisol biosynthesis, cholesterol and sphingolipid transport, lipoprotein metabolism, and metabolic syndrome/type 2 diabetes. Pathways associated with PTB and/or sPTB related to cortisol production activation in depression, renal secretion of drugs, transcription role of Vitamin D receptor in regulation of genes involved in osteoporosis, immune responses, and tyrosine metabolism.

**Table 5:** Enriched metabolic pathways perturbed between different case groups and control group (corresponding to Fig. 1).

Region codes^a^	Enriched pathways (cut-off, *p < 0.01)	Outcome	*p* value^b^	Number of identified metabolites perturbed in the pathway	FDR^c^
**ABCDE (5)**	Aminoacyl-tRNA: biosynthesis in cytoplasm	GH	1.9E−06	6/98	1.6E−05
		HDP	7.6E−10	9/98	2.5E−08
		PE	2.8E−13	12/98	1.5E−11
		PTB	4.4E−10	7/98	1.0E−08
		sPTB	9.4E−06	4/98	9.7E−05
	Aminoacyl-tRNA: biosynthesis in mitochondrion	GH	6.1E−07	6/81	6.7E−06
		HDP	1.3E−10	9/81	7.4E−09
		PE	2.6E−14	12/81	2.1E−12
		PTB	1.1E−10	7/81	5.1E−09
		sPTB	4.4E−06	4/81	6.8E−05
	l-Threonine metabolism	GH	3.9E−05	4/50	2.6E−04
		HDP	9.1E−05	4/50	1.1E−03
		PE	1.9E−04	4/50	2.0E−03
		PTB	2.3E−06	4/50	3.4E−05
		sPTB	6.2E−07	4/50	1.9E−05
	Renal secretion of organic electrolytes	GH	2.1E−04	4/77	1.2E−03
		HDP	4.9E−04	4/77	5.1E−03
		PE	1.0E−03	4/77	7.8E−03
		PTB	9.5E−03	2/77	2.9E−02
		sPTB	5.2E−03	2/77	1.8E−02
	Urea cycle	GH	8.4E−03	2/36	2.4E−02
		HDP	2.5E−05	4/36	4.1E−04
		PE	1.8E−06	5/36	3.3E−05
		PTB	2.1E−03	2/36	1.6E−02
		sPTB	1.2E−03	2/36	8.6E−03
**ABCD (1)**	Signal transduction: Amino acid-dependent mTORC1 activation	GH	4.9E−04	4/96	2.1E−03
		HDP	1.1E−03	4/96	9.8E−03
		PE	3.6E−09	9/96	9.9E−08
		PTB	3.1E−05	4/96	3.5E−04
**ACDE (1)**	Leucine, isoleucine and valine metabolism	GH	5.9E−04	3/43	2.2E−03
		PE	1.9E−03	3/43	1.2E−02
		PTB	7.3E−05	3/43	6.6E−04
		sPTB	1.7E−03	2/43	8.6E−03
**ABC (14)**	Aberrant lipid trafficking and metabolism in age-related macular degeneration pathogenesis	GH	1.6E−03	3/60	5.5E−03
		HDP	2.9E−03	3/60	2.2E−02
		PE	3.9E−04	4/60	3.5E−03
	Cholesterol and Sphingolipid transport/distribution to the intracellular membrane compartments (normal and CF)	GH	9.0E−11	7/38	2.0E−09
		HDP	4.3E−10	7/38	1.8E−08
		PE	1.7E−09	7/38	5.6E−08
	Cholesterol and sphingolipid transport/generic schema (normal and CF)	GH	5.6E−13	8/35	1.8E−11
		HDP	3.4E−12	8/35	2.9E−10
		PE	1.7E−11	8/35	6.9E−10
	Cholesterol and sphingolipid transport/influx to the early endosome in lung (normal and CF)	GH	5.5E−03	2/29	1.7E−02
		HDP	8.4E−03	2/29	5.0E−02
		PE	2.2E−05	4/29	3.2E−04
	Cholesterol and sphingolipid transport/recycling to plasma membrane in lung (normal and CF)	GH	7.5E−08	5/29	1.2E−06
		HDP	2.3E−07	5/29	6.3E−06
		PE	5.9E−07	5/29	1.4E−05
	Cholesterol and sphingolipid transport/transport from Golgi and ER to the apical membrane (normal and CF)	GH	2.7E−07	5/37	3.6E−06
		HDP	8.0E−07	5/37	1.9E−05
		PE	2.1E−06	5/37	3.4E−05
	Chylomicron dyslipidemia in type 2 diabetes and metabolic syndrome X	GH	5.5E−04	3/42	2.1E−03
		HDP	4.6E−05	4/42	5.9E−04
		PE	1.8E−03	3/42	1.2E−02
	Cortisol biosynthesis from cholesterol	GH	4.1E−04	3/38	1.9E−03
		HDP	3.1E−05	4/38	4.6E−04
		PE	6.5E−05	4/38	7.1E−04
	HDL dyslipidemia in type 2 diabetes and metabolic syndrome X	GH	4.1E−04	3/38	1.9E−03
		HDP	7.7E−04	3/38	7.6E−03
		PE	1.3E−03	3/38	9.6E−03
	Lipoprotein metabolism	GH	2.3E−03	3/68	7.4E−03
		HDP	1.7E−05	5/68	3.2E−04
		PE	7.1E−03	3/68	3.4E−02
	Metabolic syndrome X (general schema)	GH	2.4E−05	3/15	1.7E−04
		HDP	4.5E−05	3/15	5.9E−04
		PE	1.3E−06	4/15	2.7E−05
	Metabolism of l-proline and derivatives	GH	1.8E−04	4/74	1.1E−03
		HDP	4.2E−04	4/74	4.7E−03
		PE	6.6E−05	5/74	7.1E−04
	Transport: HDL-mediated reverse cholesterol transport	GH	5.5E−04	3/42	2.1E−03
		HDP	1.0E−03	3/42	9.6E−03
		PE	1.8E−03	3/42	1.2E−02
	Transport: intracellular cholesterol transport	GH	1.8E−16	12/83	1.2E−14
		HDP	3.1E−15	12/83	5.1E−13
		PE	2.2E−17	14/83	3.6E−15
**BCD (1)**	Myeloid-derived suppressor cells and M2 macrophages in cancer	HDP	1.3E−05	5/64	2.7E−04
		PE	6.0E−03	3/64	3.0E−02
		PTB	6.7E−03	2/64	2.9E−02
**AB (1)**	Androgen biosynthetic pathways	GH	3.8E−03	3/82	1.2E−02
		HDP	7.0E−03	3/82	4.3E−02
**BC (6)**	Aspartate and asparagine metabolism	HDP	5.1E−03	3/73	3.4E−02
		PE	6.2E−05	5/73	7.1E−04
	Glycine and l-serine metabolism	HDP	3.7E−03	4/133	2.6E−02
		PE	1.0E−03	5/133	7.8E−03
	l-Arginine metabolism	HDP	6.3E−03	3/79	4.1E−02
		PE	1.1E−03	4/79	8.2E−03
	Muscle contraction: regulation of eNOS activity in endothelial cells	HDP	3.7E−03	3/65	2.6E−02
		PE	5.3E−04	4/65	4.5E−03
	Niacin-HDL metabolism	HDP	1.4E−03	3/46	1.1E−02
		PE	2.3E−03	3/46	1.3E−02
	Regulation of lipid metabolism: G-alpha(q) regulation of lipid metabolism	HDP	2.8E−03	3/59	2.2E−02
		PE	4.8E−03	3/59	2.5E−02
**CD (1)**	Immune response: distinct metabolic pathways in naive and effector CD^8+^ T cells	PE	8.9E−03	3/74	3.9E−02
		PTB	8.8E−03	2/74	2.9E−02
**DE (3)**	Activation of cortisol production in major depressive disorder	PTB	2.6E−03	2/40	1.7E−02
		sPTB	1.4E−03	2/40	8.6E−03
	Renal secretion of drugs	PTB	3.6E−03	2/47	2.0E−02
		sPTB	2.0E−03	2/47	8.8E−03
	Transcription role of VDR in regulation of genes involved in osteoporosis	PTB	5.7E−03	2/59	2.8E−02
		sPTB	3.1E−03	2/59	1.2E−02
**A(2)**	Regulation of lipid metabolism: PPAR regulation of lipid metabolism	GH	9.2E−07	5/47	8.7E−06
	Transport regulation of ATP-binding transporters by Retinoic acid and orphan nuclear receptor PXR		9.8E−03	2/39	2.7E−02
**B(2)**	Immune response: The effect of IDO1 on T cell metabolism	HDP	8.8E−03	3/89	5.0E−02
	Putative pathways of oleic acid sensing in ventromedial hypothalamus in obesity		8.9E−03	2/30	5.0E−02
**C(8)**	Antioxidant effects of statins in COPD	PE	5.0E−03	3/60	2.5E−02
	Apoptosis and survival: NO signaling in apoptosis		7.7E−03	2/23	3.6E−02
	Apoptosis and survival: NO signaling in survival		8.4E−03	2/24	3.8E−02
	Beta-alanine metabolism		4.7E−05	4/35	6.3E−04
	Environmental factors-induced inflammatory signaling in normal and asthmatic airway epithelium		1.9E−03	3/43	1.2E−02
	mne-glutamate-glutamine metabolism		2.2E−04	5/95	2.1E−03
	l-Lysine metabolism		2.0E−03	4/93	1.2E−02
	Reactive oxygen and nitrogen species production in eosinophils in asthma		4.8E−03	3/59	2.5E−02
**D(3)**	Catecholamine metabolism	sPTB	9.0E−03	2/75	2.9E−02
	Immune response: T regulatory cell-mediated modulation of antigen-presenting cell functions		7.1E−03	2/66	2.9E−02
	Tyrosine metabolism p.1 (dopamine)		7.9E−03	2/70	2.9E−02

Metabolic pathways between cases (e.g., GH, HDP, PE, PTB, and sPTB) and controls were enriched by GeneGo MetaCore using Enrichment by Pathway Maps.

^a^Capital letters (A, B, C, D, E, and their combinations) and numbers are corresponding to the Venn diagram in Fig. 1. Pathways in a region with single capital letter are specific to a certain phenotype; while pathways in a region with combination of letters indicate the common pathways being impacted in different phenotypes. A, gestational hypertension (GH); B, hypertensive disorder of pregnancy (HDP); C, pre-eclampsia (PE); D, preterm birth (PTB); E, spontaneous preterm birth (sPTB).

^b^The *p* value that was generated from the hypergeometric test in Metacore indicates the significance of enrichment of metabolites in pathway mapping. A lower *p* value indicates a higher significance in the pathway enrichment.

^c^FDR = false discovery rate.

## Discussion

In this untargeted metabolomic analysis of first trimester serum samples, we identified and annotated several endogenous and exogenous metabolites associated with complications of pregnancy, and showed that metabolites significantly improved the predictive value of models over known risk factors. The number of features differentiating cases and controls and the identified/annotated features found for PTB were less than that of HDP; this may indicate that PTB is a more heterogeneous condition. The investigation was is a discovery-based (i.e., untargeted) approach which could lead to biomarker(s) useful in clinical practice. Unlike analyses that focused mainly on a few signals with identification/annotation^13,16,26^, we created models using all signals for a more comprehensive analysis. Some signals used in the modelling approach could be identified through retention time, mass, and fragmentation, while others were annotated through public databases or remained unknown. The identifications and annotations in our study provide evidence-based ontology levels, which is important for data comparison and harmonization in future collaborations.

*Exogenous metabolites* Monohexyl phthalate was correlated with HDP and GH, and phthalate metabolites were weakly associated with decreased blood pressure in the second trimester in one previous study^27^. The correlation between salicylamide and PE may be due to the usage of aspirin-like medication (such as Labetalol, 2-hydroxy-5-[1-hydroxy-2-[(1-methyl-3-phenylpropyl)amino]ethyl]benzamide monohydrochloride), in hypertensive women^28^. (In our study, salicylamide levels were higher for the 5 women in the study, 4 cases and 1 control, who had chronic hypertension.) (R,S)-*N*-Acetyl-S-(2-hydroxy-3-buten-1-yl)-l-cysteine (MHB2) is a metabolite generated in vivo after exposure to 1,3-butadiene via smoking or air pollution^29^; the link we found between MHB2 and sPTB is consistent with previous studies finding associations with these toxicants^30,31^.

*Individual metabolites, HDP*: Our study identified multiple signals with strong predictive value for HDP. We attempted to match signals to our in-house library of standards run under identical conditions to the study samples, as well as with public database. These signals could not be identified using evidence of retention time and/or MS/MS spectra pattern. Therefore, we provided the tentative annotation and chromatographic/spectra information for those important signals, which might be helpful for identification/annotation using other data mining technologies in the future^22,32^. We found a large number of metabolic profiles that were significantly perturbed (*p* < 0.1) between cases and controls (Table S2–S11 in supplementary materials). Although none of these identified/annotated metabolites was predictive enough to be used as a clinical biomarker, most of our findings in metabolic profiles (Table S2–S11) are highly consistent with the New Zealand SCOPE cohort^33^, as well as other discovery-phase studies^34,35^. One of the signals with predictive value for PE matched to an androgen steroid hormone, and the PE-associated perturbation of steroid hormones was also reported in the SCOPE study^33^. Increased androgens are correlated with vascular dysfunction in HDP, interrupting oxygen and nutrient transport from the maternal blood supply^36^. In the GH model, 2,6-Di-*tert*-butyl-4-hydroxymethylphenol (BHT-OH, PDa) was predictive. This compound is a metabolite of 2,6-Di-*tert*-butyl-4-methylphenol (BHA), a synthetic phenolic antioxidant used widely in foods, polymers, and cosmetics to slow oxidation. Some BHA metabolites have been found to induce cellular DNA damage and the chemical was placed on the European Union watch list in 2015^37^. Only elevated acylcarnitine and decreased taurine levels have repeatedly been found to relate to PE in previous metabolomic studies^6^. Neither was included in our final model, but butenylcarnitine and 3-hydroxyhexanoyl carnitine were associated with higher odds of HDP in univariate models (Table S2); no association was found with taurine.

*Individual metabolites, PTB*: Most of the signals retained in the final models for PTB and sPTB were identified with public database matching. Of the metabolites we found that were associated with PTB in this analysis, only threonine had been previously associated with PTB, with a negative association^20^. Our previous review of metabolomics and PTB found little consistency across studies, with only myoinositol, creatinine, histidine, and 5-oxoproline associated across multiple studies^20^. Among these, in our analysis, only histidine was weakly associated with PTB, and it was not retained in final models.

*Common pathways*: Pathways involved in protein synthesis (aminoacyl-tRNA biosynthesis), threonine metabolism, urea cycle, and renal secretion of organic electrolytes were perturbed in both HDP and PTB. Protein synthesis and amino acid metabolism play important roles in maternal and fetal health. Pregnant women who have inherited metabolic disorders in protein and amino acid metabolism are more likely to develop pregnancy complications, indicating burdens in urea nitrogen clearance^38^. A previous study of late-onset pre-eclampsia also found associations with aminoacyl-tRNA synthesis (though they were not statistically robust)^39^. Perturbation of the renal secretion of organic electrolytes pathway may indicate changes in the kidney proximal tubule related to xenobiotic metabolism^40^.

*Pathways and individual complications*: Multiple pathways were perturbed in the early part of pregnancies that later developed HDP. Several lipid-related pathways were associated with HDP, consistent with the disruptions of lipid metabolism that have been demonstrated in HDP^41,42^. The leucine, valine, and isoleucine metabolism, related to both HDP and PTB in these data, was previously associated with late-onset preeclampsia^39^. 4-hydroxyglutamate, identified as a strong predictor of PE in a previous study^16^, was not associated in our analysis. However, it is involved in the arginine-proline metabolism pathway, one of the pathways identified for HDP, and is a substrate that produces 4-hydroxy-2-oxoglutarate, an intermediate on several pathways identified in this analysis. Pathways related to oxidative stress, nitrous oxide signaling, and inflammatory signaling were associated only with PE, suggesting that the oxidative stress and inflammation leading to severe damage in endothelial function might contribute to the more severe pathology of PE. Fewer pathways were associated with PTB and the associations were less strong, but some were intriguing. For instance, the pathways related to activation of cortisol pathways in major depressive disorder were perturbed, and cortisol and depression have both been previously related to PTB^43^.

Strengths of the study include the first-trimester sampling and strong QC for both the sample collection and the spectroscopic analyses. Limitations include the small sample size, lack of detailed information on subtypes of PE and PTB, lack of a replication sample, the single-timepoint sample, and the limited number of African-American participants.

This study contributes to the growing literature on metabolites associated with pregnancy complications and suggests that perturbations of several common pathways are associated with both HDP and PTB. The metabolomic field needs to report the evidence basis for identifications and annotations in order to increase the usability of reported findings.

## Supplementary Information

Supplementary Information.
