# Supplementary material for: Untargeted analysis of first trimester serum to reveal biomarkers of pregnancy complications: a case–control discovery phase study
Source: Sci Rep. 2021 Feb 10;11:3468. doi: 10.1038/s41598-021-82804-1 (PMC7876105; doi:10.1038/s41598-021-82804-1)
Supplement: Supplementary file 1 — Supplementary Information. [file 41598_2021_82804_MOESM1_ESM.docx]

**SUPPLEMENTAL MATERIAL**

Untargeted Analysis of First Trimester Serum to Reveal Biomarkers of Pregnancy Complications: A case-control discovery phase study EW Harville,^1*†^ Y-Y Li,^2^^†^ K Pan,^1^ S McRitchie,^2^ W Pathmasiri,^2^ S Sumner^2*^

1. Department of Epidemiology, Tulane University School of Public Health and Tropical Medicine, New Orleans, LA
2. Nutrition Research Institute, Department of Nutrition, University of North Carolina at Chapel Hill School of Public Health, Chapel Hill, NC 27599, USA

† Co-first authors

*Corresponding Author

Susan CJ Sumner, PhD

Professor, Department of Nutrition

Nutrition Research Institute

University of North Carolina at Chapel Hill

Chapel Hill, NC, 27514, USA

Tel: 919-622-4456

Email: Susan_sumner@unc.edu

* Corresponding Author

EW Harville, PhD

Tulane School of Public Health and Tropical Medicine

Epidemiology #8318

1440 Canal St. Ste. 2001

New Orleans, LA 70112

harville@tulane.edu

Tel. 504-988-7327

Fax. 504-988-1568

**Checklist of Supplementary Data:**

1). **Sample preparation, data acquisition, data preprocessing, metabolite identification and annotation, and statistical analysis for untargeted metabolomics via UPLC-HR-MS and NMR.**

**2). Table S1. Descriptive statistics, overall Global Alliance to Prevent Prematurity and Stillbirth (GAPPS) registry**

**3). Table S2. UPLC-HR-MS determined metabolites/peaks associated with hypertensive disorders in pregnancy (HDP) (p<0.1)**

**4). Table S3. UPLC-HR-MS determined metabolites/peaks associated with gestational hypertension (GH) (p<0.1)**

**5). Table S4. UPLC-HR-MS determined metabolites/peaks determined metabolites/peaks that are associated with preeclampsia (PE) (p<0.1)**

**6). Table S5.** **UPLC-HR-MS determined metabolites/peaks metabolites/peaks that are associated with preterm birth (PTB) (p<0.1)**

**7). Table S6. UPLC-HR-MS determined metabolites/peaks associated with preterm birth (sPTB) (p<0.1)**

**8). Table S7. Nuclear magnetic resonance (NMR)-determined bins, library matched to metabolites associated with hypertensive disorders in pregnancy (HDP) (p<0.1)**

**9). Table S8. Nuclear magnetic resonance (NMR)-determined bins, library-matched to metabolites associated with gestational hypertension (GH) (p<0.1)**

**10). Table S9. Nuclear magnetic resonance (NMR)-determined bins, library matched to metabolites associated with preeclampsia (PE) (p<0.1)**

**11). Table S10. Nuclear magnetic resonance (NMR)-determined bins, library matched to metabolites associated with preterm birth (PTB) (p<0.1)**

**12). Table S11. Nuclear magnetic resonance (NMR)-determined bins, library-matched to metabolites associated with spontaneous preterm birth (SPTB) (p<0.1)**

**1). Sample preparation, data acquisition, data preprocessing and metabolite identification and annotation for untargeted metabolomics via UPLC-HR-MS and NMR.**

**Section A) Experimental methodology for UPLC-HR-MS metabolomics**

*Sample collection*

Serum samples were collected from participants during the first trimester of pregnancy (gestational age range: 6+1-13+6 weeks), with a time from blood draw to completion of specimen processing of less than 2 hours. After processing samples were stored at a minimum of -20°C for short term storage (< 30 days) until shipped to the core repository for storage at -80°C.

*Sample preparation*

Serum samples were prepared according to the published methods ^1, 2^ with modification. 50-µL of serum was mixed with 400-µL methanol containing 500 ng/ml L-tryptophan-d5 and then vortexed at 5,000 rpm for 2 min. Quality control samples (QC pools) were made by pooling 7-µL serum from each of the study sample, and processed with identical procedures as the study sample. All samples (including pools and study samples) were centrifuged at 16, 000 rcf for 5 min at 4°C. The supernatant (350-uL) was dried and reconstituted with 100 µl water-methanol (95:5, v/v) for the UPLC-HR-MS analysis. Study samples were randomized before sample preparation and acquisition with QC pools interspersed.

*Analysis and data acquisition*

The untargeted metabolomics data was acquired on a Vanquish UHPLC system coupled with a Q Exactive HF-X Hybrid Quadrupole-Orbitrap Mass Spectrometer (Thermo Fisher Scientific, San Jose, CA). 5 µl was injected into the instrument for analysis. Metabolites were separated via an HSS T3 C18 column (2.1 x 100 mm, 1.7 µm, Waters Corporation) at 50 °C with mobile phases of water (A) and methanol (B), each containing 0.1% formic acid (v/v). The UHPLC linear gradient began with 2% B, and increased to 100% B in 16 min, then held for 4 min, with a flow rate at 400 µl/min. The untargeted data was acquired from 70 to 1050 m/z under the data dependent acquisition mode.

The UPLC-HR-MS data was processed by Progenesis QI (version 2.1, Waters Corporation) for peak picking, alignment, and normalization. Signals (peaks) that were highly varied (RSD>50%) across QC pools, and the peaks that are significantly differed among running batches (ANOVA, with FDR correction q<0.05), as well as peaks that were with missing value in all QC pools, were excluded for further analysis. Peaks were normalized in Progenesis QI using the “normalize to total intensity” feature.

Peaks were identified or annotated through matching to an in-house experimental standards library (IESL) generated by acquiring data for over 1,000 compounds under identical conditions to study samples. The same set of peaks were also matched to public database, including HMDB, NIST, and METLIN. The evidence that supported each identification or annotation include retention time (RT), exact mass (MS), MS/MS fragmentation pattern, and isotopic ion pattern. Peaks/metabolites that matched to the IESL by a) RT, MS, and MS/MS are labeled as OL1, or b) by RT and MS are labeled OL2a. OL1 and OL2a were considered as confident matches made in this study. An OL2b label was provided for peaks that match by MS and MS/MS to the in-house library but were outside the retention time tolerance (±0.5 min) for the standards run under identical conditions. Peaks matched to public databases are labeled as PDa (MS and experimental MS/MS), PDb (MS and theoretical MS/MS), PDc (MS and isotopic ion pattern), and PDd (MS only).

**Section B) Experimental methodology for NMR metabolomics**

NMR metabolomics was performed according to the published methods^3, 4^ with modification. 400-µL of serum sample was mixed with 300 µL saline (0.9%) containing 2.33 mM formate (chemical shift indicator), and then centrifuged at 16,000 rcf and at 4°C to obtain 600 µL supernatant for NMR analysis. Study QC pools for NMR analysis were made by pooling identical amount of serum from 50 randomly selected study samples, and nine aliquots (400 µL each) were used. QC pools and study samples were processed with identical procedures. All study samples and QC pools were randomized before sample preparation and data acquisition.

1H NMR spectra of serum or plasma samples were acquired on a Bruker Avance III 700 MHz NMR spectrometer equipped with a cryogenically cooled 5 mm ATMA probe at 25 °C. A CPMG pulse sequence with presaturation^5^ was used for data acquisition. For each sample, 128 transients were collected into 64k data points using a spectral width of 12 ppm, 2 s relaxation delay, 400 μs fixed echo time, loop for T2 filter (l4) = 80, and an acquisition time of 3.893 s per FID. The water resonance was suppressed using resonance irradiation during the relaxation delay. NMR spectra were processed using TopSpin software (Bruker, Germany). Spectra were zero filled and Fourier-transformed after exponential multiplication with a line broadening factor of 0.5. Phase and baseline of the spectra were manually corrected for each spectrum. Spectra were referenced internally to the formate signal. The quality of each NMR spectrum was assessed for the level of noise and alignment of identified markers. The NMR spectra were pre-processed using ACD Spectrus Processor 2017 (ACD Labs, Toronto, Canada). NMR bins (0.5 – 8.0 ppm) were made after excluding water (4.5 – 5.0 ppm) using intelligent binning width of 0.04 ppm and 50% looseness factor. Integrals of each of the bins were normalized to the total integral of each of the spectrum. Important NMR bins in the statistical analyses were matched to metabolites using Chenomx NMR Suite 8.4 Professional software (Chenomx, Edmonton, AB, Canada).

**Section C. Statistical analysis**

The 3,122 UPLC-HR-MS peaks create a high dimensional dataset, and the sample size was not sufficient to create a training and test set. Therefore, the 5-fold cross validation procedure was utilized to determine the best model using the R package Caret.^6^ For each pregnancy complication, each of the 5 iterations divided the dataset into 5 groups; one group was selected to be the test set and the other 4 groups were combined to be the training set. Four steps were used to identify the final predictive model for each pregnancy complication. The first step was to select a subset of metabolites that were associated with cases in the univariate logistic regression model in all 5 training sets (p<0.1). The second step was to fit a stepwise multiple logistic regression model on each training set using the metabolites identified in step 1 and all covariates previously identified as important predictors regardless of significance level in the current model, and then evaluating the model using the misclassification rate on each test set. The model with the minimum misclassification rate was determined to be the best model. Step 3 was to identify the number of steps required by the stepwise regression to create the best model in the test set. The final ‘best number of steps” was determined by cross validation error/misclassification. The final cross-validation error of the logistic model with the best number of predictors was 0 for HDP (17 steps), 0 for GH (14 steps), 0 for PE (step=12), 0 for PTB (7 steps), and 0.0968 for sPTB (8 steps). Step 4 was to select the final model by conducting stepwise regression on the full dataset using the best number of steps identified in Step 3. The area under the receiver operating characteristic curve (AUC) was used to evaluate the performance of the prediction models.

**Supplementary tables**

**Table S1. Descriptive statistics, overall Global Alliance to Prevent Prematurity and Stillbirth (GAPPS) registry**

|  | Overall registry (N=2562) |
| --- | --- |
|  | N (%)/Mean (SD) |
| Age | 30.14 (6.3) |
| BMI at first prenatal visit | 28.41 (7.4) |
| Race |  |
| White | 1613 (75.7) |
| Non-White | 166 (7.8) |
| Unknown/rather not say | 351 (16.4) |
| Smoked more than 100 cigarettes (about 5 packs) in your lifetime | 624 (26.0%) |

**Supplementary** **Table S2.** **UPLC-HR-MS determined metabolites/peaks associated with hypertensive disorders in pregnancy (HDP) (univariate logistic regression, p<0.1)**

| **Metabolite/peaks**  **(337 peaks, 173 with annotation or identification)** ^a^ | **Ontology level** ^b^ | **FC**^c^ | ***p*-value**^d^ |
| --- | --- | --- | --- |
| Salicylamide | OL1 | 1.28 | 0.06 |
| Corticosterone | OL1 | 1.36 | 0.09 |
| Cortisol * | OL1 | 1.22 | 0.10 |
| 3,4,5-trimethoxybenzaldehyde* | OL1 | 1.18 | 0.02 |
| Cytidine* | OL1 | 1.49 | <0.01 |
| Cortisone* | OL1 | 1.12 | 0.08 |
| 3-hydroxybutanoate | OL2a | 0.90 | 0.09 |
| Monohexyl phthalate* | OL2a | 1.72 | 0.09 |
| Cortisol derivative or isomer* | OL2a | 1.38 | 0.14 |
| Butenylcarnitine* | OL2a | 1.37 | 0.01 |
| DL-glyceraldehyde | OL2a | 0.91 | 0.05 |
| 3,4-dihydroxyphenylacetate* | OL2b | 1.69 | 0.04 |
| 2',4'-dihydroxyacetophenone | OL2b | 1.18 | 0.08 |
| cortisol* | OL2b | 1.31 | 0.01 |
| 3-hydroxybutanoate | OL2b | 0.87 | 0.04 |
| Val-Val* | PDa | 1.31 | 0.02 |
| Glu Trp* | PDa | 0.71 | 0.06 |
| 4-Androstene-3,17-dione | PDa | 1.46 | 0.05 |
| Pyroglu-Phe | PDa | 1.22 | 0.12 |
| 5-Androsten-3.beta.-ol-17-one | PDa | 1.29 | 0.07 |
| Phe Ser Ala | PDa | 1.83 | 0.06 |
| Bolasterone* | PDa | 1.60 | 0.11 |
| 5-Androsten-3.beta.-ol-17-one* | PDa | 1.74 | 0.01 |
| 17-hydroxyprogesterone* | PDa | 1.47 | <0.01 |
| Cinnamoylglycine | PDa | 0.63 | 0.01 |
| Arg-Phe | PDa | 1.84 | 0.08 |
| Bolasterone* | PDa | 1.36 | 0.14 |
| Retinol / Retinol skeleton* | PDa | 1.50 | <0.01 |
| Trans,trans-Muconic acid | PDa | 0.90 | 0.02 |
| 11-beta-Hydroxyandrosterone-3-glucuronide | PDb | 0.50 | <0.01 |
| 2-butyl-5-[2-(4-hydroxy-3-methoxyphenyl)ethyl]furan | PDb | 1.26 | 0.06 |
| Americanin D* | PDb | 1.26 | 0.02 |
| Hesperetin* | PDb | 1.22 | 0.01 |
| 3,7,8,15-Scirpenetetrol* | PDb | 1.36 | 0.02 |
| 4-Hydroxy duloxetine glucuronide | PDb | 0.62 | 0.05 |
| Cerasinone* | PDb | 1.47 | 0.02 |
| 3-(8,11,14-Pentadecatrienyl)phenol* | PDb | 1.99 | 0.05 |
| 1-a,24R,25-Trihydroxyvitamin D2 | PDb | 0.67 | 0.02 |
| 4-Methoxy-17beta-estradiol | PDb | 1.33 | 0.11 |
| 11-beta-Hydroxyandrosterone-3-glucuronide | PDb | 1.68 | 0.14 |
| Prostaglandin E3 | PDb | 3.26 | 0.08 |
| Cortolone-3-glucuronide | PDb | 1.34 | 0.02 |
| Tetrahydroaldosterone-3-glucuronide | PDb | 1.29 | 0.06 |
| Neomenthol-glucuronide* | PDb | 1.74 | 0.01 |
| Cerasinone* | PDb | 1.46 | <0.01 |
| Betavulgaroside IV* | PDb | 1.46 | 0.03 |
| Glycocholic acid* | PDb | 1.39 | 0.02 |
| P-Hydroxyl-ethotoin* | PDb | 4.07 | 0.30 |
| 12alpha-hydroxy-3-oxo-5beta-cholan-24-oic Acid* | PDb | 0.76 | 0.01 |
| Cortolone-3-glucuronide | PDb | 1.30 | 0.02 |
| Sphingosine 1-phosphate (d16:1-P) | PDb | 1.11 | 0.05 |
| Agaritinal* | PDc | 0.46 | 0.03 |
| L-3-cyanoalanine* | PDc | 1.60 | 0.01 |
| Cis-Acetylacrylate* | PDc | 0.85 | 0.01 |
| 8-hydroxyadenine* | PDc | 1.20 | 0.05 |
| 2-[4-hydroxy-3-(sulfooxy)phenyl]acetic acid | PDc | 0.44 | 0.02 |
| 4,5-dimethyloxazole* | PDc | 1.61 | 0.01 |
| 2-Methylpropyl acetate | PDc | 0.90 | 0.06 |
| Thr-gly | PDc | 0.88 | 0.03 |
| Phosphoserine, butyl ester | PDc | 0.58 | 0.02 |
| 2-(Cyclohexylamino)ethanesulfonic acid | PDc | 1.38 | 0.08 |
| Methionyl-phenylalanine | PDc | 0.46 | 0.02 |
| Glutamyl-hydroxyproline | PDc | 2.27 | 0.05 |
| 5-methoxytryptophan | PDc | 0.68 | 0.02 |
| Leonuriside A | PDc | 1.56 | 0.04 |
| Carbamazepine-O-quinone | PDc | 1.11 | 0.13 |
| Indolylacryloylglycine | PDc | 0.61 | 0.01 |
| Indoleacetyl glutamine | PDc | 1.62 | 0.06 |
| 4-Hydroxycyclohexylcarboxylic acid | PDc | 0.89 | 0.03 |
| Aciclovir | PDc | 1.19 | 0.13 |
| 4-Hydroxyretinoic acid glucuronide | PDc | 1.44 | 0.05 |
| (3,4-Dihydroxyphenyl)ethanol | PDc | 0.91 | 0.05 |
| Ethyl 4-pentenoate | PDc | 0.88 | 0.03 |
| 5-fluorouridine | PDc | 1.22 | 0.03 |
| Cholic acid | PDc | 0.55 | 0.02 |
| N,N-Dimethyl-L-valine | PDc | 0.84 | 0.02 |
| Digoxigenin monodigitoxoside | PDc | 1.72 | 0.12 |
| 1-oleoylglycerophosphoinositol | PDc | 1.27 | 0.09 |
| Val-met* | PDc | 1.56 | 0.10 |
| 6-[6-(1-carboxyethyl)-3-hydroxy-2-(3-methylbut-2-en-1-yl)phenoxy]-3,4,5-trihydroxyoxane-2-carboxylic acid | PDc | 1.34 | 0.03 |
| O-ureidohomoserine | PDc | 0.91 | 0.07 |
| N-Oleoyl tyrosine* | PDc | 1.60 | 0.03 |
| 2-hydroxyxanthone | PDc | 0.79 | 0.13 |
| Pilocarpine* | PDc | 1.37 | <0.01 |
| Neomenthol-glucuronide* | PDc | 2.23 | <0.01 |
| Grepafloxacin | PDc | 1.22 | 0.12 |
| 2-[4-(3-Hydroxypropyl)-2-methoxyphenoxy]-1,3-propanediol 1-glucoside | PDc | 1.57 | 0.12 |
| 3-hydroxyhexanoyl carnitine | PDc | 1.26 | 0.05 |
| 6-[1-(6,7-dimethoxy-2H-1,3-benzodioxol-5-yl)-3-oxopropoxy]-3,4,5-trihydroxyoxane-2-carboxylic acid | PDc | 0.72 | 0.05 |
| 2-Thiouracil | PDc | 0.90 | 0.03 |
| 4-Hydroxycyclohexylcarboxylic acid | PDc | 0.93 | 0.09 |
| 1-(9Z-Octadecenoyl)-sn-glycero-3-phospho-(1'-myo-inositol)* | PDc | 2.21 | <0.01 |
| 2,4-Pentadienal | PDc | 0.93 | 0.07 |
| 11-Oxo-androsterone glucuronide | PDc | 1.43 | 0.05 |
| 4-Ethyl-2-hexylthiazole* | PDc | 1.40 | <0.01 |
| Isolimonic acid* | PDc | 1.55 | 0.10 |
| Travoprost* | PDc | 2.02 | 0.14 |
| 1-(beta-D-Glucopyranosyloxy)-3-octanone | PDc | 0.93 | 0.09 |
| Leucyl-Hydroxyproline* | PDc | 1.34 | 0.05 |
| (+)-Fluprostenol isopropyl ester* | PDc | 2.13 | 0.13 |
| Indole-5,6-quinone | PDc | 0.85 | 0.01 |
| Levulinic acid | PDc | 0.94 | 0.09 |
| Nithiamide | PDc | 0.81 | 0.05 |
| Thr Pro Pro Val Gln* | PDc | 1.95 | <0.01 |
| Indoleacrylic acid | PDd | 0.63 | 0.07 |
| Dehydroascorbic acid | PDd | 0.70 | 0.01 |
| Glycyltyrosine | PDd | 0.77 | 0.09 |
| (+/-)-3-(Ethylthio)butanol | PDd | 0.81 | 0.03 |
| 8-Chloroxanthine* | PDd | 0.81 | 0.01 |
| 8-Methoxykynurenate | PDd | 0.83 | 0.09 |
| DL-Homocysteine, S-ethyl- | PDd | 0.62 | 0.01 |
| 3-[(1E)-1-{4-[2-(dimethylamino)ethoxy]phenyl}-1-(3-hydroxyphenyl)but-1-en-2-yl]phenol | PDd | 0.75 | 0.09 |
| 3-Nonanon-1-yl acetate | PDd | 0.81 | 0.07 |
| (-)-Isopulegol | PDd | 0.91 | 0.09 |
| 7alpha-hydroxy-3-oxochol-4-en-24-oic Acid | PDd | 0.49 | <0.01 |
| Fluconazole | PDd | 1.47 | 0.14 |
| 3,4,5-trihydroxy-6-[4-(5,6,7-trihydroxy-4-oxo-4H-chromen-3-yl)phenoxy]oxane-2-carboxylic acid* | PDd | 1.41 | 0.08 |
| 4,7,8-trihydroxy-2H-chromen-2-one | PDd | 0.84 | 0.02 |
| 3-[3,4-dihydroxy-5-(3,4,5-trihydroxybenzoyloxy)benzoyloxy]-5-hydroxy-4-methoxybenzoic acid* | PDd | 1.43 | 0.02 |
| Ile-Leu* | PDd | 1.58 | 0.04 |
| delta3,5-Deoxytigogenin | PDd | 1.37 | 0.10 |
| Abscisic alcohol | PDd | 1.25 | 0.08 |
| 3-[(2-Methyl-3-furanyl)thio]-4-heptanone* | PDd | 1.53 | 0.02 |
| (Z)-4-Hexenal | PDd | 0.90 | 0.04 |
| 2-Methyl-3-furanthiol* | PDd | 0.82 | 0.02 |
| Pro-Trp-Arg | PDd | 0.79 | 0.03 |
| Diosmetin 7-O-beta-D-glucuronopyranoside | PDd | 1.70 | 0.11 |
| Diguanosine pentaphosphate | PDd | 1.75 | 0.14 |
| Furoparadine* | PDd | 1.22 | 0.05 |
| Cevadine | PDd | 1.81 | 0.09 |
| S-(2,5-Dimethyl-3-furanyl) 2-furancarbothioate* | PDd | 1.50 | 0.02 |
| Octanoylglucuronide | PDd | 0.84 | 0.05 |
| Hydroxyprolyl-Glutamate* | PDd | 1.38 | 0.08 |
| Ser-Phe-Arg | PDd | 2.55 | 0.09 |
| Clemastine | PDd | 1.86 | 0.10 |
| Glycochenodeoxycholic acid 3-glucuronide* | PDd | 2.37 | 0.02 |
| Gibberellin A87* | PDd | 1.43 | 0.01 |
| 2-amino-4-({1-[(carboxymethyl)-C-hydroxycarbonimidoyl]-2-[(2-hydroxy-5-oxo-1,7-diphenylheptyl)sulfanyl]ethyl}-C-hydroxycarbonimidoyl)butanoic acid | PDd | 1.75 | 0.03 |
| 3-Oxo-1,8-octanedicarboxylic acid* | PDd | 0.82 | 0.01 |
| Chalcone* | PDd | 0.81 | 0.01 |
| Dibenzylamine* | PDd | 0.85 | 0.01 |
| cis-5-Dodecenoic acid | PDd | 0.93 | 0.09 |
| Decanenitrile, 10-(methylsulfonyl)-* | PDd | 1.25 | <0.01 |
| 12S-Hydroxy-5Z,8E,10E-heptadecatrienoic acid* | PDd | 1.35 | 0.12 |
| Glucosyl (2E,6E,10x)-10,11-dihydroxy-2,6-farnesadienoate | PDd | 1.29 | 0.11 |
| Muzanzagenin | PDd | 1.19 | 0.14 |
| Aspartyl-Glutamate | PDd | 0.66 | 0.03 |
| Chlorosesamone | PDd | 0.66 | 0.05 |
| Cucurbitacin C* | PDd | 1.60 | <0.01 |
| L-Cysteinylglycine disulfide* | PDd | 1.00 | 0.05 |
| 10-Hydroxy-8-nor-2-fenchanone glucoside | PDd | 1.69 | 0.07 |
| 5-(2-Methylpropyl)tetrahydro-2-oxo-3-furancarboxylic acid | PDd | 0.92 | 0.04 |
| Polysorbate 60* | PDd | 1.10 | 0.09 |
| 2-Phenyl-4-pentenal | PDd | 0.92 | 0.07 |
| 3,6,9,12,15-Pentaoxaheptacosan-1-ol, hydrogen sulfate | PDd | 0.30 | 0.01 |
| Apraclonidine* | PDd | 20.06 | 0.31 |
| Ajoene | PDd | 0.67 | 0.06 |
| Deltoside | PDd | 1.39 | 0.11 |
| Cyclopassifloic acid B | PDd | 1.36 | 0.08 |
| Phosphonic acid, 1,2-ethanediylbis-, tetraethyl ester* | PDd | 1.99 | 0.02 |
| Talinolol | PDd | 1.15 | 0.06 |
| Celiprolol | PDd | 1.15 | 0.06 |
| Leu-Pro-Lys* | PDd | 1.16 | 0.05 |
| 6,7-dihydroxy-5-methoxy-2-phenyl-4H-chromen-4-one | PDd | 1.68 | 0.15 |
| Ganoderiol C* | PDd | 0.78 | 0.02 |
| 8-[3,7-dihydroxy-2-(3-hydroxyphenyl)-3,4-dihydro-2H-1-benzopyran-4-yl]-6-[2-(3,4-dihydroxyphenyl)-3,7-dihydroxy-3,4-dihydro-2H-1-benzopyran-4-yl]-2-(3-hydroxyphenyl)-3,4-dihydro-2H-1-benzopyran-3,5,7-triol | PDd | 1.62 | 0.09 |
| Gln-Met(O)-Lys | PDd | 0.70 | 0.02 |
| Oryzarol* | PDd | 1.39 | <0.01 |
| 8-Methoxykynurenate* | PDd | 1.15 | 0.01 |
| 3beta,7alpha-Dihydroxy-5-cholestenoate* | PDd | 0.82 | <0.01 |
| 2-amino-4-({1-[(carboxymethyl)-C-hydroxycarbonimidoyl]-2-[(2-hydroxy-2-methyl-3-oxo-1-phenylbutyl)sulfanyl]ethyl}-C-hydroxycarbonimidoyl)butanoic acid | PDd | 1.27 | 0.09 |
| Avocadene* | PDd | 0.85 | <0.01 |
| Tyr Pro Thr Val Asn* | PDd | 1.56 | 0.03 |
| 0.51_84.9549m/z | N/A | 0.85 | 0.03 |
| 7.21_131.0688m/z | N/A | 0.75 | 0.02 |
| 8.99_143.0731m/z | N/A | 0.56 | 0.02 |
| 2.20_193.9708m/z* | N/A | 0.80 | 0.01 |
| 8.58_206.0870m/z | N/A | 0.80 | 0.10 |
| 3.34_134.9673m/z* | N/A | 0.86 | 0.03 |
| 2.20_142.9467m/z | N/A | 0.82 | 0.01 |
| 13.66_302.2558m/z* | N/A | 2.18 | 0.02 |
| 3.24_221.5793m/z | N/A | 0.85 | 0.09 |
| 0.88_150.9002m/z* | N/A | 0.78 | <0.01 |
| 6.25_316.1235m/z* | N/A | 1.59 | 0.04 |
| 0.62_90.0497m/z | N/A | 1.21 | 0.06 |
| 2.87_262.9896m/z | N/A | 1.19 | 0.11 |
| 6.32_708.4153n | N/A | 2.02 | 0.05 |
| 3.12_475.2125m/z* | N/A | 0.68 | 0.05 |
| 8.66_762.1452m/z* | N/A | 0.25 | <0.01 |
| 14.02_484.2751m/z | N/A | 0.35 | 0.01 |
| 4.60_237.6678m/z | N/A | 1.33 | 0.09 |
| 8.50_326.0299n | N/A | 0.39 | 0.01 |
| 7.64_443.6270m/z | N/A | 1.34 | 0.13 |
| 3.74_387.7088m/z* | N/A | 2.37 | <0.01 |
| 3.92_237.6370m/z | N/A | 1.93 | 0.09 |
| 5.13_509.2731m/z* | N/A | 0.66 | 0.06 |
| 1.25_134.0049m/z | N/A | 0.83 | 0.03 |
| 9.85_478.5506m/z | N/A | 1.68 | 0.12 |
| 2.20_166.9486m/z | N/A | 0.90 | 0.04 |
| 7.87_279.9912m/z | N/A | 0.89 | 0.09 |
| 4.82_474.6885m/z | N/A | 1.34 | 0.10 |
| 0.81_212.8528m/z | N/A | 0.87 | 0.05 |
| 6.30_477.7721m/z | N/A | 1.69 | 0.12 |
| 3.56_120.9891m/z* | N/A | 0.91 | 0.06 |
| 0.90_98.9979n | N/A | 0.89 | 0.06 |
| 13.23_517.2773m/z | N/A | 0.61 | 0.01 |
| 3.92_107.9718m/z | N/A | 0.89 | 0.05 |
| 8.66_761.8944m/z* | N/A | 0.34 | <0.01 |
| 3.79_344.1927m/z | N/A | 1.90 | 0.07 |
| 9.26_392.1858m/z | N/A | 0.52 | 0.03 |
| 14.30_534.2515n | N/A | 0.52 | 0.02 |
| 3.56_149.9792m/z | N/A | 0.90 | 0.07 |
| 5.00_457.1579m/z | N/A | 1.24 | 0.07 |
| 0.88_200.8769m/z* | N/A | 0.84 | 0.01 |
| 5.20_400.7348m/z | N/A | 1.78 | 0.10 |
| 1.34_146.9745m/z | N/A | 0.90 | 0.04 |
| 7.54_311.2020m/z | N/A | 1.37 | 0.08 |
| 5.92_243.1339m/z* | N/A | 1.99 | <0.01 |
| 1.55_139.9516m/z | N/A | 0.88 | 0.03 |
| 0.77_215.0795n | N/A | 1.39 | 0.05 |
| 5.38_725.8083m/z | N/A | 1.50 | 0.10 |
| 1.23_401.0803m/z | N/A | 1.72 | 0.13 |
| 0.85_226.9339m/z | N/A | 0.84 | 0.07 |
| 0.78_190.8481m/z | N/A | 0.88 | 0.04 |
| 15.09_341.1812n* | N/A | 0.86 | 0.08 |
| 6.27_403.9756m/z | N/A | 0.52 | 0.02 |
| 15.46_274.6348m/z | N/A | 1.17 | 0.08 |
| 10.49_330.0944m/z* | N/A | 1.88 | 0.14 |
| 8.17_572.7879m/z | N/A | 0.48 | 0.02 |
| 6.55_452.9051m/z | N/A | 1.55 | 0.04 |
| 7.64_400.6531m/z | N/A | 1.28 | 0.14 |
| 4.62_398.6247m/z | N/A | 1.59 | 0.14 |
| 7.47_93.0694n* | N/A | 1.19 | 0.01 |
| 12.74_412.2842m/z | N/A | 0.67 | 0.01 |
| 8.17_572.5371m/z | N/A | 0.45 | 0.01 |
| 5.66_579.6482m/z | N/A | 1.79 | 0.05 |
| 8.63_600.1835m/z* | N/A | 1.56 | 0.04 |
| 7.24_676.8623m/z | N/A | 1.71 | 0.14 |
| 0.65_118.0942m/z | N/A | 0.84 | 0.03 |
| 4.30_422.8618m/z | N/A | 1.51 | 0.07 |
| 7.24_676.6116m/z | N/A | 1.87 | 0.10 |
| 11.74_455.3214m/z | N/A | 1.17 | 0.07 |
| 4.44_117.0663n | N/A | 0.91 | 0.05 |
| 4.82_983.3090m/z | N/A | 1.30 | 0.09 |
| 7.21_243.0208n | N/A | 0.87 | 0.05 |
| 0.56_90.9820m/z | N/A | 0.90 | 0.07 |
| 7.66_742.3858n | N/A | 1.42 | 0.08 |
| 0.78_210.8558m/z* | N/A | 0.83 | <0.01 |
| 10.79_367.1872n* | N/A | 1.14 | 0.06 |
| 10.36_1198.6101n | N/A | 1.66 | 0.10 |
| 14.59_557.3296m/z* | N/A | 2.13 | 0.01 |
| 0.65_162.1254m/z* | N/A | 0.82 | 0.01 |
| 6.17_344.6342m/z | N/A | 1.38 | 0.09 |
| 0.88_208.8587m/z | N/A | 0.89 | 0.06 |
| 14.97_1391.9322n | N/A | 1.24 | 0.05 |
| 4.27_322.9156m/z | N/A | 0.91 | 0.03 |
| 11.79_314.1493n | N/A | 1.29 | 0.10 |
| 8.84_824.4202m/z | N/A | 1.47 | 0.11 |
| 8.48_548.0706m/z | N/A | 1.55 | 0.04 |
| 8.12_687.6555m/z | N/A | 1.64 | 0.16 |
| 8.48_487.6750m/z* | N/A | 1.46 | 0.03 |
| 9.51_820.1646m/z | N/A | 1.60 | 0.08 |
| 8.48_547.8199m/z | N/A | 1.54 | 0.07 |
| 8.30_717.0275m/z | N/A | 0.63 | 0.02 |
| 7.66_418.7474m/z | N/A | 1.30 | 0.08 |
| 0.65_329.9232m/z | N/A | 1.29 | 0.05 |
| 8.30_717.1945m/z | N/A | 0.65 | 0.02 |
| 15.54_284.6658m/z | N/A | 1.16 | 0.08 |
| 9.85_1313.6370n* | N/A | 0.66 | 0.05 |
| 0.62_337.9051m/z* | N/A | 1.17 | 0.08 |
| 15.57_139.1091n | N/A | 0.88 | 0.09 |
| 1.16_459.0249m/z | N/A | 0.86 | 0.02 |
| 14.79_536.3016m/z | N/A | 1.14 | 0.05 |
| 14.10_507.2291n* | N/A | 0.73 | 0.01 |
| 5.76_824.4133m/z | N/A | 2.05 | 0.14 |
| 0.69_331.0898n* | N/A | 1.30 | 0.04 |
| 0.59_746.6045n | N/A | 0.74 | 0.02 |
| 6.83_717.6138m/z | N/A | 1.80 | 0.16 |
| 0.56_560.8717m/z* | N/A | 0.69 | 0.01 |
| 6.57_330.6766m/z | N/A | 1.94 | 0.09 |
| 14.74_254.1518n* | N/A | 1.17 | 0.06 |
| 9.09_1022.5013m/z | N/A | 1.72 | 0.14 |
| 14.10_959.3820m/z | N/A | 0.73 | 0.01 |
| 0.54_665.8606m/z | N/A | 0.78 | 0.01 |
| 8.56_860.8294m/z | N/A | 0.74 | 0.05 |
| 8.94_828.6606m/z | N/A | 0.74 | 0.05 |
| 0.54_631.8669m/z | N/A | 0.78 | 0.03 |
| 8.74_816.4079m/z | N/A | 1.51 | 0.10 |
| 14.97_663.9378m/z* | N/A | 1.16 | 0.07 |
| 0.59_501.7514n | N/A | 0.84 | 0.05 |
| 0.56_866.8245m/z* | N/A | 0.78 | 0.03 |
| 0.59_587.7047n | N/A | 0.86 | 0.03 |
| 7.08_522.2842m/z | N/A | 1.45 | 0.07 |
| 15.48_549.3277n* | N/A | 1.08 | 0.06 |
| 1.16_1680.2814n* | N/A | 1.49 | 0.02 |
| 7.71_1047.5955n | N/A | 1.87 | 0.14 |
| 8.56_860.6292m/z | N/A | 0.75 | 0.09 |
| 13.58_543.3973n | N/A | 1.11 | 0.10 |
| 14.10_1016.1106m/z | N/A | 0.81 | 0.03 |
| 1.16_1994.3563n* | N/A | 2.16 | 0.01 |
| 12.59_607.3355n* | N/A | 1.59 | 0.02 |
| 9.64_1043.0000m/z | N/A | 0.85 | 0.06 |
| 14.10_1015.5208m/z | N/A | 0.78 | 0.03 |
| 2.20_140.9488n | N/A | 0.92 | 0.08 |
| 0.54_759.8531m/z | N/A | 0.88 | 0.09 |
| 6.17_650.3145m/z | N/A | 1.37 | 0.10 |
| 7.64_401.6503n | N/A | 1.28 | 0.06 |
| 14.10_1016.0520m/z | N/A | 0.81 | 0.03 |
| 8.35_650.7129m/z | N/A | 1.69 | 0.12 |
| 9.90_932.1363m/z* | N/A | 1.88 | 0.07 |
| 8.53_2282.1461n | N/A | 1.65 | 0.13 |
| 14.12_1015.9932m/z | N/A | 0.83 | 0.03 |
| 4.82_1918.6346n | N/A | 1.31 | 0.10 |
| 5.00_272.0207n* | N/A | 10.11 | 0.30 |
| 0.59_731.6171n | N/A | 0.85 | 0.07 |
| 1.16_692.0937m/z | N/A | 1.20 | 0.03 |
| 0.59_913.4879n | N/A | 0.85 | 0.03 |
| 0.59_618.6671n | N/A | 0.87 | 0.03 |
| 14.12_1015.9340m/z | N/A | 0.82 | 0.05 |
| 13.99_592.1872n* | N/A | 1.41 | 0.05 |
| 0.41_191.9953n | N/A | 0.91 | 0.05 |
| 14.10_1015.8755m/z | N/A | 0.80 | 0.02 |
| 0.59_795.5879n* | N/A | 0.83 | <0.01 |
| 15.48_557.3022n* | N/A | 1.09 | 0.03 |
| 14.10_982.6820n | N/A | 0.82 | 0.03 |
| 14.10_982.7376n | N/A | 0.83 | 0.02 |
| 15.66_770.9636m/z | N/A | 1.09 | 0.05 |
| 0.54_3097.3756n | N/A | 0.87 | 0.02 |
| 15.66_770.4609n* | N/A | 1.09 | 0.03 |
| 4.82_1924.6502n | N/A | 1.34 | 0.13 |
| 15.52_1133.8245n | N/A | 1.10 | 0.07 |
| 0.56_1043.3020m/z | N/A | 0.87 | 0.03 |
| 9.29_1361.5731n | N/A | 1.14 | 0.04 |
| 10.03_2282.2128n* | N/A | 1.15 | 0.05 |
| 0.90_191.0252n | N/A | 1.90 | 0.18 |
| 15.88_404.3160n* | N/A | 0.85 | 0.01 |
| 8.58_1379.5840n | N/A | 1.12 | 0.08 |

^a^Cut-off criterion for the associated peaks/metabolites is p<0.1 without adjusting for covariates; the unidentified or unannotated peaks are listed with retention (RT) and exact mass (m/z or neutral mass). *Metabolites that predict HDP after adjusting for covariates (p<0.1). ^b^Ontology levels: OL1, highly confident identification based on matching with In-house physical standard library (IPSL) via retention time (RT, with RT error≤|0.5|), exact mass (MS, with mass error<5ppm), and tandem mass similarity (MS/MS, with similarity ≥30); OL2a, confident identification based on matching with IPSL via MS and RT; OL2b, annotation for the isomer or derivatives of the compound listed but not the compound itself, based on matching with IPSL via MS and MS/MS; PDa, annotation based on matching with public database via MS and experimental MS/MS (could be the listed compound, or the isomer or derivatives of the listed compound); PDb, annotation based on matching with public database via MS and predict MS/MS; PDc, annotation for the listed compound based on matching with public database via MS and isotopic similarity or adducts; PDd annotation for listed compound based on matching with public database via MS; N/A, peaks was not identified or annotated. ^c^FC, fold change, the ratio of intensity between the HDP subjects vs control, based on the mean, indicates the direction and magnitude of change: FC>1.0 indicates increase compared to control and FC<1.0 indicates decrease compared to control. ^d^ *p*-value determined by logistic modeling.

**Table S3. UPLC-HR-MS determined metabolites/peaks associated with gestational hypertension (GH) (univariate logistic regression, p<0.1)**

| **Metabolite/Peaks**  **(344 peaks, 173 with annotation or identification)** ^a^ | **Ontology level ^b^** | **FC^c^** | ***p*-value^d^** |
| --- | --- | --- | --- |
| Corticosterone | OL1 | 1.37 | 0.08 |
| 3,4,5-trimethoxybenzaldehyde | OL1 | 1.22 | 0.02 |
| Cytidine* | OL1 | 1.35 | 0.01 |
| Bisphenol S | OL1 | 0.87 | 0.04 |
| Glutarate | OL1 | 0.90 | 0.05 |
| 3-hydroxybutanoate | OL2a | 0.85 | 0.03 |
| Monohexyl phthalate* | OL2a | 2.12 | 0.02 |
| L-carnitine* | OL2a | 0.85 | 0.02 |
| Butenylcarnitine* | OL2a | 1.40 | 0.01 |
| Dl-glyceraldehyde | OL2a | 0.88 | 0.04 |
| Dl-glyceraldehyde | OL2a | 0.88 | 0.03 |
| 3,4-dihydroxyphenylacetate* | OL2b | 1.60 | 0.05 |
| 2',4'-dihydroxyacetophenone | OL2b | 1.20 | 0.07 |
| 2-phenylpropionate* | OL2b | 1.26 | 0.02 |
| γ,γ -Dimethylallyl pyrophosphate triammonium salt | OL2b | 2.00 | 0.09 |
| Mevalonate | OL2b | 0.91 | 0.06 |
| Glutarate | OL2b | 0.92 | 0.10 |
| 5-aminopentanoate | OL2b | 0.91 | 0.04 |
| 3-Hydroxy-3-methylglutaric acid | OL2b | 0.90 | 0.07 |
| 4-aminobenzoic acid | PDa | 0.89 | 0.09 |
| Phe Ser Ala | PDa | 1.80 | 0.04 |
| 5-Androsten-3.beta.-ol-17-one | PDa | 1.40 | 0.02 |
| 5-Androsten-3.beta.-ol-17-one | PDa | 1.40 | 0.02 |
| Cinnamoylglycine | PDa | 0.50 | 0.01 |
| 17-hydroxyprogesterone | PDa | 1.51 | <0.01 |
| Retinol / Retinol skeleton* | PDa | 1.78 | <0.01 |
| 17-hydroxyprogesterone | PDa | 1.22 | 0.10 |
| 2-hydroxy-3-methyl-2-Cyclopenten-1-one | PDa | 0.91 | 0.10 |
| 5-Hydroxy-2-methylpyridine | PDa | 1.75 | 0.06 |
| 2,6-Di-tert-butyl-4-hydroxymethylphenol* | PDa | 1.17 | 0.03 |
| Trans,trans-Muconic acid | PDa | 0.88 | 0.03 |
| 11-beta-Hydroxyandrosterone-3-glucuronide | PDb | 0.45 | 0.04 |
| 2-butyl-5-[2-(4-hydroxy-3-methoxyphenyl)ethyl]furan* | PDb | 1.31 | 0.04 |
| 4-Hydroxy duloxetine glucuronide | PDb | 0.49 | 0.05 |
| 3,7,8,15-scirpenetetrol | PDb | 1.36 | 0.05 |
| 3-[2-(3,7-dimethylocta-2,6-dien-1-yl)-3,4-dihydroxyphenyl]propanoic acid | PDb | 3.66 | 0.10 |
| (-)-Matairesinol 4'-[apiosyl-(1->2)-glucoside]* | PDb | 0.68 | 0.09 |
| 11-Oxo-androsterone glucuronide* | PDb | 1.89 | 0.06 |
| 5-Fluorodeoxyuridine monophosphate* | PDb | 1.63 | 0.09 |
| Prostaglandin E3 | PDb | 3.41 | 0.03 |
| Cortolone-3-glucuronide | PDb | 1.44 | 0.01 |
| Tetrahydroaldosterone-3-glucuronide | PDb | 1.38 | 0.04 |
| Cerasinone* | PDb | 1.23 | 0.10 |
| Neomenthol-glucuronide* | PDb | 2.04 | <0.01 |
| Glycocholic acid | PDb | 1.40 | 0.01 |
| Betavulgaroside IV | PDb | 1.59 | 0.04 |
| Cortolone-3-glucuronide | PDb | 1.37 | 0.01 |
| Nitroacetic acid ethyl ester* | PDc | 0.77 | 0.01 |
| L-3-cyanoalanine* | PDc | 1.51 | 0.04 |
| Cis-Acetylacrylate* | PDc | 0.82 | 0.02 |
| 2-Methylpropyl acetate* | PDc | 0.85 | 0.02 |
| Dimethyl 2-oxoglutarate | PDc | 0.89 | 0.09 |
| Thr-Gly* | PDc | 0.85 | 0.02 |
| 4-hydroxy-3-nitrophenylacetate | PDc | 0.82 | 0.04 |
| Nithiamide | PDc | 0.77 | 0.06 |
| Jasmonic acid* | PDc | 2.21 | 0.04 |
| Glutamyl-hydroxyproline | PDc | 2.20 | 0.08 |
| 5-methoxytryptophan* | PDc | 0.63 | 0.09 |
| Argininic acid* | PDc | 0.66 | <0.01 |
| 2-methyl-1,3-thiazolidine-2-carboxamide | PDc | 0.87 | 0.03 |
| 13-Hydroperoxy-9Z,11E-octadecadienoic acid* | PDc | 1.25 | 0.08 |
| Indolylacryloylglycine* | PDc | 0.54 | 0.01 |
| Leonuriside A | PDc | 1.69 | 0.04 |
| N-(4-Acetyl-5-methyl-4,5-dihydro-1,3,4-thiadiazol-2-yl)acetamide | PDc | 1.89 | 0.06 |
| 4-Hydroxycyclohexylcarboxylic acid* | PDc | 0.83 | <0.01 |
| (3e,5z)-1,3,5-heptatriene | PDc | 0.92 | 0.09 |
| Cholic acid | PDc | 0.44 | 0.06 |
| Ethyl 4-pentenoate* | PDc | 0.83 | 0.01 |
| 4-Hydroxyretinoic acid glucuronide* | PDc | 1.63 | 0.01 |
| 5-fluorouridine | PDc | 1.25 | 0.05 |
| Abscisic alcohol 11-glucoside* | PDc | 1.29 | 0.10 |
| (E)-c-hdmapp* | PDc | 0.88 | 0.08 |
| 1-oleoylglycerophosphoinositol | PDc | 1.40 | 0.03 |
| 6-[6-(1-carboxyethyl)-3-hydroxy-2-(3-methylbut-2-en-1-yl)phenoxy]-3,4,5-trihydroxyoxane-2-carboxylic acid | PDc | 1.36 | 0.06 |
| Hexanedioic acid, bis(2-ethylhexyl) ester | PDc | 1.28 | 0.07 |
| 2-hydroxyxanthone | PDc | 0.77 | 0.03 |
| 3-Sulfopyruvic acid | PDc | 0.90 | 0.04 |
| Pilocarpine* | PDc | 1.36 | 0.01 |
| 6-[1-(6,7-dimethoxy-2H-1,3-benzodioxol-5-yl)-3-oxopropoxy]-3,4,5-trihydroxyoxane-2-carboxylic acid | PDc | 0.64 | 0.04 |
| 2-[4-(3-Hydroxypropyl)-2-methoxyphenoxy]-1,3-propanediol 1-glucoside | PDc | 1.47 | 0.07 |
| N-Oleoyl tyrosine* | PDc | 1.82 | 0.01 |
| Neomenthol-glucuronide* | PDc | 2.32 | 0.01 |
| 3-hydroxyhexanoyl carnitine* | PDc | 1.26 | 0.09 |
| 2-thiouracil* | PDc | 0.88 | 0.03 |
| 4-Hydroxycyclohexylcarboxylic acid* | PDc | 0.89 | 0.02 |
| 1-(9Z-Octadecenoyl)-sn-glycero-3-phospho-(1'-myo-inositol)* | PDc | 2.24 | <0.01 |
| 3-Hexenedioic acid* | PDc | 0.88 | 0.04 |
| 2,4-pentadienal | PDc | 0.90 | 0.04 |
| Thr Pro Pro Val Gln* | PDc | 2.05 | <0.01 |
| 2-aminoheptanoate* | PDc | 0.91 | 0.03 |
| Isolimonic acid* | PDc | 1.80 | 0.03 |
| NNAL-N-glucuronide | PDc | 3.17 | 0.07 |
| 1-(beta-D-Glucopyranosyloxy)-3-octanone | PDc | 0.91 | 0.06 |
| Indole-5,6-quinone* | PDc | 0.80 | 0.02 |
| Levulinic acid* | PDc | 0.90 | 0.02 |
| Indoleacrylic acid* | PDd | 0.48 | 0.02 |
| Dehydroascorbic acid* | PDd | 0.61 | 0.01 |
| (+/-)-3-(Ethylthio)butanol* | PDd | 0.78 | 0.03 |
| 8-chloroxanthine* | PDd | 0.82 | 0.04 |
| Apraclonidine | PDd | 1.58 | 0.07 |
| DL-Homocysteine, S-ethyl- | PDd | 0.79 | 0.10 |
| 8-methoxykynurenate* | PDd | 0.57 | 0.06 |
| 13.99_175.0755m/z* | PDd | 1.71 | 0.06 |
| 7alpha-hydroxy-3-oxochol-4-en-24-oic Acid | PDd | 0.42 | 0.03 |
| 3-Nonanon-1-yl acetate | PDd | 0.80 | 0.10 |
| (-)-Isopulegol* | PDd | 0.89 | 0.09 |
| Fluconazole* | PDd | 1.44 | 0.10 |
| 4,7,8-trihydroxy-2H-chromen-2-one | PDd | 0.82 | 0.03 |
| 2-Methyl-4-pentenal | PDd | 0.91 | 0.09 |
| 10.54_197.1172m/z | PDd | 1.39 | 0.08 |
| 3-[3,4-dihydroxy-5-(3,4,5-trihydroxybenzoyloxy)benzoyloxy]-5-hydroxy-4-methoxybenzoic acid | PDd | 1.27 | 0.07 |
| 2-(3-hydroxyprop-1-en-1-yl)-6-methoxyphenol | PDd | 1.57 | 0.07 |
| Propenoylcarnitine | PDd | 1.27 | 0.09 |
| N-lactoyl-Tryptophan | PDd | 0.71 | 0.08 |
| Ile-Leu | PDd | 1.58 | 0.06 |
| 3-[(2-Methyl-3-furanyl)thio]-4-heptanone | PDd | 1.36 | 0.06 |
| Abscisic alcohol* | PDd | 1.33 | 0.05 |
| (Z)-4-Hexenal* | PDd | 0.88 | 0.04 |
| 2-Methyl-3-furanthiol* | PDd | 0.75 | 0.05 |
| delta3,5-Deoxytigogenin* | PDd | 1.71 | 0.01 |
| 5(Z),8(Z),11(Z)-Eicosatrienoic acid methyl ester | PDd | 1.23 | 0.08 |
| Amyl 2-furoate* | PDd | 1.22 | 0.03 |
| Furoparadine | PDd | 1.19 | 0.08 |
| Hexylbenzene | PDd | 1.17 | 0.08 |
| Gibberellin A87* | PDd | 1.40 | 0.03 |
| Ser-Phe-Arg | PDd | 3.33 | 0.03 |
| 3-Oxo-1,8-octanedicarboxylic acid* | PDd | 0.80 | 0.03 |
| cis-5-Dodecenoic acid* | PDd | 0.87 | 0.01 |
| Chalcone* | PDd | 0.80 | 0.02 |
| Dibenzylamine* | PDd | 0.81 | <0.01 |
| Glycochenodeoxycholic acid 3-glucuronide* | PDd | 2.77 | 0.01 |
| 2-amino-4-({1-[(carboxymethyl)-C-hydroxycarbonimidoyl]-2-[(2-hydroxy-5-oxo-1,7-diphenylheptyl)sulfanyl]ethyl}-C-hydroxycarbonimidoyl)butanoic acid | PDd | 2.08 | 0.01 |
| 12S-Hydroxy-5Z,8E,10E-heptadecatrienoic acid* | PDd | 1.48 | 0.04 |
| Chlorosesamone | PDd | 0.66 | 0.09 |
| Glucosyl (2E,6E,10x)-10,11-dihydroxy-2,6-farnesadienoate | PDd | 1.31 | 0.06 |
| Aspartyl-Glutamate | PDd | 0.68 | 0.08 |
| Muzanzagenin | PDd | 1.23 | 0.08 |
| Cucurbitacin C* | PDd | 1.63 | <0.01 |
| 5-(2-Methylpropyl)tetrahydro-2-oxo-3-furancarboxylic acid* | PDd | 0.87 | 0.01 |
| Unoprostone | PDd | 1.25 | 0.07 |
| 10-Hydroxy-8-nor-2-fenchanone glucoside | PDd | 1.80 | 0.09 |
| 2'-Hydroxyacetanilide | PDd | 0.90 | 0.05 |
| 3,3'-Dithiobis[4,5-dihydro-2-methylfuran] | PDd | 2.16 | 0.06 |
| Polysorbate 60* | PDd | 1.16 | 0.03 |
| (-)-Carveol* | PDd | 1.15 | 0.06 |
| Phosphonic acid, 1,2-ethanediylbis-, tetraethyl ester* | PDd | 1.75 | 0.03 |
| Talinolol* | PDd | 1.18 | 0.02 |
| Cyclopassifloic acid B | PDd | 1.47 | 0.04 |
| Celiprolol | PDd | 1.16 | 0.06 |
| Chalcone | PDd | 0.91 | 0.10 |
| Leu-Pro-Lys | PDd | 1.20 | 0.01 |
| Gln-Met(O)-Lys* | PDd | 0.62 | 0.03 |
| Oryzarol* | PDd | 1.45 | <0.01 |
| 15-Ketoprostaglandin E2* | PDd | 1.27 | 0.03 |
| 8-Methoxykynurenate | PDd | 1.12 | 0.08 |
| 2-amino-4-({1-[(carboxymethyl)-C-hydroxycarbonimidoyl]-2-[(2-hydroxy-2-methyl-3-oxo-1-phenylbutyl)sulfanyl]ethyl}-C-hydroxycarbonimidoyl)butanoic acid | PDd | 1.32 | 0.06 |
| Avocadene | PDd | 0.88 | 0.08 |
| LysoPC(22:5(4Z,7Z,10Z,13Z,16Z))* | PDd | 0.77 | 0.05 |
| 2.89_116.0425m/z | N/A | 0.80 | 0.10 |
| 0.51_84.9549m/z* | N/A | 0.78 | 0.02 |
| 7.21_131.0688m/z* | N/A | 0.69 | 0.04 |
| 8.99_143.0731m/z | N/A | 0.57 | 0.07 |
| 10.92_110.1047m/z | N/A | 1.43 | 0.10 |
| 1.92_243.0112m/z* | N/A | 0.66 | 0.04 |
| 1.48_103.0584m/z | N/A | 0.86 | 0.07 |
| 2.20_193.9708m/z* | N/A | 0.82 | 0.07 |
| 3.34_134.9673m/z* | N/A | 0.85 | 0.06 |
| 1.71_104.0424m/z | N/A | 0.85 | 0.07 |
| 2.20_142.9467m/z* | N/A | 0.79 | 0.02 |
| 0.88_150.9002m/z* | N/A | 0.73 | <0.01 |
| 3.59_139.9993m/z* | N/A | 0.85 | 0.04 |
| 4.92_205.0385m/z* | N/A | 0.87 | 0.10 |
| 8.66_762.1452m/z* | N/A | 0.19 | 0.02 |
| 2.57_152.0646m/z | N/A | 0.88 | 0.07 |
| 5.10_397.1955m/z* | N/A | 0.63 | 0.07 |
| 1.50_125.0297m/z | N/A | 0.74 | 0.10 |
| 1.89_211.9740m/z* | N/A | 0.68 | 0.09 |
| 1.46_100.0475m/z* | N/A | 0.85 | 0.02 |
| 1.78_177.1144m/z* | N/A | 1.44 | 0.10 |
| 3.12_388.6564m/z* | N/A | 1.62 | 0.06 |
| 3.94_214.9862m/z | N/A | 2.40 | 0.10 |
| 2.35_81.0700m/z | N/A | 0.91 | 0.10 |
| 3.74_387.7088m/z* | N/A | 2.26 | <0.01 |
| 5.08_362.1108m/z* | N/A | 0.68 | 0.07 |
| 15.13_267.6555m/z* | N/A | 1.32 | 0.05 |
| 8.40_269.0863m/z | N/A | 0.73 | 0.06 |
| 5.13_509.2731m/z* | N/A | 0.58 | 0.08 |
| 12.51_159.1169m/z* | N/A | 1.20 | 0.10 |
| 0.18_95.9514m/z | N/A | 0.92 | 0.09 |
| 0.75_234.0824m/z | N/A | 0.75 | 0.08 |
| 1.73_191.0227m/z | N/A | 0.88 | 0.07 |
| 9.72_694.2986m/z | N/A | 0.46 | 0.08 |
| 1.25_134.0049m/z* | N/A | 0.78 | 0.08 |
| 2.20_166.9486m/z | N/A | 0.88 | 0.03 |
| 0.81_212.8528m/z* | N/A | 0.80 | <0.01 |
| 15.97_251.1619m/z | N/A | 1.22 | 0.05 |
| 4.72_128.0424m/z | N/A | 0.83 | 0.08 |
| 3.24_314.8189m/z* | N/A | 0.68 | 0.08 |
| 0.61_80.9524m/z* | N/A | 0.86 | 0.03 |
| 13.23_517.2773m/z | N/A | 0.56 | 0.04 |
| 3.56_120.9891m/z* | N/A | 0.86 | 0.02 |
| 14.30_534.2515n | N/A | 0.44 | 0.07 |
| 0.90_98.9979n* | N/A | 0.86 | 0.09 |
| 4.82_474.6885m/z* | N/A | 1.44 | 0.06 |
| 8.66_761.8944m/z* | N/A | 0.32 | 0.03 |
| 3.92_107.9718m/z | N/A | 0.89 | 0.09 |
| 0.83_306.9575m/z | N/A | 0.79 | 0.04 |
| 1.23_196.0184n | N/A | 1.56 | 0.04 |
| 3.56_149.9792m/z | N/A | 0.89 | 0.08 |
| 0.88_200.8769m/z* | N/A | 0.79 | <0.01 |
| 1.21_152.9975m/z | N/A | 0.89 | 0.08 |
| 5.92_243.1339m/z* | N/A | 1.56 | 0.02 |
| 11.10_292.6349m/z | N/A | 0.52 | 0.08 |
| 1.34_146.9745m/z* | N/A | 0.88 | 0.02 |
| 0.78_202.8563m/z | N/A | 0.84 | 0.05 |
| 8.17_572.7879m/z* | N/A | 0.36 | 0.04 |
| 8.17_572.5371m/z* | N/A | 0.32 | 0.04 |
| 1.23_401.0803m/z* | N/A | 1.57 | 0.08 |
| 2.40_93.9560m/z | N/A | 0.91 | 0.06 |
| 1.55_139.9516m/z | N/A | 0.87 | 0.04 |
| 0.78_190.8481m/z* | N/A | 0.84 | 0.01 |
| 13.41_326.1719n | N/A | 1.44 | 0.10 |
| 12.87_551.3066n | N/A | 0.61 | 0.06 |
| 12.74_412.2842m/z* | N/A | 0.61 | 0.03 |
| 7.47_93.0694n* | N/A | 1.21 | 0.01 |
| 15.13_273.6276m/z | N/A | 1.31 | 0.05 |
| 15.13_274.6633m/z* | N/A | 1.24 | 0.05 |
| 12.53_764.4238m/z | N/A | 0.61 | 0.10 |
| 15.97_237.0791m/z | N/A | 1.17 | 0.09 |
| 0.65_118.0942m/z | N/A | 0.85 | 0.10 |
| 13.99_516.1444m/z | N/A | 1.57 | 0.10 |
| 7.21_535.3096m/z* | N/A | 0.64 | 0.09 |
| 8.33_576.7087m/z* | N/A | 0.66 | 0.03 |
| 4.44_117.0663n* | N/A | 0.88 | 0.02 |
| 5.00_272.0207n* | N/A | 1.29 | 0.05 |
| 1.89_168.0174n* | N/A | 0.75 | 0.03 |
| 7.21_243.0208n | N/A | 0.85 | 0.05 |
| 0.56_90.9820m/z | N/A | 0.87 | 0.04 |
| 15.13_258.6503m/z* | N/A | 1.26 | 0.03 |
| 14.59_557.3296m/z | N/A | 1.87 | 0.01 |
| 9.11_243.0837n* | N/A | 1.58 | 0.07 |
| 4.82_983.3090m/z | N/A | 1.41 | 0.06 |
| 4.82_1956.5825n* | N/A | 1.45 | 0.05 |
| 0.78_210.8558m/z* | N/A | 0.80 | <0.01 |
| 0.88_208.8587m/z* | N/A | 0.84 | 0.01 |
| 0.65_162.1254m/z* | N/A | 0.84 | 0.06 |
| 4.27_322.9156m/z* | N/A | 0.87 | 0.01 |
| 2.54_146.9745m/z* | N/A | 0.90 | 0.05 |
| 14.97_1391.9322n | N/A | 1.23 | 0.09 |
| 0.77_338.7962m/z | N/A | 0.78 | 0.01 |
| 11.79_314.1493n | N/A | 1.32 | 0.06 |
| 8.56_861.0300m/z | N/A | 0.64 | 0.06 |
| 9.85_1313.6370n* | N/A | 0.55 | 0.07 |
| 8.30_717.0275m/z | N/A | 0.60 | 0.07 |
| 0.13_177.9928m/z | N/A | 0.92 | 0.08 |
| 0.65_329.9232m/z | N/A | 1.20 | 0.10 |
| 8.30_717.1945m/z | N/A | 0.61 | 0.07 |
| 8.30_860.6838m/z* | N/A | 0.68 | 0.10 |
| 8.30_860.5171m/z* | N/A | 0.60 | 0.02 |
| 9.72_688.9753m/z | N/A | 0.61 | 0.10 |
| 2.20_181.9509m/z* | N/A | 0.90 | 0.05 |
| 14.10_966.3291m/z* | N/A | 0.77 | 0.09 |
| 0.61_131.8962n* | N/A | 0.74 | 0.03 |
| 1.16_459.0249m/z* | N/A | 0.83 | 0.02 |
| 7.64_185.0576m/z | N/A | 0.91 | 0.07 |
| 13.68_954.2641m/z | N/A | 0.69 | 0.05 |
| 0.59_746.6045n* | N/A | 0.64 | 0.01 |
| 0.54_270.9775m/z | N/A | 0.89 | 0.07 |
| 0.73_142.9007m/z* | N/A | 0.87 | 0.02 |
| 14.79_536.3016m/z | N/A | 1.18 | 0.03 |
| 2.30_308.8998m/z | N/A | 0.90 | 0.05 |
| 1.10_150.0698m/z* | N/A | 0.81 | 0.03 |
| 3.22_177.9928m/z* | N/A | 0.89 | 0.03 |
| 13.68_954.0415m/z | N/A | 0.71 | 0.05 |
| 7.69_558.2435m/z* | N/A | 0.81 | 0.06 |
| 10.49_471.3162m/z | N/A | 1.16 | 0.05 |
| 5.53_219.0535n* | N/A | 0.91 | 0.04 |
| 14.48_539.3586n | N/A | 0.73 | 0.01 |
| 0.77_282.8347m/z* | N/A | 0.84 | 0.01 |
| 14.10_959.3820m/z* | N/A | 0.67 | 0.02 |
| 0.56_560.8717m/z | N/A | 0.65 | 0.04 |
| 8.56_860.8294m/z | N/A | 0.67 | 0.06 |
| 15.13_593.2339n | N/A | 1.30 | 0.05 |
| 14.74_254.1518n* | N/A | 1.26 | 0.02 |
| 0.59_501.7514n | N/A | 0.81 | 0.05 |
| 14.97_663.9378m/z | N/A | 1.16 | 0.09 |
| 0.59_587.7047n* | N/A | 0.84 | 0.04 |
| 0.61_453.7681n | N/A | 0.88 | 0.06 |
| 2.54_294.9207m/z | N/A | 0.91 | 0.07 |
| 8.56_860.6292m/z | N/A | 0.70 | 0.09 |
| 14.10_1016.1106m/z* | N/A | 0.75 | 0.01 |
| 1.16_1994.3563n | N/A | 2.07 | <0.01 |
| 14.10_1015.5208m/z | N/A | 0.69 | 0.01 |
| 9.64_1043.0000m/z* | N/A | 0.81 | 0.04 |
| 2.20_140.9488n | N/A | 0.89 | 0.03 |
| 12.59_607.3355n* | N/A | 1.83 | <0.01 |
| 14.10_966.4410m/z* | N/A | 0.78 | 0.05 |
| 0.54_759.8531m/z* | N/A | 0.87 | 0.09 |
| 0.54_589.8853m/z | N/A | 0.88 | 0.07 |
| 13.68_967.7112m/z | N/A | 0.82 | 0.09 |
| 14.10_1016.0520m/z | N/A | 0.79 | 0.04 |
| 0.56_1004.7873m/z* | N/A | 0.82 | 0.05 |
| 15.42_520.5582m/z | N/A | 0.83 | 0.09 |
| 14.12_1015.9932m/z* | N/A | 0.75 | 0.02 |
| 0.59_718.6199n | N/A | 0.83 | 0.07 |
| 8.58_1206.5067n* | N/A | 0.76 | 0.04 |
| 4.82_1918.6346n | N/A | 1.38 | 0.06 |
| 0.59_731.6171n | N/A | 0.82 | 0.06 |
| 0.59_913.4879n | N/A | 0.83 | 0.04 |
| 14.12_1015.9340m/z* | N/A | 0.73 | 0.01 |
| 0.59_870.5135n* | N/A | 0.87 | 0.08 |
| 3.59_177.9929m/z* | N/A | 0.91 | 0.04 |
| 0.59_618.6671n | N/A | 0.87 | 0.06 |
| 15.84_1000.6480n | N/A | 0.66 | 0.08 |
| 14.10_1015.8755m/z* | N/A | 0.71 | 0.01 |
| 0.41_191.9953n* | N/A | 0.87 | 0.02 |
| 13.99_592.1872n* | N/A | 1.50 | 0.06 |
| 0.59_795.5879n* | N/A | 0.80 | 0.01 |
| 0.59_821.5590m/z | N/A | 0.88 | 0.09 |
| 14.10_982.6820n* | N/A | 0.75 | 0.01 |
| 8.25_390.6291m/z* | N/A | 0.86 | 0.09 |
| 15.48_557.3022n* | N/A | 1.09 | 0.05 |
| 14.10_982.7376n* | N/A | 0.75 | 0.01 |
| 0.56_939.7901n | N/A | 0.85 | 0.07 |
| 4.62_177.9929m/z | N/A | 0.92 | 0.06 |
| 15.64_275.6328m/z* | N/A | 1.13 | 0.07 |
| 0.56_1016.7937m/z* | N/A | 0.88 | 0.08 |
| 14.10_990.3083n | N/A | 0.85 | 0.10 |
| 0.56_929.7849n | N/A | 0.87 | 0.07 |
| 4.82_1924.6502n | N/A | 1.47 | 0.05 |
| 15.52_1133.8245n | N/A | 1.12 | 0.08 |
| 2.18_177.9562m/z | N/A | 0.91 | 0.05 |
| 7.69_1054.5439n* | N/A | 0.85 | 0.09 |
| 13.68_965.4919m/z | N/A | 0.83 | 0.07 |
| 0.90_191.0252n | N/A | 1.24 | 0.10 |
| 0.56_1043.3020m/z* | N/A | 0.86 | 0.04 |
| 0.08_208.9753n | N/A | 0.93 | 0.09 |
| 2.42_191.9721m/z* | N/A | 0.91 | 0.04 |
| 8.02_1615.6513n* | N/A | 0.66 | 0.01 |
| 15.88_404.3160n* | N/A | 0.86 | 0.06 |
| 12.04_355.2628m/z | N/A | 0.27 | 0.09 |
| 1.80_167.0701m/z | N/A | 0.84 | 0.08 |
| 3.66_95.0128m/z | N/A | 3.51 | 0.07 |
| 13.99_175.0755m/z* | N/A | 1.71 | 0.06 |
| 10.39_231.0628m/z | N/A | 1.27 | 0.08 |

^a^Cut-off criterion for the associated peaks/metabolites is p<0.1 without adjusting for covariates; the unidentified or unannotated peaks are listed with retention (RT) and exact mass (m/z or neutral mass). *Metabolites that predict GH after adjusting for covariates (p<0.1). ^b^ Ontology levels: OL1, highly confident identification based on matching with In-house physical standard library (IPSL) via retention time (RT, with RT error≤|0.5|), exact mass (MS, with mass error<5ppm), and tandem mass similarity (MS/MS, with similarity≥30); OL2a, confident identification based on matching with IPSL via MS and RT; OL2b, annotation for the isomer or derivatives of the compound listed but not the compound itself, based on matching with IPSL via MS and MS/MS; PDa, annotation based on matching with public database via MS and experimental MS/MS (could be the listed compound, or the isomer or derivatives of the listed compound); PDb, annotation based on matching with public database via MS and predict MS/MS; PDc, annotation for the listed compound based on matching with public database via MS and isotopic similarity or adducts; PDd annotation for listed compound based on matching with public database via MS; N/A, peaks was not identified or annotated. ^c^FC, fold change, the ratio of intensity between the GH subjects vs control, based on the mean, indicates the direction and magnitude of change: FC>1.0 indicates increase compared to control and FC<1.0 indicates decrease compared to control. ^d^ *p*-value determined by logistic modeling.

**Table S4. UPLC-HR-MS determined metabolites/peaks that are associated with preeclampsia (PE) (univariate logistic regression, p<0.1)**

| **Metabolite/peak**  **(446 peaks, 189 with annotation or identification)** ^a^ | **Ontology level ^b^** | **FC^c^** | ***p*-value^d^** |
| --- | --- | --- | --- |
| Hydrocinnamic acid* | OL1 | 0.63 | 0.09 |
| Cortisol* | OL1 | 1.60 | 0.01 |
| Cytidine* | OL1 | 1.75 | <0.01 |
| Cortisone* | OL1 | 1.29 | 0.01 |
| Anserine* | OL2a | 0.56 | 0.08 |
| Salicylamide* | OL2a | 1.34 | 0.08 |
| Butenylcarnitine* | OL2a | 1.31 | 0.07 |
| S-carboxymethylcysteine* | OL2a | 1.45 | 0.03 |
| Cortisol* | OL2a | 1.75 | 0.06 |
| N-heptylparaben* | OL2a | 0.80 | 0.10 |
| 3,4-dihydroxyphenylacetate* | OL2b | 1.85 | 0.05 |
| Cortisol* | OL2b | 1.67 | <0.01 |
| N-acetylserotonin* | OL2b | 1.96 | 0.05 |
| Dimethylallyl pyrophosphate* | OL2b | 1.53 | 0.01 |
| 4-aminobenzoic acid* | OL2b | 1.34 | 0.07 |
| Ethylparaben* | OL2b | 0.80 | 0.08 |
| Nornicotine | OL2b | 1.39 | 0.10 |
| 4-Androstene-3,17-dione* | PDa | 1.92 | 0.03 |
| Bolasterone* | PDa | 2.90 | <0.01 |
| 5-Androsten-3.beta.-ol-17-one* | PDa | 2.37 | 0.01 |
| Arg-Phe* | PDa | 2.89 | 0.03 |
| Bolasterone* | PDa | 2.24 | <0.01 |
| Val Val* | PDa | 1.42 | 0.05 |
| 17-hydroxyprogesterone* | PDa | 1.38 | 0.07 |
| 3-Cyano-4,7-dimethylcoumarin* | PDa | 1.60 | 0.05 |
| Uric acid, 1,3-dimethyl-* | PDa | 1.73 | 0.03 |
| 4-aminobenzoic acid* | PDa | 1.16 | 0.09 |
| Isoalantolactone* | PDa | 0.80 | 0.09 |
| Asn Ile Leu* | PDa | 1.63 | 0.09 |
| 9,12-Octadecadiynoic Acid* | PDa | 0.87 | 0.10 |
| Met Arg* | PDa | 1.94 | 0.03 |
| His Phe Trp* | PDa | 1.55 | 0.03 |
| Pyro-Glu-Phe | PDa | 1.31 | 0.09 |
| Phenyl glucuronide | PDa | 2.78 | 0.07 |
| Phe Gln | PDa | 1.41 | 0.10 |
| Pro Thr | PDa | 1.60 | 0.09 |
| Hesperetin* | PDb | 1.36 | 0.01 |
| Americanin D* | PDb | 1.52 | 0.01 |
| 1-a,24R,25-Trihydroxyvitamin D2* | PDb | 0.50 | 0.04 |
| Cerasinone* | PDb | 1.88 | <0.01 |
| 3-(8,11,14-Pentadecatrienyl)phenol* | PDb | 3.58 | <0.01 |
| 11-beta-Hydroxyandrosterone-3-glucuronide* | PDb | 3.02 | 0.01 |
| Glycocholic acid* | PDb | 1.37 | 0.07 |
| Cerasinone* | PDb | 1.89 | <0.01 |
| 12alpha-hydroxy-3-oxo-5beta-cholan-24-oic Acid* | PDb | 0.67 | 0.06 |
| p-Hydroxyl-ethotoin* | PDb | 9.54 | 0.04 |
| [6]-Gingerdiol 3,5-diacetate* | PDb | 1.84 | 0.02 |
| Cerasinone* | PDb | 1.73 | 0.04 |
| 2-[4-(3-Hydroxypropyl)-2-methoxyphenoxy]-1,3-propanediol 1-glucoside* | PDb | 1.56 | 0.05 |
| Succinyladenosine* | PDb | 1.27 | 0.04 |
| Dihydrozeatin-7-N-dihydrozeatin* | PDb | 1.18 | 0.07 |
| LysoPE(22:5(7Z,10Z,13Z,16Z,19Z)/0:0)* | PDb | 1.44 | 0.02 |
| LysoPE(22:6(4Z,7Z,10Z,13Z,16Z,19Z)/0:0)* | PDb | 1.19 | 0.07 |
| Asparaginyl-Lysine | PDb | 0.49 | 0.10 |
| 8,11-Heptadecadienal | PDb | 0.13 | 0.09 |
| L-3-Cyanoalanine* | PDc | 1.77 | 0.01 |
| 8-Hydroxyadenine* | PDc | 1.31 | 0.04 |
| 4,5-Dimethyloxazole* | PDc | 2.11 | <0.01 |
| Pregabalin* | PDc | 1.43 | 0.07 |
| 2-(Cyclohexylamino)ethanesulfonic acid* | PDc | 1.73 | 0.01 |
| Carbamazepine-O-quinone* | PDc | 1.24 | 0.04 |
| 5-[2H-Pyrrol-4-(3H)-ylidenemethyl]-2-furanmethanol* | PDc | 1.32 | 0.04 |
| Aciclovir* | PDc | 1.37 | 0.03 |
| Indoleacetyl glutamine* | PDc | 2.28 | 0.01 |
| Val-Met* | PDc | 1.94 | 0.04 |
| Pilocarpine* | PDc | 1.37 | 0.03 |
| Neomenthol-glucuronide* | PDc | 2.08 | 0.02 |
| Digoxigenin monodigitoxoside* | PDc | 2.74 | 0.01 |
| 1-(9Z-Octadecenoyl)-sn-glycero-3-phospho-(1'-myo-inositol)* | PDc | 2.16 | 0.01 |
| Grepafloxacin* | PDc | 1.49 | 0.01 |
| 4-Ethyl-2-hexylthiazole* | PDc | 1.81 | <0.01 |
| 11-Oxo-androsterone glucuronide* | PDc | 1.92 | 0.01 |
| 3-hydroxyoctanoyl carnitine* | PDc | 1.37 | 0.04 |
| Leucyl-Hydroxyproline* | PDc | 1.72 | 0.01 |
| Travoprost* | PDc | 4.11 | 0.01 |
| (+)-Fluprostenol isopropyl ester* | PDc | 4.65 | 0.01 |
| N-(1-Deoxy-1-fructosyl)valine* | PDc | 0.11 | 0.08 |
| L-2-Amino-4-methylenepentanedioic acid* | PDc | 2.18 | 0.06 |
| (E)-2-octenal* | PDc | 0.78 | 0.06 |
| Ethyl menthane carboxamide* | PDc | 0.30 | 0.08 |
| 8-Nonen-3-one* | PDc | 0.75 | 0.04 |
| 2,5-Heptadien-1-ol* | PDc | 1.37 | 0.08 |
| Isoamyl p-anisate* | PDc | 0.76 | 0.08 |
| 13-Hydroperoxy-9Z,11E-octadecadienoic acid* | PDc | 0.66 | 0.05 |
| Aspartyl-Serine* | PDc | 2.63 | 0.02 |
| Methyl hexadec-9-enoate* | PDc | 0.76 | 0.07 |
| Glu-Pro-Lys* | PDc | 1.91 | 0.09 |
| 2-Benzofurancarboxaldehyde* | PDc | 1.48 | 0.01 |
| Momilactone B* | PDc | 0.69 | 0.06 |
| Arabinosylhypoxanthine* | PDc | 2.57 | 0.06 |
| Uric acid* | PDc | 1.23 | 0.09 |
| Phenylalanyl-Asparagine* | PDc | 1.72 | 0.07 |
| Polyoxyethylene 40 monostearate* | PDc | 0.69 | 0.01 |
| Valyl-Valine* | PDc | 1.81 | 0.02 |
| Methylisocitric acid* | PDc | 1.41 | 0.03 |
| Ala Ala Arg Gly Ala* | PDc | 1.70 | 0.04 |
| Glu Phe Arg* | PDc | 0.79 | 0.08 |
| Methyl perillate* | PDc | 0.77 | 0.07 |
| Aminofurantoin* | PDc | 1.35 | 0.03 |
| Adenosine* | PDc | 1.48 | 0.02 |
| Tyr-Tyr-Arg* | PDc | 1.46 | 0.05 |
| 4-Methylnonanoic acid* | PDc | 0.82 | 0.07 |
| Thr Pro Pro Val Gln* | PDc | 1.77 | 0.03 |
| 2-O-p-Coumaroyltartronic acid* | PDc | 1.42 | 0.06 |
| Leu Ile Ser Glu* | PDc | 2.42 | 0.04 |
| 25-Acetylvulgaroside* | PDc | 2.82 | 0.01 |
| Histidine, butyl ester* | PDc | 1.82 | 0.01 |
| (S)-(-)-1,2,4-Butanetriol* | PDc | 0.75 | 0.06 |
| Gln Phe Leu Glu* | PDc | 1.58 | 0.03 |
| Ala Ala Arg Gly Ala* | PDc | 1.71 | 0.01 |
| 2-Phenyl-2-butenal* | PDc | 1.61 | 0.04 |
| Ile Glu Val His Gly* | PDc | 1.92 | 0.08 |
| Phosphoserine, butyl ester | PDc | 0.51 | 0.10 |
| Glutamyl-Hydroxyproline | PDc | 2.39 | 0.03 |
| (3,4-Dihydroxyphenyl)ethanol | PDc | 0.87 | 0.09 |
| O-Ureidohomoserine | PDc | 0.87 | 0.09 |
| 3,4-Dihydro-6-methoxy-3,7-dimethyl-1H-2-benzopyran-8-ol | PDc | 0.76 | 0.10 |
| Gln Ala | PDc | 1.56 | 0.08 |
| (R) 2,3-Dihydroxy-3-methylvalerate | PDc | 1.38 | 0.06 |
| Argininic acid | PDc | 1.25 | 0.08 |
| Hexanedioic acid, bis(2-ethylhexyl) ester | PDc | 0.74 | 0.10 |
| (R)-Lisofylline | PDc | 1.46 | 0.04 |
| 3,4,5-trihydroxy-6-[4-(5,6,7-trihydroxy-4-oxo-4H-chromen-3-yl)phenoxy]oxane-2-carboxylic acid* | PDd | 1.88 | 0.04 |
| 3-[3,4-dihydroxy-5-(3,4,5-trihydroxybenzoyloxy)benzoyloxy]-5-hydroxy-4-methoxybenzoic acid* | PDd | 1.72 | 0.01 |
| 3,3'-Thiobispropanoic acid* | PDd | 1.22 | 0.06 |
| Pro-Trp-Arg* | PDd | 0.67 | 0.07 |
| 3-[(2-Methyl-3-furanyl)thio]-4-heptanone* | PDd | 1.84 | 0.01 |
| Tetrahydro-2,5-furan-diacetic acid* | PDd | 1.24 | 0.04 |
| Octanoylglucuronide* | PDd | 0.71 | 0.02 |
| Furoparadine* | PDd | 1.28 | 0.09 |
| Diosmetin 7-O-beta-D-glucuronopyranoside* | PDd | 2.53 | 0.05 |
| S-(2,5-Dimethyl-3-furanyl) 2-furancarbothioate* | PDd | 1.88 | 0.01 |
| Gibberellin A87* | PDd | 1.47 | 0.04 |
| Hydroxyprolyl-Glutamate* | PDd | 1.91 | 0.02 |
| Cevadine* | PDd | 2.90 | 0.01 |
| Clemastine* | PDd | 2.37 | 0.08 |
| Decanenitrile, 10-(methylsulfonyl)-* | PDd | 1.44 | <0.01 |
| Cucurbitacin C* | PDd | 1.56 | 0.01 |
| Ganoderiol C* | PDd | 0.60 | 0.01 |
| Deltoside* | PDd | 2.14 | <0.01 |
| L-Cysteinylglycine disulfide* | PDd | 2.27 | <0.01 |
| Phosphonic acid, 1,2-ethanediylbis-, tetraethyl ester* | PDd | 2.43 | 0.01 |
| Apraclonidine* | PDd | 53.93 | 0.09 |
| Oryzarol* | PDd | 1.27 | 0.03 |
| 3,4,5-trihydroxy-6-[(14-hydroxy-3-methyl-1,7-dioxo-3,4,5,6,7,8,9,10-octahydro-1H-2-benzoxacyclotetradecin-16-yl)oxy]oxane-2-carboxylic acid* | PDd | 1.29 | 0.06 |
| 8-[3,7-dihydroxy-2-(3-hydroxyphenyl)-3,4-dihydro-2H-1-benzopyran-4-yl]-6-[2-(3,4-dihydroxyphenyl)-3,7-dihydroxy-3,4-dihydro-2H-1-benzopyran-4-yl]-2-(3-hydroxyphenyl)-3,4-dihydro-2H-1-benzopyran-3,5,7-triol* | PDd | 2.41 | 0.02 |
| 3beta,7alpha-Dihydroxy-5-cholestenoate* | PDd | 0.68 | <0.01 |
| 8-Methoxykynurenate* | PDd | 1.22 | 0.02 |
| Avocadene* | PDd | 0.79 | 0.02 |
| N-Octanoyl-L-homoserine lactone* | PDd | 0.42 | 0.10 |
| Goshuyic acid* | PDd | 0.70 | 0.04 |
| 9-OxoOTrE* | PDd | 0.25 | 0.05 |
| 1,5-Diaminonaphthalene* | PDd | 1.56 | 0.06 |
| 5(Z),8(Z),11(Z)-Eicosatrienoic acid methyl ester* | PDd | 0.66 | 0.04 |
| Methyl 2-octynoate* | PDd | 0.81 | 0.07 |
| N-Undecanoyl-L-homoserine lactone* | PDd | 0.61 | 0.07 |
| 9,10,13-Trihydroxystearic acid* | PDd | 0.51 | 0.04 |
| Ecklonialactone A* | PDd | 0.77 | 0.03 |
| Isobutyl 2-furanpropionate* | PDd | 1.20 | 0.05 |
| (S)-(-)-1,2,4-Butanetriol* | PDd | 0.72 | 0.07 |
| Methyl (2E,6Z)-dodecadienoate* | PDd | 0.76 | 0.04 |
| N-Decanoylglycine* | PDd | 0.58 | 0.07 |
| N2-Galacturonyl-L-lysine* | PDd | 0.81 | 0.09 |
| Menthyl pyrrolidone carboxylate* | PDd | 0.64 | 0.02 |
| 2-(4-Methyl-5-thiazolyl)ethyl propionate* | PDd | 1.28 | 0.05 |
| 4-Methoxybenzyl O-(2-sulfoglucoside)* | PDd | 1.45 | 0.02 |
| 3-Propylidene-1(3H)-isobenzofuranone* | PDd | 0.81 | 0.09 |
| p-tert-Octylphenol glycol ether* | PDd | 0.71 | 0.04 |
| LysoPC(14:0)* | PDd | 2.43 | 0.04 |
| Lucidone A* | PDd | 0.82 | 0.05 |
| 4-Acetylzearalenone* | PDd | 0.80 | 0.07 |
| .beta.-Naphthol* | PDd | 2.32 | 0.10 |
| Tyr Pro Thr Val Asn* | PDd | 2.04 | <0.01 |
| 3-carboxy-4-methyl-5-pentyl-2-furanpropanoic acid* | PDd | 0.86 | 0.07 |
| Val-Nap-OH* | PDd | 7.35 | 0.08 |
| 3-hydroxydecanoyl carnitine* | PDd | 0.77 | 0.02 |
| Prostaglandin F2.alpha. Dimethylamine* | PDd | 0.74 | 0.09 |
| Hexanal dihexyl acetal* | PDd | 0.78 | 0.02 |
| N-[(1R)-2-Hydroxy-1-methylethyl-9Z-octadecenamide* | PDd | 0.77 | 0.04 |
| His Ser Val Glu* | PDd | 1.81 | 0.03 |
| Paullinic acid* | PDd | 0.75 | 0.03 |
| 2-aminohexadecanoic acid* | PDd | 0.72 | 0.04 |
| 8-Chloroxanthine | PDd | 0.81 | 0.09 |
| Ile-Leu | PDd | 1.59 | 0.09 |
| Homovanillic acid sulfate | PDd | 0.72 | 0.10 |
| Torososide B | PDd | 1.36 | 0.08 |
| Ajoene | PDd | 0.55 | 0.10 |
| 1,4-Dithiothreitol | PDd | 1.21 | 0.10 |
| 17-trifluoromethylphenyl trinor Prostaglandin F2α ethyl amide | PDd | 1.23 | 0.10 |
| 3.52_102.0825m/z* | N/A | 3.21 | 0.02 |
| 11.48_172.1414m/z* | N/A | 0.74 | 0.10 |
| 5.71_203.0591m/z* | N/A | 0.45 | 0.04 |
| 8.58_206.0870m/z* | N/A | 0.59 | 0.03 |
| 2.74_116.0166m/z* | N/A | 0.63 | 0.02 |
| 2.45_138.0304m/z* | N/A | 1.30 | 0.02 |
| 3.61_209.1263m/z* | N/A | 2.06 | 0.08 |
| 3.12_475.2125m/z* | N/A | 0.45 | 0.03 |
| 1.73_238.0290m/z* | N/A | 1.25 | 0.09 |
| 0.62_90.0497m/z* | N/A | 1.33 | 0.04 |
| 2.87_262.9896m/z* | N/A | 1.38 | 0.04 |
| 0.69_112.0281m/z* | N/A | 0.65 | 0.06 |
| 3.52_122.0661m/z* | N/A | 2.51 | 0.01 |
| 3.71_279.6476m/z* | N/A | 2.30 | 0.06 |
| 6.25_316.1235m/z* | N/A | 2.07 | 0.01 |
| 15.97_251.1619m/z* | N/A | 0.73 | 0.06 |
| 3.14_237.1576m/z* | N/A | 1.95 | 0.02 |
| 13.66_302.2558m/z* | N/A | 3.67 | <0.01 |
| 3.12_457.7037m/z* | N/A | 0.60 | 0.09 |
| 5.59_293.6996m/z* | N/A | 1.79 | 0.05 |
| 9.26_392.1858m/z* | N/A | 0.29 | 0.03 |
| 6.32_708.4153n* | N/A | 2.94 | 0.02 |
| 1.50_216.9907m/z* | N/A | 1.55 | 0.07 |
| 3.74_387.7088m/z* | N/A | 2.57 | 0.01 |
| 2.52_176.9846m/z* | N/A | 1.12 | 0.10 |
| 4.60_237.6678m/z* | N/A | 1.77 | 0.01 |
| 4.15_380.1803m/z* | N/A | 3.04 | 0.09 |
| 4.25_344.1689m/z* | N/A | 1.70 | 0.08 |
| 7.64_443.6270m/z* | N/A | 1.79 | 0.01 |
| 12.46_357.1219m/z* | N/A | 0.72 | 0.07 |
| 2.45_277.1475m/z* | N/A | 1.26 | 0.09 |
| 8.25_382.6405m/z* | N/A | 1.55 | 0.03 |
| 3.12_471.9594m/z* | N/A | 2.13 | 0.07 |
| 1.50_125.0297m/z* | N/A | 1.54 | 0.07 |
| 1.89_127.0455m/z* | N/A | 1.66 | 0.07 |
| 6.68_351.2105m/z* | N/A | 1.78 | 0.08 |
| 7.71_556.6658m/z* | N/A | 1.65 | 0.03 |
| 1.89_189.5573m/z* | N/A | 2.25 | 0.03 |
| 3.92_237.6370m/z* | N/A | 3.07 | 0.02 |
| 4.70_421.1030m/z* | N/A | 1.64 | 0.08 |
| 1.71_195.0629m/z* | N/A | 1.23 | 0.03 |
| 4.72_128.0424m/z* | N/A | 1.23 | 0.09 |
| 8.45_293.1429m/z* | N/A | 2.24 | 0.02 |
| 3.12_471.7087m/z* | N/A | 2.23 | 0.08 |
| 15.59_282.2169n* | N/A | 0.78 | 0.09 |
| 11.99_467.1453m/z* | N/A | 2.67 | 0.05 |
| 8.71_254.9714m/z* | N/A | 0.78 | 0.04 |
| 5.00_457.1579m/z* | N/A | 1.49 | 0.02 |
| 6.02_299.1313m/z* | N/A | 1.97 | 0.09 |
| 2.97_435.7069m/z* | N/A | 2.06 | 0.01 |
| 15.09_341.1812n* | N/A | 0.79 | 0.08 |
| 12.89_551.2824m/z* | N/A | 2.33 | 0.01 |
| 11.64_269.1317m/z* | N/A | 2.46 | 0.06 |
| 6.30_477.7721m/z* | N/A | 2.92 | 0.01 |
| 6.45_288.1320n* | N/A | 1.88 | 0.08 |
| 3.79_344.1927m/z* | N/A | 3.18 | <0.01 |
| 4.97_255.0869m/z* | N/A | 1.65 | 0.04 |
| 4.62_365.6900m/z* | N/A | 2.21 | 0.03 |
| 1.03_348.0696n* | N/A | 1.38 | 0.01 |
| 4.30_181.0569m/z* | N/A | 2.13 | 0.05 |
| 3.94_399.1697m/z* | N/A | 1.72 | 0.05 |
| 7.54_311.2020m/z* | N/A | 1.90 | 0.01 |
| 5.38_725.8083m/z* | N/A | 1.90 | 0.04 |
| 2.77_294.6585m/z* | N/A | 2.20 | 0.02 |
| 5.92_243.1339m/z* | N/A | 2.78 | <0.01 |
| 5.20_400.7348m/z* | N/A | 3.02 | 0.01 |
| 8.10_444.2817m/z* | N/A | 1.88 | 0.03 |
| 6.86_581.3408m/z* | N/A | 2.04 | 0.02 |
| 4.22_416.7115m/z* | N/A | 1.89 | 0.06 |
| 4.35_398.6848m/z* | N/A | 1.87 | 0.03 |
| 4.15_234.0795m/z* | N/A | 1.63 | 0.06 |
| 8.94_791.9018m/z* | N/A | 1.84 | 0.03 |
| 1.50_216.0311n* | N/A | 1.70 | 0.10 |
| 1.03_232.0471m/z* | N/A | 1.22 | 0.10 |
| 6.55_452.9051m/z* | N/A | 2.11 | 0.01 |
| 8.89_704.7109m/z* | N/A | 1.52 | 0.03 |
| 7.64_400.6531m/z* | N/A | 1.69 | 0.01 |
| 15.23_520.5621m/z* | N/A | 1.47 | 0.02 |
| 4.30_422.8618m/z* | N/A | 1.78 | 0.09 |
| 1.14_290.6320m/z* | N/A | 2.51 | 0.03 |
| 7.24_676.8623m/z* | N/A | 2.04 | 0.07 |
| 4.62_378.6453m/z* | N/A | 1.92 | 0.03 |
| 6.17_119.0730n* | N/A | 1.28 | 0.01 |
| 6.83_588.8002m/z* | N/A | 1.66 | 0.10 |
| 15.54_601.3510m/z* | N/A | 0.67 | 0.02 |
| 3.09_197.1285m/z* | N/A | 1.31 | 0.09 |
| 10.49_330.0944m/z* | N/A | 3.00 | <0.01 |
| 0.65_162.1254m/z* | N/A | 0.78 | 0.05 |
| 4.90_279.6861m/z* | N/A | 1.41 | 0.03 |
| 7.24_676.6116m/z* | N/A | 2.37 | 0.04 |
| 8.63_600.1835m/z* | N/A | 2.27 | <0.01 |
| 10.79_367.1872n* | N/A | 1.19 | 0.07 |
| 10.36_1198.6101n* | N/A | 1.80 | 0.09 |
| 1.01_169.5948m/z* | N/A | 1.84 | 0.02 |
| 7.66_742.3858n* | N/A | 1.74 | 0.05 |
| 0.51_231.9919n* | N/A | 1.48 | 0.07 |
| 14.74_634.9170m/z* | N/A | 1.29 | 0.04 |
| 9.93_825.6692m/z* | N/A | 1.83 | 0.02 |
| 8.07_553.2471m/z* | N/A | 4.46 | 0.07 |
| 12.33_850.6566m/z* | N/A | 1.53 | 0.10 |
| 2.74_342.1427m/z* | N/A | 1.65 | 0.03 |
| 14.59_557.3296m/z* | N/A | 2.61 | 0.01 |
| 3.22_463.9687m/z* | N/A | 1.76 | 0.04 |
| 1.89_217.9874m/z* | N/A | 1.81 | 0.06 |
| 5.18_808.3885n* | N/A | 1.86 | 0.03 |
| 6.37_287.6127m/z* | N/A | 1.66 | 0.03 |
| 8.38_803.8524m/z* | N/A | 1.40 | 0.06 |
| 6.17_344.6342m/z* | N/A | 1.82 | 0.01 |
| 8.71_1016.7787m/z* | N/A | 1.54 | 0.08 |
| 8.84_824.4202m/z* | N/A | 1.81 | 0.04 |
| 8.61_1241.4206n* | N/A | 1.52 | 0.03 |
| 14.10_507.2291n* | N/A | 0.63 | 0.03 |
| 1.89_168.0174n* | N/A | 1.39 | 0.03 |
| 6.17_417.2344m/z* | N/A | 2.46 | 0.06 |
| 5.28_556.2997m/z* | N/A | 2.10 | 0.08 |
| 0.65_329.9232m/z* | N/A | 1.44 | 0.03 |
| 8.48_548.0706m/z* | N/A | 2.06 | 0.01 |
| 9.41_786.9030m/z* | N/A | 2.38 | 0.02 |
| 6.17_455.2510m/z* | N/A | 1.80 | 0.02 |
| 3.22_463.7179m/z* | N/A | 1.78 | 0.04 |
| 8.17_512.6467m/z* | N/A | 1.47 | <0.01 |
| 0.68_401.1142m/z* | N/A | 1.33 | 0.05 |
| 4.62_718.3365m/z* | N/A | 1.86 | 0.05 |
| 8.48_487.6750m/z* | N/A | 1.99 | <0.01 |
| 6.93_582.2740n* | N/A | 4.45 | 0.02 |
| 9.36_1220.5191n* | N/A | 1.26 | 0.09 |
| 8.48_547.8199m/z* | N/A | 2.04 | 0.01 |
| 10.39_739.3375m/z* | N/A | 1.60 | 0.03 |
| 7.66_418.7474m/z* | N/A | 1.76 | <0.01 |
| 7.51_1567.6216n* | N/A | 1.23 | 0.06 |
| 6.17_479.7012m/z* | N/A | 1.84 | 0.01 |
| 4.30_398.0763n* | N/A | 3.61 | 0.02 |
| 15.48_376.3182m/z* | N/A | 0.75 | 0.02 |
| 15.95_368.2906n* | N/A | 0.86 | 0.04 |
| 0.69_331.0898n* | N/A | 1.46 | 0.01 |
| 9.51_820.1646m/z* | N/A | 2.53 | 0.01 |
| 6.68_350.2071m/z* | N/A | 1.61 | 0.05 |
| 8.89_703.7078m/z* | N/A | 2.44 | 0.07 |
| 9.41_786.6521m/z* | N/A | 2.50 | 0.01 |
| 8.53_771.0547m/z* | N/A | 1.99 | 0.06 |
| 0.78_239.1367n* | N/A | 1.53 | 0.02 |
| 0.62_279.9465m/z* | N/A | 1.30 | 0.04 |
| 0.68_279.0093m/z* | N/A | 1.31 | 0.04 |
| 0.56_728.7960n* | N/A | 1.62 | 0.05 |
| 5.28_296.6236m/z* | N/A | 2.00 | 0.08 |
| 12.87_551.3066n* | N/A | 2.73 | 0.01 |
| 6.88_334.6897m/z* | N/A | 1.69 | 0.04 |
| 1.10_150.0470m/z* | N/A | 1.29 | 0.03 |
| 0.56_951.7837m/z* | N/A | 0.73 | 0.09 |
| 7.29_683.0036m/z* | N/A | 1.75 | 0.01 |
| 15.46_460.2820m/z* | N/A | 1.29 | 0.07 |
| 11.53_369.2028n* | N/A | 1.97 | 0.06 |
| 8.27_343.1889m/z* | N/A | 1.61 | 0.03 |
| 15.37_528.3036m/z* | N/A | 1.19 | 0.03 |
| 0.75_362.9304m/z* | N/A | 0.75 | 0.03 |
| 9.70_909.1336m/z* | N/A | 6.30 | 0.08 |
| 6.45_533.9498m/z* | N/A | 1.69 | 0.01 |
| 7.08_522.2842m/z* | N/A | 1.71 | 0.08 |
| 15.48_549.3277n* | N/A | 1.17 | 0.01 |
| 1.18_409.0858n* | N/A | 1.37 | 0.10 |
| 0.51_217.9763n* | N/A | 1.31 | 0.08 |
| 10.21_1285.6423n* | N/A | 1.83 | 0.04 |
| 1.16_1994.3563n* | N/A | 2.32 | 0.03 |
| 10.72_600.9611m/z* | N/A | 1.43 | 0.08 |
| 8.33_1205.5224n* | N/A | 1.72 | 0.01 |
| 8.61_624.2230m/z* | N/A | 1.40 | 0.03 |
| 8.74_816.4079m/z* | N/A | 2.28 | 0.01 |
| 6.57_330.6766m/z* | N/A | 3.28 | 0.02 |
| 9.64_880.7568m/z* | N/A | 1.38 | 0.09 |
| 13.68_977.3786m/z* | N/A | 1.25 | 0.09 |
| 1.16_1680.2814n* | N/A | 1.93 | 0.01 |
| 9.09_1022.5013m/z* | N/A | 2.96 | 0.01 |
| 11.05_385.1824n* | N/A | 0.88 | 0.07 |
| 12.74_846.9009m/z* | N/A | 1.43 | 0.06 |
| 8.30_1033.2206m/z* | N/A | 1.68 | 0.05 |
| 7.71_1047.5955n* | N/A | 2.78 | 0.02 |
| 0.75_302.9747m/z* | N/A | 0.76 | 0.03 |
| 6.17_650.3145m/z* | N/A | 1.83 | 0.01 |
| 7.64_401.6503n* | N/A | 1.67 | <0.01 |
| 8.71_1011.9821m/z* | N/A | 1.44 | 0.07 |
| 1.16_692.0937m/z* | N/A | 1.31 | 0.04 |
| 0.75_222.0270n* | N/A | 0.76 | 0.04 |
| 8.53_2282.1461n* | N/A | 2.24 | 0.02 |
| 8.33_1032.8202m/z* | N/A | 1.76 | 0.04 |
| 9.90_932.1363m/z* | N/A | 3.06 | 0.02 |
| 8.35_650.7129m/z* | N/A | 2.83 | 0.01 |
| 9.26_606.5994m/z* | N/A | 1.37 | 0.10 |
| 8.50_786.7257m/z* | N/A | 1.85 | 0.04 |
| 6.83_752.4565n* | N/A | 1.68 | 0.04 |
| 6.12_269.6547m/z* | N/A | 1.61 | 0.04 |
| 8.25_390.6291m/z* | N/A | 1.22 | 0.06 |
| 15.66_770.9636m/z* | N/A | 1.15 | 0.03 |
| 5.00_272.0207n* | N/A | 26.29 | 0.02 |
| 2.74_622.3419n* | N/A | 1.59 | 0.04 |
| 7.47_401.7321m/z* | N/A | 1.75 | 0.02 |
| 4.35_735.4261n* | N/A | 1.62 | 0.03 |
| 8.61_1247.4375n* | N/A | 1.39 | 0.04 |
| 15.66_770.4609n* | N/A | 1.18 | 0.01 |
| 15.66_948.6169n* | N/A | 1.49 | 0.01 |
| 8.17_1008.3142n* | N/A | 1.35 | 0.01 |
| 7.01_866.4999n* | N/A | 1.54 | 0.05 |
| 9.29_1361.5731n* | N/A | 1.22 | 0.05 |
| 10.03_2282.2128n* | N/A | 1.24 | 0.05 |
| 3.84_527.2125n* | N/A | 1.45 | 0.06 |
| 15.88_404.3160n* | N/A | 0.82 | 0.06 |
| 7.44_1240.6071n* | N/A | 1.57 | 0.03 |
| 8.58_1379.5840n* | N/A | 1.22 | 0.05 |
| 7.16_1054.5438n* | N/A | 1.60 | 0.03 |
| 8.74_2386.2736n* | N/A | 1.96 | 0.01 |
| 7.49_1349.6207n* | N/A | 1.52 | 0.03 |
| 15.68_454.2916n* | N/A | 1.26 | 0.01 |
| 9.29_1395.5576n* | N/A | 1.40 | 0.02 |
| 2.20_193.9708m/z | N/A | 0.78 | 0.09 |
| 0.81_176.1161m/z | N/A | 1.37 | 0.10 |
| 2.25_115.0584m/z | N/A | 1.25 | 0.07 |
| 5.46_371.1924m/z | N/A | 1.66 | 0.07 |
| 4.55_421.1749m/z | N/A | 1.84 | 0.05 |
| 4.87_512.4860m/z | N/A | 2.05 | 0.08 |
| 4.60_358.1205m/z | N/A | 1.83 | 0.10 |
| 9.01_674.8333m/z | N/A | 2.30 | 0.09 |
| 2.69_220.6163m/z | N/A | 1.38 | 0.10 |
| 3.22_366.7035m/z | N/A | 1.57 | 0.09 |
| 4.87_512.2353m/z | N/A | 2.40 | 0.10 |
| 3.92_364.8731m/z | N/A | 1.86 | 0.08 |
| 5.59_889.4338n | N/A | 1.98 | 0.07 |
| 1.43_247.1481m/z | N/A | 1.64 | 0.10 |
| 2.47_488.2465m/z | N/A | 1.64 | 0.10 |
| 3.09_278.1478m/z | N/A | 1.77 | 0.08 |
| 1.85_86.1014m/z | N/A | 1.15 | 0.10 |
| 10.61_675.6580m/z | N/A | 1.58 | 0.09 |
| 1.37_158.0126m/z | N/A | 1.25 | 0.06 |
| 8.38_571.2848m/z | N/A | 4.84 | 0.09 |
| 6.40_1013.0246m/z | N/A | 1.50 | 0.09 |
| 6.22_671.6687m/z | N/A | 2.74 | 0.06 |
| 0.61_343.8616m/z | N/A | 0.82 | 0.09 |
| 0.62_337.9051m/z | N/A | 1.26 | 0.08 |
| 1.62_181.9269m/z | N/A | 1.26 | 0.10 |
| 8.76_663.7941m/z | N/A | 1.61 | 0.06 |
| 7.01_464.2218m/z | N/A | 1.46 | 0.08 |
| 5.76_824.4133m/z | N/A | 2.74 | 0.06 |
| 0.56_866.8245m/z | N/A | 0.73 | 0.08 |
| 0.75_360.9333m/z | N/A | 0.79 | 0.07 |
| 0.54_816.1754m/z | N/A | 0.75 | 0.08 |
| 5.48_1141.6240n | N/A | 1.45 | 0.08 |
| 8.30_860.5171m/z | N/A | 1.55 | 0.10 |
| 10.26_892.3909m/z | N/A | 1.49 | 0.09 |
| 7.26_360.6998m/z | N/A | 1.44 | 0.05 |
| 8.50_590.5471m/z | N/A | 1.51 | 0.09 |
| 2.01_132.0066m/z | N/A | 1.16 | 0.09 |
| 8.53_552.1966m/z | N/A | 1.24 | 0.09 |
| 5.59_873.4693n | N/A | 1.73 | 0.05 |
| 1.62_182.0965m/z | N/A | 1.22 | 0.07 |
| 15.46_445.2938n | N/A | 0.42 | 0.09 |
| 10.72_798.9348m/z | N/A | 1.64 | 0.09 |
| 8.33_1032.6197m/z | N/A | 1.56 | 0.09 |
| 9.98_944.2340m/z | N/A | 1.57 | 0.09 |
| 10.72_958.7213m/z | N/A | 1.49 | 0.10 |

^a^Cut-off criterion for the associated peaks/metabolites is p<0.1 without adjusting for covariates; the unidentified or unannotated peaks are listed with retention (RT) and exact mass (m/z or neutral mass). *Metabolites that predict PE after adjusting for covariates (p<0.1). ^b^ Ontology levels: OL1, highly confident identification based on matching with In-house physical standard library (IPSL) via retention time (RT, with RT error≤|0.5|), exact mass (MS, with mass error<5ppm), and tandem mass similarity (MS/MS, with similarity ≥30); OL2a, confident identification based on matching with IPSL via MS and RT; OL2b, annotation for the isomer or derivatives of the compound listed but not the compound itself, based on matching with IPSL via MS and MS/MS; PDa, annotation based on matching with public database via MS and experimental MS/MS (could be the listed compound, or the isomer or derivatives of the listed compound); PDb, annotation based on matching with public database via MS and predict MS/MS; PDc, annotation for the listed compound based on matching with public database via MS and isotopic similarity or adducts; PDd annotation for listed compound based on matching with public database via MS; N/A, peaks was not identified or annotated. ^c^FC, fold change, the ratio of intensity between the PE subjects vs control, based on the mean, indicates the direction and magnitude of change: FC>1.0 indicates increase compared to control and FC<1.0 indicates decrease compared to control. ^d^ p-value determined by logistic modeling.

**Table S5. UPLC-HR-MS determined metabolites/peaks that are associated with preterm birth (PTB) (univariate logistic regression, p<0.1)**

| **Metabolite/peak**  **(246 peaks, 189 with annotation or identification)** ^a^ | **Ontology level ^b^** | **FC^c^** | ***p*-value^d^** |
| --- | --- | --- | --- |
| Corticosterone | OL1 | 1.44 | 0.03 |
| Bisphenol S | OL1 | 0.91 | 0.07 |
| Glutarate | OL1 | 0.90 | 0.03 |
| 3-hydroxybutanoate* | OL2a | 0.84 | 0.01 |
| Butyrylcarnitine* | OL2a | 1.18 | 0.08 |
| S-carboxymethylcysteine* | OL2a | 1.24 | 0.07 |
| Cortisol* | OL2a | 1.42 | 0.05 |
| Homocysteine thiolactone* | OL2a | 0.73 | 0.10 |
| 3,4-dihydroxyphenylacetate | OL2a | 0.88 | 0.07 |
| 3-hydroxybutanoate | OL2a | 0.86 | 0.05 |
| 2,6-dimethoxyphenol* | OL2b | 0.64 | 0.05 |
| Pyridoxal* | OL2b | 0.87 | 0.02 |
| Mevalonate | OL2b | 0.94 | 0.09 |
| Glutarate | OL2b | 0.93 | 0.08 |
| Met-Ala* | PDa | 0.71 | 0.03 |
| Ser Pro Phe* | PDa | 0.64 | 0.04 |
| Trans,trans-Muconic acid | PDa | 0.91 | 0.07 |
| 17-hydroxyprogesterone | PDa | 1.00 | 0.04 |
| Asparaginyl-Lysine* | PDb | 0.57 | 0.03 |
| Biocytin* | PDb | 0.73 | 0.09 |
| Feruperine* | PDb | 1.50 | 0.10 |
| gamma-Glutamylisoleucine* | PDb | 0.62 | 0.04 |
| Pyrrolidine* | PDb | 0.91 | 0.04 |
| L-Pyridosine | PDb | 0.71 | 0.08 |
| Cortolone-3-glucuronide | PDb | 1.27 | 0.03 |
| Cortolone-3-glucuronide | PDb | 1.18 | 0.09 |
| Jasmonic acid* | PDc | 0.47 | 0.08 |
| Nitroacetic acid ethyl ester* | PDc | 0.83 | 0.03 |
| (1xi,3S)-1,2,3,4-Tetrahydro-1-methyl-beta-carboline-1,3-dicarboxylic acid* | PDc | 0.41 | 0.02 |
| Ethyl 5-hexenoate* | PDc | 0.77 | 0.03 |
| 5-Phosphoribosylamine* | PDc | 0.53 | 0.05 |
| (Â±)-Anisoxide* | PDc | 1.24 | 0.04 |
| 2-Methylbutyrylglycine* | PDc | 1.93 | 0.05 |
| Piperanine* | PDc | 1.63 | 0.07 |
| 4-hydroxy-3-nitrophenylacetate* | PDc | 0.85 | 0.02 |
| Nitroguanidine* | PDc | 0.80 | 0.03 |
| 5-[2H-Pyrrol-4-(3H)-ylidenemethyl]-2-furanmethanol* | PDc | 0.83 | 0.07 |
| Homoarecoline* | PDc | 0.80 | 0.02 |
| Di(propylene glycol) propyl ether* | PDc | 0.81 | 0.03 |
| S-(2-Aminoethyl)isothiourea* | PDc | 0.77 | 0.02 |
| 4-Hydroxycyclohexylcarboxylic acid* | PDc | 0.89 | 0.02 |
| Aciclovir* | PDc | 1.23 | 0.08 |
| Testosterone enanthate* | PDc | 1.36 | 0.04 |
| (3,4-Dihydroxyphenyl)ethanol* | PDc | 0.89 | 0.02 |
| Isopentyl pyrophosphate* | PDc | 0.84 | 0.03 |
| N,N-Dimethyl-L-valine* | PDc | 0.77 | 0.01 |
| Glycylprolylhydroxyproline* | PDc | 0.65 | 0.01 |
| Hydroxy-lacosamide* | PDc | 1.53 | 0.08 |
| Cysteinyl-Proline* | PDc | 0.87 | 0.06 |
| Val-Met* | PDc | 1.96 | 0.03 |
| 3-Sulfopyruvic acid* | PDc | 0.89 | 0.02 |
| 6-[1-(6,7-dimethoxy-2H-1,3-benzodioxol-5-yl)-3-oxopropoxy]-3,4,5-trihydroxyoxane-2-carboxylic acid* | PDc | 0.73 | 0.06 |
| 3-Hexenedioic acid* | PDc | 0.89 | 0.05 |
| Arecoline* | PDc | 0.77 | 0.02 |
| 1-Hexanol, 6-amino-* | PDc | 0.86 | 0.03 |
| 3-hydroxyoctanoyl carnitine* | PDc | 1.22 | 0.05 |
| Dacarbazine* | PDc | 0.85 | 0.02 |
| Pregabalin | PDc | 0.82 | 0.09 |
| Isoeugenitin | PDc | 0.57 | 0.06 |
| gamma-Glutaminyl-4-hydroxybenzene | PDc | 0.77 | 0.04 |
| Methionyl-Phenylalanine | PDc | 0.45 | 0.08 |
| 2-methyl-1,3-thiazolidine-2-carboxamide | PDc | 0.92 | 0.08 |
| Acrylamide | PDc | 0.73 | 0.07 |
| 1-(9Z-Octadecenoyl)-sn-glycero-3-phospho-(1'-myo-inositol) | PDc | 1.60 | 0.03 |
| 7-Keto-8-aminopelargonic acid | PDc | 0.86 | 0.08 |
| 4-Hydroxycyclohexylcarboxylic acid | PDc | 0.94 | 0.09 |
| 2-Aminoheptanoate | PDc | 0.94 | 0.09 |
| 3-Hexenedioic acid | PDc | 0.92 | 0.09 |
| N-Ethylmaleimide | PDc | 0.88 | 0.06 |
| 2-(sulfooxy)pentanoic acid | PDc | 0.75 | 0.10 |
| Nithiamide | PDc | 0.81 | 0.05 |
| S-2,5-Dimethyl-3-furanyl 3-methylbutanethioate* | PDd | 0.64 | 0.01 |
| Indole-2-carboxylic acid* | PDd | 0.85 | 0.01 |
| {3-[(2E)-3-phenylprop-2-enoyl]phenyl}oxidanesulfonic acid* | PDd | 0.76 | 0.01 |
| 4,7,8-trihydroxy-2H-chromen-2-one* | PDd | 0.85 | 0.01 |
| 4-Methoxycoumarin* | PDd | 0.65 | 0.05 |
| 3,3'-Thiobispropanoic acid* | PDd | 0.88 | 0.03 |
| 3-nonenoylglycine* | PDd | 1.17 | 0.10 |
| Ile-Leu* | PDd | 1.49 | 0.04 |
| Tetrahydro-2,5-furan-diacetic acid* | PDd | 0.84 | 0.03 |
| Phe-Arg-Arg* | PDd | 0.44 | 0.01 |
| Methyl 2E,4Z-hexadecadienoate* | PDd | 0.82 | 0.07 |
| cis-4-Octenedioic acid* | PDd | 0.92 | 0.07 |
| 4-Oxo-1-(3-pyridyl)-1-butanone* | PDd | 0.83 | 0.04 |
| 2,6-Dimethyl-naphtalene* | PDd | 0.73 | 0.02 |
| Lys-Pro-Arg* | PDd | 1.30 | 0.02 |
| Torososide B* | PDd | 1.30 | 0.02 |
| (±)- 2-Butylthiazolidine* | PDd | 0.89 | 0.05 |
| (±)- 2-Propylthiazolidine* | PDd | 0.83 | 0.01 |
| Dibenzylamine* | PDd | 0.90 | 0.05 |
| 27-Norcholestanehexol* | PDd | 1.19 | 0.05 |
| Muzanzagenin* | PDd | 1.27 | 0.06 |
| 1-Propenyl 1-(1-propenylthio)propyl disulfide* | PDd | 0.82 | 0.01 |
| 2'-Hydroxyacetanilide* | PDd | 0.87 | 0.01 |
| 2,4,5,7alpha-Tetrahydro-1,4,4,7a-tetramethyl-1H-inden-2-ol* | PDd | 0.88 | 0.01 |
| Phosphonic acid, 1,2-ethanediylbis-, tetraethyl ester* | PDd | 1.87 | 0.01 |
| 3-Methylbutyl glucosinolate* | PDd | 0.71 | 0.08 |
| Talinolol* | PDd | 1.15 | 0.02 |
| Celiprolol* | PDd | 1.17 | 0.02 |
| Chalcone* | PDd | 0.91 | 0.04 |
| Leu-Pro-Lys* | PDd | 1.17 | 0.02 |
| Lyciumoside II* | PDd | 1.49 | 0.02 |
| 8-Methoxykynurenate* | PDd | 1.12 | 0.04 |
| 2-Heptylfuran | PDd | 0.73 | 0.05 |
| 1-Propenyl 1-(propylsulfinyl)propyl disulfide | PDd | 0.52 | 0.08 |
| Cevadine | PDd | 1.67 | 0.09 |
| 4-Nitrobenzylamine | PDd | 0.87 | 0.06 |
| Ethyl octynecarboxylate | PDd | 0.93 | 0.09 |
| Bergaptol | PDd | 0.87 | 0.10 |
| Homovanillic acid sulfate | PDd | 0.82 | 0.07 |
| 2-amino-4-({1-[(carboxymethyl)-C-hydroxycarbonimidoyl]-2-{[2-hydroxy-3-(4-hydroxyphenyl)-1-(5-methoxy-2,2-dimethyl-2H-chromen-6-yl)-3-oxopropyl]sulfanyl}ethyl}-C-hydroxycarbonimidoyl)butanoic acid | PDd | 1.27 | 0.07 |
| 2-Amino-a-carboline | PDd | 0.89 | 0.07 |
| 5-(2-Methylpropyl)tetrahydro-2-oxo-3-furancarboxylic acid | PDd | 0.93 | 0.05 |
| Methylimidazole acetaldehyde | PDd | 0.91 | 0.07 |
| 8-[3,7-dihydroxy-2-(3-hydroxyphenyl)-3,4-dihydro-2H-1-benzopyran-4-yl]-6-[2-(3,4-dihydroxyphenyl)-3,7-dihydroxy-3,4-dihydro-2H-1-benzopyran-4-yl]-2-(3-hydroxyphenyl)-3,4-dihydro-2H-1-benzopyran-3,5,7-triol | PDd | 1.59 | 0.06 |
| 3,4,5-trihydroxy-6-[(14-hydroxy-3-methyl-1,7-dioxo-3,4,5,6,7,8,9,10-octahydro-1H-2-benzoxacyclotetradecin-16-yl)oxy]oxane-2-carboxylic acid | PDd | 1.16 | 0.08 |
| 0.51_84.9549m/z* | N/A | 0.87 | 0.08 |
| 7.21_131.0688m/z* | N/A | 0.76 | 0.09 |
| 1.78_175.1505m/z* | N/A | 0.66 | 0.08 |
| 2.87_139.9948m/z* | N/A | 0.87 | 0.06 |
| 5.46_199.5255m/z* | N/A | 1.45 | 0.06 |
| 1.25_102.9640m/z* | N/A | 0.87 | 0.02 |
| 5.15_327.6946m/z* | N/A | 0.66 | 0.06 |
| 3.34_134.9673m/z* | N/A | 0.83 | 0.01 |
| 0.51_102.9639m/z* | N/A | 0.68 | 0.02 |
| 2.57_152.0646m/z* | N/A | 0.82 | <0.01 |
| 0.83_121.0077m/z* | N/A | 0.88 | 0.04 |
| 7.03_532.6234m/z* | N/A | 0.46 | 0.10 |
| 7.29_507.2374m/z* | N/A | 0.46 | 0.04 |
| 3.56_120.9891m/z* | N/A | 0.87 | 0.01 |
| 14.87_277.6686m/z* | N/A | 1.24 | 0.08 |
| 8.91_763.6418m/z* | N/A | 0.58 | 0.10 |
| 0.62_104.1265m/z* | N/A | 0.89 | 0.05 |
| 2.74_205.0244m/z* | N/A | 0.87 | 0.03 |
| 0.62_190.1379m/z* | N/A | 1.36 | 0.06 |
| 15.97_291.6842m/z* | N/A | 1.18 | 0.09 |
| 8.91_763.3911m/z* | N/A | 0.60 | 0.10 |
| 7.51_591.6469m/z* | N/A | 0.75 | 0.05 |
| 1.75_142.0608n* | N/A | 0.83 | 0.04 |
| 3.34_991.4879n* | N/A | 0.57 | 0.07 |
| 10.39_976.0815m/z* | N/A | 0.78 | 0.08 |
| 7.79_216.0311n* | N/A | 0.70 | 0.05 |
| 14.72_499.3242m/z* | N/A | 5.18 | 0.08 |
| 4.44_117.0663n* | N/A | 0.91 | 0.04 |
| 7.21_535.3096m/z* | N/A | 0.68 | 0.08 |
| 11.74_455.3214m/z* | N/A | 1.27 | 0.01 |
| 14.07_533.2666n* | N/A | 1.41 | 0.02 |
| 13.03_513.3635m/z* | N/A | 1.18 | 0.06 |
| 6.19_219.0540m/z* | N/A | 0.90 | 0.03 |
| 14.20_575.3697m/z* | N/A | 1.25 | 0.01 |
| 8.56_861.0300m/z* | N/A | 0.69 | 0.05 |
| 8.48_547.8199m/z* | N/A | 1.55 | 0.02 |
| 1.16_459.0249m/z* | N/A | 0.86 | 0.02 |
| 0.61_403.8173m/z* | N/A | 0.87 | 0.06 |
| 6.40_431.9056m/z* | N/A | 0.69 | 0.09 |
| 10.39_975.6808m/z* | N/A | 0.83 | 0.07 |
| 10.36_1827.0030n* | N/A | 1.69 | 0.03 |
| 14.10_507.2291n* | N/A | 0.77 | 0.03 |
| 14.20_1148.7870n* | N/A | 1.20 | 0.04 |
| 13.61_571.4049m/z* | N/A | 1.15 | 0.01 |
| 10.69_955.7174m/z* | N/A | 0.50 | 0.04 |
| 10.49_471.3162m/z* | N/A | 1.18 | 0.01 |
| 0.56_560.8717m/z* | N/A | 0.71 | 0.08 |
| 10.69_956.1189m/z* | N/A | 0.55 | 0.05 |
| 0.56_226.9463m/z* | N/A | 0.87 | 0.08 |
| 4.32_132.0440n* | N/A | 0.78 | 0.05 |
| 10.69_955.9183m/z* | N/A | 0.56 | 0.06 |
| 0.56_866.8245m/z* | N/A | 0.82 | 0.08 |
| 8.91_795.3987m/z* | N/A | 0.70 | 0.04 |
| 8.91_794.9990m/z* | N/A | 0.53 | 0.03 |
| 13.58_543.3973n* | N/A | 1.14 | 0.02 |
| 14.10_1015.5208m/z* | N/A | 0.77 | 0.02 |
| 9.62_924.8135m/z* | N/A | 0.56 | 0.06 |
| 8.74_795.2020m/z* | N/A | 0.53 | 0.05 |
| 0.56_1017.2949m/z* | N/A | 0.86 | 0.08 |
| 14.12_1015.9340m/z* | N/A | 0.80 | 0.02 |
| 15.86_669.9560m/z* | N/A | 1.13 | 0.07 |
| 0.56_939.7901n* | N/A | 0.84 | 0.02 |
| 9.14_791.5979m/z* | N/A | 0.73 | 0.06 |
| 0.56_1016.7937m/z* | N/A | 0.87 | 0.02 |
| 15.66_770.9636m/z* | N/A | 1.10 | 0.02 |
| 0.54_3097.3756n* | N/A | 0.89 | 0.08 |
| 15.66_770.4609n* | N/A | 1.13 | <0.01 |
| 15.66_522.7946m/z* | N/A | 1.09 | 0.06 |
| 9.14_791.9991m/z* | N/A | 0.75 | 0.07 |
| 8.68_901.3495n* | N/A | 1.16 | 0.05 |
| 7.51_1128.3677n* | N/A | 0.81 | 0.04 |
| 15.88_404.3160n* | N/A | 0.85 | 0.02 |
| 0.59_615.6859n* | N/A | 0.89 | 0.09 |
| 6.93_582.2740n | N/A | 0.05 | 0.10 |
| 3.52_102.0825m/z | N/A | 2.09 | 0.06 |
| 12.28_222.0947m/z | N/A | 1.37 | 0.08 |
| 5.71_161.0410m/z | N/A | 0.85 | 0.03 |
| 0.88_150.9002m/z | N/A | 0.86 | 0.05 |
| 1.62_117.0573m/z | N/A | 0.88 | 0.09 |
| 6.37_646.2939m/z | N/A | 0.63 | 0.12 |
| 14.02_484.2751m/z | N/A | 0.41 | 0.06 |
| 7.87_279.9912m/z | N/A | 0.87 | 0.05 |
| 5.81_235.9997n | N/A | 0.56 | 0.08 |
| 15.64_124.2082m/z | N/A | 1.14 | 0.04 |
| 0.90_98.9979n | N/A | 0.88 | 0.07 |
| 6.30_477.7721m/z | N/A | 1.80 | 0.05 |
| 8.20_837.6319m/z | N/A | 0.70 | 0.10 |
| 13.58_406.1809n | N/A | 0.19 | 0.07 |
| 9.26_392.1858m/z | N/A | 0.59 | 0.05 |
| 15.77_468.3763m/z | N/A | 1.29 | 0.06 |
| 0.53_441.0318m/z | N/A | 1.21 | 0.06 |
| 11.36_479.2268n | N/A | 0.61 | 0.05 |
| 8.63_600.1835m/z | N/A | 1.32 | 0.09 |
| 14.74_589.3857m/z | N/A | 1.34 | 0.06 |
| 8.20_833.3900m/z | N/A | 0.76 | 0.09 |
| 7.01_453.7377m/z | N/A | 0.71 | 0.08 |
| 15.13_274.6633m/z | N/A | 1.22 | 0.05 |
| 14.59_557.3296m/z | N/A | 1.47 | 0.06 |
| 8.63_547.2084m/z | N/A | 0.80 | 0.08 |
| 0.78_210.8558m/z | N/A | 0.92 | 0.09 |
| 12.33_850.6566m/z | N/A | 1.39 | 0.09 |
| 0.88_208.8587m/z | N/A | 0.92 | 0.09 |
| 8.48_548.0706m/z | N/A | 1.48 | 0.03 |
| 7.64_2049.0704n | N/A | 1.34 | 0.08 |
| 3.41_165.5513m/z | N/A | 0.87 | 0.06 |
| 0.61_131.8962n | N/A | 0.79 | 0.03 |
| 14.38_514.2751n | N/A | 0.68 | 0.06 |
| 0.64_336.9406m/z | N/A | 0.91 | 0.06 |
| 9.57_839.4082m/z | N/A | 1.32 | 0.09 |
| 9.14_799.7892m/z | N/A | 0.73 | 0.09 |
| 9.09_1022.5013m/z | N/A | 1.75 | 0.05 |
| 12.20_999.8890m/z | N/A | 1.61 | 0.07 |
| 8.74_816.4079m/z | N/A | 1.44 | 0.08 |
| 12.53_809.9179m/z | N/A | 1.44 | 0.05 |
| 1.16_1994.3563n | N/A | 1.80 | 0.06 |
| 3.69_201.0314n | N/A | 0.76 | 0.09 |
| 13.16_805.6613m/z | N/A | 1.42 | 0.06 |
| 15.44_311.6327m/z | N/A | 0.87 | 0.07 |
| 4.44_209.1030n | N/A | 0.88 | 0.09 |
| 14.10_1016.1106m/z | N/A | 0.84 | 0.05 |
| 12.51_806.1761m/z | N/A | 1.39 | 0.05 |
| 8.35_650.7129m/z | N/A | 1.55 | 0.08 |
| 8.53_2282.1461n | N/A | 1.55 | 0.07 |
| 0.59_718.6199n | N/A | 0.87 | 0.09 |
| 12.59_896.6563m/z | N/A | 1.34 | 0.04 |
| 12.59_896.4892m/z | N/A | 1.28 | 0.09 |
| 0.41_191.9953n | N/A | 0.92 | 0.06 |
| 14.10_982.7376n | N/A | 0.85 | 0.07 |
| 12.51_782.9283n | N/A | 1.33 | 0.06 |
| 12.51_796.6893m/z | N/A | 1.33 | 0.06 |

^a^Cut-off criterion for the associated peaks/metabolites is p<0.1 without adjusting for covariates; the unidentified or unannotated peaks are listed with retention (RT) and exact mass (m/z or neutral mass). *Metabolites that predict PTB after adjusting for covariates (p<0.1). ^b^ Ontology levels: OL1, highly confident identification based on matching with In-house physical standard library (IPSL) via retention time (RT, with RT error≤|0.5|), exact mass (MS, with mass error<5ppm), and tandem mass similarity (MS/MS, with similarity ≥30); OL2a, confident identification based on matching with IPSL via MS and RT; OL2b, annotation for the isomer or derivatives of the compound listed but not the compound itself, based on matching with IPSL via MS and MS/MS; PDa, annotation based on matching with public database via MS

and experimental MS/MS (could be the listed compound, or the isomer or derivatives of the listed compound); PDb, annotation based on matching with public database via MS and predict MS/MS; PDc, annotation for the listed compound based on matching with public database via MS and isotopic similarity or adducts; PDd annotation for listed compound based on matching with public database via MS; N/A, peak was not identified or annotated. ^c^FC, fold change, the ratio of intensity between the PTB subjects vs control, based on the mean, indicates the direction and magnitude of change: FC>1.0 indicates increase compared to control and FC<1.0 indicates decrease compared to control. ^d^ *p*-value determined by logistic modeling.

**Table S6. UPLC-HR-MS determined metabolites/peaks associated with preterm birth (sPTB) (p<0.1)**

| **Metabolite/peak**  **(298 peaks, 135 with annotation or identification)** ^a^ | **Ontology level ^b^** | **FC^c^** | ***p*-value^d^** |
| --- | --- | --- | --- |
| Hydrocinnamic acid | OL1 | 0.73 | 0.08 |
| Serine | OL1 | 0.93 | 0.07 |
| Bisphenol S* | OL1 | 0.90 | 0.06 |
| Corticosterone* | OL1 | 1.40 | 0.05 |
| Cortisol | OL1 | 1.18 | 0.09 |
| Glutarate | OL1 | 0.90 | 0.03 |
| 3,4-dihydroxyphenylacetate | OL2a | 0.86 | 0.05 |
| 3-hydroxybutanoate | OL2a | 0.84 | 0.02 |
| 3-hydroxybutanoate | OL2a | 0.86 | 0.08 |
| Cortisol | OL2a | 1.55 | 0.03 |
| Salicylamide | OL2a | 0.77 | 0.08 |
| (R,S)-N-Acetyl-S-(2-hydroxy-3-buten-1-yl)-L-cysteine | OL2a | 0.84 | 0.08 |
| Glutarate* | OL2b | 0.92 | 0.07 |
| 2,6-dimethoxyphenol | OL2b | 0.63 | 0.06 |
| Pyridoxal | OL2b | 0.86 | 0.02 |
| 3-Hydroxy-3-methylglutaric acid | OL2b | 0.91 | 0.07 |
| γ,γ -Dimethylallyl pyrophosphate triammonium salt | OL2b | 0.82 | 0.10 |
| 2-phenylpropionate | OL2b | 0.85 | 0.07 |
| 2',4'-dihydroxyacetophenone | OL2b | 0.91 | 0.09 |
| Trans,trans-Muconic acid | PDa | 0.89 | 0.04 |
| Met-ala | PDa | 0.70 | 0.04 |
| 17-hydroxyprogesterone | PDa | 1.31 | 0.04 |
| Ser Pro Phe | PDa | 0.59 | 0.04 |
| Methionine | PDa | 0.88 | 0.04 |
| N,N-Dimethyl-L-valine | PDa | 1.45 | 0.07 |
| Asparaginyl-lysine | PDb | 0.55 | 0.04 |
| Biocytin | PDb | 0.73 | 0.07 |
| Cortolone-3-glucuronide | PDb | 1.30 | 0.03 |
| Pyrrolidine | PDb | 0.92 | 0.07 |
| [6]-Gingerdiol 3,5-diacetate | PDb | 1.30 | 0.07 |
| Succinyladenosine | PDb | 0.88 | 0.08 |
| 3-Hexenedioic acid* | PDc | 0.92 | 0.10 |
| 3-Sulfopyruvic acid* | PDc | 0.87 | 0.01 |
| Thr-Gly* | PDc | 0.88 | 0.05 |
| (1xi,3S)-1,2,3,4-Tetrahydro-1-methyl-beta-carboline-1,3-dicarboxylic acid | PDc | 0.49 | 0.06 |
| (3,4-Dihydroxyphenyl)ethanol | PDc | 0.89 | 0.03 |
| Anisoxide | PDc | 1.36 | 0.01 |
| 1-(9Z-Octadecenoyl)-sn-glycero-3-phospho-(1'-myo-inositol) | PDc | 1.64 | 0.03 |
| 1-Hexanol, 6-amino- | PDc | 0.85 | 0.03 |
| 2-aminoheptanoate | PDc | 0.93 | 0.07 |
| 2-methyl-1,3-thiazolidine-2-carboxamide | PDc | 0.90 | 0.05 |
| 3-Hexenedioic acid | PDc | 0.84 | <0.01 |
| 3-hydroxyoctanoyl carnitine | PDc | 1.21 | 0.08 |
| 4-hydroxy-3-nitrophenylacetate | PDc | 0.83 | 0.02 |
| 4-Hydroxycyclohexylcarboxylic acid | PDc | 0.87 | 0.01 |
| 5-[2H-Pyrrol-4-(3H)-ylidenemethyl]-2-furanmethanol | PDc | 0.80 |  |
| 5-phosphoribosylamine | PDc | 0.48 | 0.05 |
| 6-[1-(6,7-dimethoxy-2H-1,3-benzodioxol-5-yl)-3-oxopropoxy]-3,4,5-trihydroxyoxane-2-carboxylic acid | PDc | 0.72 | 0.08 |
| Acrylamide | PDc | 0.73 | 0.09 |
| Arecoline | PDc | 0.80 | 0.08 |
| Carbamazepine-O-quinone | PDc | 0.90 | 0.06 |
| Cysteinyl-proline | PDc | 0.87 | 0.07 |
| Dacarbazine | PDc | 0.85 | 0.03 |
| Di(propylene glycol) propyl ether | PDc | 0.76 | 0.01 |
| Ethyl 5-hexenoate | PDc | 0.79 | 0.07 |
| Gamma-Glutaminyl-4-hydroxybenzene | PDc | 0.79 | 0.08 |
| Glycylprolylhydroxyproline | PDc | 0.66 | 0.03 |
| Grepafloxacin | PDc | 1.23 | 0.06 |
| Homoarecoline | PDc | 0.82 | 0.07 |
| Isoeugenitin | PDc | 0.57 | 0.10 |
| Isopentyl pyrophosphate | PDc | 0.81 | 0.02 |
| Jasmonic acid | PDc | 0.45 | 0.10 |
| Levulinic acid | PDc | 0.92 | 0.05 |
| N,N-Dimethyl-L-valine | PDc | 0.76 | 0.02 |
| N-ethylmaleimide | PDc | 0.86 | 0.06 |
| Nitroacetic acid ethyl ester | PDc | 0.77 | 0.01 |
| Nitroguanidine | PDc | 0.78 | 0.04 |
| S-(2-Aminoethyl)isothiourea | PDc | 0.78 | 0.03 |
| Testosterone enanthate | PDc | 1.50 | 0.01 |
| Val-met | PDc | 2.03 | 0.04 |
| (R) 2,3-Dihydroxy-3-methylvalerate | PDc | 0.82 | 0.07 |
| Argininic acid | PDc | 0.84 | 0.08 |
| 2-O-p-Coumaroyltartronic acid | PDc | 0.79 | 0.09 |
| Aminofurantoin | PDc | 0.83 | 0.06 |
| 3,4-Dihydro-6-methoxy-3,7-dimethyl-1H-2-benzopyran-8-ol | PDc | 0.81 | 0.05 |
| Methyl sorbate | PDc | 0.81 | 0.10 |
| 3-Amino-2-piperidone | PDc | 0.79 | 0.07 |
| 4-hydroxy-1-(4-methoxyphenyl)pentan-3-one | PDc | 1.26 | 0.06 |
| Indole-3-carbinol | PDc | 0.88 | 0.08 |
| Ala-ala | PDc | 0.88 | 0.07 |
| Isopentenyl pyrophosphate | PDc | 0.72 | 0.05 |
| L-formylkynurenine | PDc | 0.76 | 0.04 |
| (2r,2's)-isobuteine | PDc | 3169.76 | 0.08 |
| 4-Amino-2-methylpyrimidine-5-carbonitrile | PDc | 0.81 | 0.05 |
| Picolinoylglycine | PDc | 0.85 | 0.09 |
| 2,3-Methyleneglutaric acid | PDc | 0.63 | 0.08 |
| 8-hydroxycarvotanacetone | PDc | 0.91 | 0.07 |
| 2-amino-4-({1-[(carboxymethyl)-C-hydroxycarbonimidoyl]-2-{[2-hydroxy-3-(4-hydroxyphenyl)-1-(5-methoxy-2,2-dimethyl-2H-chromen-6-yl)-3-oxopropyl]sulfanyl}ethyl}-C-hydroxycarbonimidoyl)butanoic acid* | PDd | 1.35 | 0.03 |
| 3,3'-Thiobispropanoic acid* | PDd | 0.84 | 0.01 |
| 5-(2-Methylpropyl)tetrahydro-2-oxo-3-furancarboxylic acid* | PDd | 0.92 | 0.06 |
| Chalcone* | PDd | 0.89 | 0.02 |
| Lyciumoside II* | PDd | 1.50 | 0.02 |
| Lys-pro-arg* | PDd | 1.26 | 0.06 |
| Phe-arg-arg* | PDd | 0.47 | 0.03 |
| Torososide B* | PDd | 1.30 | 0.04 |
| (±)- 2-butylthiazolidine | PDd | 0.88 | 0.04 |
| (±)- 2-propylthiazolidine | PDd | 0.83 | 0.02 |
| {3-[(2E)-3-phenylprop-2-enoyl]phenyl}oxidanesulfonic acid | PDd | 0.73 | 0.01 |
| 1-Propenyl 1-(1-propenylthio)propyl disulfide | PDd | 0.86 | 0.07 |
| 2,4,5,7alpha-Tetrahydro-1,4,4,7a-tetramethyl-1H-inden-2-ol | PDd | 0.87 | 0.02 |
| 2,6-Dimethyl-naphtalene | PDd | 0.70 | 0.01 |
| 27-norcholestanehexol | PDd | 1.24 | 0.02 |
| 2-Amino-a-carboline | PDd | 0.89 | 0.10 |
| 2'-hydroxyacetanilide | PDd | 0.86 | <0.01 |
| 3-[(1E)-1-{4-[2-(dimethylamino)ethoxy]phenyl}-1-(3-hydroxyphenyl)but-1-en-2-yl]phenol | PDd | 0.69 | 0.06 |
| 3-Methylbutyl glucosinolate | PDd | 0.67 | 0.07 |
| 4,7,8-trihydroxy-2H-chromen-2-one | PDd | 0.83 | 0.02 |
| 4-methoxycoumarin | PDd | 0.63 | 0.07 |
| 4-nitrobenzylamine | PDd | 0.87 | 0.09 |
| 4-Oxo-1-(3-pyridyl)-1-butanone | PDd | 0.84 | 0.08 |
| 8-[3,7-dihydroxy-2-(3-hydroxyphenyl)-3,4-dihydro-2H-1-benzopyran-4-yl]-6-[2-(3,4-dihydroxyphenyl)-3,7-dihydroxy-3,4-dihydro-2H-1-benzopyran-4-yl]-2-(3-hydroxyphenyl)-3,4-dihydro-2H-1-benzopyran-3,5,7-triol | PDd | 1.55 | 0.10 |
| Celiprolol | PDd | 1.18 | 0.02 |
| Cevadine | PDd | 1.87 | 0.05 |
| Chalcone | PDd | 0.83 | 0.02 |
| Dibenzylamine | PDd | 0.90 | 0.09 |
| Gln-met(o)-lys | PDd | 0.72 | 0.09 |
| Ile-leu | PDd | 1.60 | 0.02 |
| Indole-2-carboxylic acid | PDd | 0.85 | 0.02 |
| Leu-pro-lys | PDd | 1.19 | 0.02 |
| Phosphonic acid, 1,2-ethanediylbis-, tetraethyl ester | PDd | 1.98 | 0.01 |
| S-2,5-Dimethyl-3-furanyl 3-methylbutanethioate | PDd | 0.67 | 0.04 |
| Talinolol | PDd | 1.18 | 0.01 |
| Tetrahydro-2,5-furan-diacetic acid | PDd | 0.83 | 0.03 |
| (Z)-[3-(Methylsulfinyl)-1-propenyl] 2-propenyl disulfide | PDd | 0.38 | 0.09 |
| 2-Oxindole-3-acetate | PDd | 0.84 | 0.07 |
| 2-[(sulfooxy)methyl]butanoic acid | PDd | 0.85 | 0.08 |
| 2,5-Dihydro-4,5-dimethyl-2-(1-methylpropyl)thiazole | PDd | 1.71 | 0.10 |
| Pro ile | PDd | 0.79 | 0.05 |
| 2-[(sulfooxy)methyl]butanoic acid | PDd | 0.88 | 0.06 |
| Benzoic acid, 2-hydroxy-5-(methylthio)- | PDd | 0.86 | 0.08 |
| 11'-Carboxy-alpha-chromanol | PDd | 1.18 | 0.07 |
| 27-Nor-5b-cholestane-3a,7a,12a,24,25-pentol | PDd | 1.21 | 0.10 |
| Cis-3-Hexenyl phenylacetate | PDd | 0.93 | 0.10 |
| Persenone B | PDd | 1.21 | 0.09 |
| Mg(0:0/22:4(7z,10z,13z,16z)/0:0) | PDd | 1.14 | 0.09 |
| 0.77_143.9935m/z* | N/A | 0.76 | 0.06 |
| 0.69_132.0476n* | N/A | 0.88 | 0.10 |
| 7.21_420.1597m/z* | N/A | 5.57 | 0.09 |
| 15.62_684.9611m/z* | N/A | 1.21 | 0.04 |
| 2.74_205.0244m/z* | N/A | 0.86 | 0.03 |
| 2.87_139.9948m/z* | N/A | 0.80 | 0.01 |
| 3.34_991.4879n* | N/A | 0.52 | 0.06 |
| 4.44_117.0663n* | N/A | 0.91 | 0.05 |
| 7.51_1128.3677n* | N/A | 0.80 | 0.06 |
| 7.87_279.9912m/z* | N/A | 0.86 | 0.05 |
| 8.35_650.7129m/z* | N/A | 1.66 | 0.06 |
| 8.48_547.8199m/z* | N/A | 1.53 | 0.03 |
| 8.74_816.4079m/z* | N/A | 1.49 | 0.08 |
| 2.18_212.9639m/z* | N/A | 0.89 | 0.07 |
| 0.41_191.9953n | N/A | 0.90 | 0.05 |
| 0.51_102.9639m/z | N/A | 0.69 | 0.03 |
| 0.51_84.9549m/z | N/A | 0.86 | 0.08 |
| 0.53_441.0318m/z | N/A | 1.21 | 0.07 |
| 0.54_3097.3756n | N/A | 0.87 | 0.05 |
| 0.56_1016.7937m/z | N/A | 0.87 | 0.03 |
| 0.56_226.9463m/z | N/A | 0.86 | 0.09 |
| 0.56_560.8717m/z | N/A | 0.69 | 0.07 |
| 0.56_866.8245m/z | N/A | 0.81 | 0.08 |
| 0.56_90.9820m/z | N/A | 0.91 | 0.10 |
| 0.56_939.7901n | N/A | 0.84 | 0.04 |
| 0.59_615.6859n | N/A | 0.86 | 0.04 |
| 0.59_618.6671n | N/A | 0.90 | 0.08 |
| 0.59_718.6199n | N/A | 0.84 | 0.06 |
| 0.59_731.6171n | N/A | 0.85 | 0.08 |
| 0.59_821.5590m/z | N/A | 0.89 | 0.10 |
| 0.61_131.8962n | N/A | 0.79 | 0.06 |
| 0.61_403.8173m/z | N/A | 0.85 | 0.05 |
| 0.62_190.1379m/z | N/A | 1.42 | 0.05 |
| 0.64_336.9406m/z | N/A | 0.90 | 0.07 |
| 0.69_112.0281m/z | N/A | 0.77 | 0.07 |
| 0.78_210.8558m/z | N/A | 0.91 | 0.09 |
| 0.83_121.0077m/z | N/A | 0.88 | 0.05 |
| 0.85_226.9339m/z | N/A | 0.79 | 0.03 |
| 0.88_150.9002m/z | N/A | 0.83 | 0.03 |
| 0.88_208.8587m/z | N/A | 0.90 | 0.06 |
| 0.90_98.9979n | N/A | 0.87 | 0.09 |
| 1.16_459.0249m/z | N/A | 0.87 | 0.05 |
| 1.25_102.9640m/z | N/A | 0.85 | 0.02 |
| 1.48_103.0584m/z | N/A | 0.86 | 0.05 |
| 10.36_1827.0030n | N/A | 1.59 | 0.08 |
| 10.39_976.0815m/z | N/A | 0.75 | 0.06 |
| 10.49_471.3162m/z | N/A | 1.16 | 0.03 |
| 10.79_367.1872n | N/A | 1.13 | 0.07 |
| 11.36_479.2268n | N/A | 0.60 | 0.06 |
| 11.53_369.2028n | N/A | 1.63 | 0.09 |
| 11.74_455.3214m/z | N/A | 1.29 | <0.01 |
| 12.20_999.8890m/z | N/A | 1.63 | 0.08 |
| 12.28_222.0947m/z | N/A | 1.46 | 0.05 |
| 12.51_782.9283n | N/A | 1.34 | 0.07 |
| 12.51_796.6893m/z | N/A | 1.33 | 0.07 |
| 12.51_806.1761m/z | N/A | 1.42 | 0.04 |
| 12.53_809.9179m/z | N/A | 1.47 | 0.05 |
| 13.03_513.3635m/z | N/A | 1.24 | 0.03 |
| 13.16_805.6613m/z | N/A | 1.46 | 0.07 |
| 13.58_543.3973n | N/A | 1.15 | 0.02 |
| 13.61_571.4049m/z | N/A | 1.16 | 0.02 |
| 14.07_533.2666n | N/A | 1.32 | 0.07 |
| 14.10_1015.5208m/z | N/A | 0.73 | 0.01 |
| 14.10_1016.1106m/z | N/A | 0.82 | 0.04 |
| 14.10_982.7376n | N/A | 0.85 | 0.09 |
| 14.12_1015.9340m/z | N/A | 0.80 | 0.03 |
| 14.20_575.3697m/z | N/A | 1.22 | 0.04 |
| 14.38_514.2751n | N/A | 0.65 | 0.05 |
| 15.13_274.6633m/z | N/A | 1.22 | 0.07 |
| 15.52_1133.8245n | N/A | 1.13 | 0.05 |
| 15.64_124.2082m/z | N/A | 1.17 | 0.03 |
| 15.66_522.7946m/z | N/A | 1.12 | 0.02 |
| 15.66_770.4609n | N/A | 1.15 | <0.01 |
| 15.66_770.9636m/z | N/A | 1.11 | 0.02 |
| 15.68_454.2916n | N/A | 1.12 | 0.08 |
| 15.77_468.3763m/z | N/A | 1.35 | 0.05 |
| 15.86_669.9560m/z | N/A | 1.17 | 0.03 |
| 15.88_404.3160n | N/A | 0.83 | 0.01 |
| 15.97_291.6842m/z | N/A | 1.20 | 0.08 |
| 2.25_115.0584m/z | N/A | 0.86 | 0.04 |
| 2.30_308.8998m/z | N/A | 0.92 | 0.09 |
| 2.57_152.0646m/z | N/A | 0.81 | <0.01 |
| 2.87_262.9896m/z | N/A | 0.87 | 0.09 |
| 3.22_177.9928m/z | N/A | 0.91 | 0.07 |
| 3.34_134.9673m/z | N/A | 0.79 | <0.01 |
| 3.56_120.9891m/z | N/A | 0.86 | 0.01 |
| 3.56_149.9792m/z | N/A | 0.90 | 0.07 |
| 4.32_132.0440n | N/A | 0.71 | 0.02 |
| 4.44_209.1030n | N/A | 0.87 | 0.10 |
| 5.28_296.6236m/z | N/A | 1.67 | 0.09 |
| 5.28_556.2997m/z | N/A | 1.75 | 0.09 |
| 5.71_203.0591m/z | N/A | 0.52 | 0.04 |
| 6.17_417.2344m/z | N/A | 2.00 | 0.06 |
| 6.19_219.0540m/z | N/A | 0.90 | 0.05 |
| 6.30_477.7721m/z | N/A | 1.91 | 0.04 |
| 6.40_431.9056m/z | N/A | 0.67 | 0.10 |
| 7.21_243.0208n | N/A | 0.88 | 0.08 |
| 7.21_535.3096m/z | N/A | 0.65 | 0.07 |
| 7.29_507.2374m/z | N/A | 0.41 | 0.03 |
| 7.51_591.6469m/z | N/A | 0.75 | 0.08 |
| 7.64_2049.0704n | N/A | 1.40 | 0.06 |
| 7.79_216.0311n | N/A | 0.68 | 0.06 |
| 8.17_572.5371m/z | N/A | 0.41 | 0.05 |
| 8.17_572.7879m/z | N/A | 0.45 | 0.06 |
| 8.20_833.3900m/z | N/A | 0.72 | 0.07 |
| 8.20_837.6319m/z | N/A | 0.63 | 0.06 |
| 8.38_571.2848m/z | N/A | 0.51 | 0.09 |
| 8.48_548.0706m/z | N/A | 1.48 | 0.04 |
| 8.53_2282.1461n | N/A | 1.55 | 0.10 |
| 8.56_861.0300m/z | N/A | 0.68 | 0.07 |
| 8.63_547.2084m/z | N/A | 0.74 | 0.03 |
| 8.63_600.1835m/z | N/A | 1.36 | 0.08 |
| 8.66_761.8944m/z | N/A | 0.40 | 0.04 |
| 8.66_762.1452m/z | N/A | 0.35 | 0.03 |
| 8.71_1016.7787m/z | N/A | 1.39 | 0.08 |
| 8.74_795.2020m/z | N/A | 0.48 | 0.04 |
| 8.91_763.3911m/z | N/A | 0.54 | 0.07 |
| 8.91_763.6418m/z | N/A | 0.47 | 0.04 |
| 8.91_794.9990m/z | N/A | 0.48 | 0.02 |
| 8.91_795.3987m/z | N/A | 0.66 | 0.04 |
| 9.09_1022.5013m/z | N/A | 1.89 | 0.04 |
| 9.14_791.5979m/z | N/A | 0.72 | 0.08 |
| 9.14_791.9991m/z | N/A | 0.73 | 0.08 |
| 9.57_839.4082m/z | N/A | 1.36 | 0.07 |
| 0.51_102.9687m/z | N/A | 0.82 | 0.06 |
| 3.64_187.0278m/z | N/A | 0.89 | 0.10 |
| 3.47_226.9862m/z | N/A | 0.47 | 0.05 |
| 8.50_582.0176m/z | N/A | 0.41 | 0.10 |
| 15.59_425.3623m/z | N/A | 1.38 | 0.07 |
| 3.92_142.0557m/z | N/A | 0.85 | 0.05 |
| 7.97_372.1951m/z | N/A | 0.77 | 0.03 |
| 5.56_259.0489m/z | N/A | 0.89 | 0.07 |
| 3.04_454.1821m/z | N/A | 0.88 | 0.09 |
| 0.73_281.1218m/z | N/A | 1.35 | 0.07 |
| 3.89_186.0584m/z | N/A | 0.90 | 0.03 |
| 8.79_401.6578m/z | N/A | 0.58 | 0.08 |
| 4.62_563.4950m/z | N/A | 0.70 | 0.04 |
| 2.01_132.0788m/z | N/A | 0.86 | 0.10 |
| 13.66_616.2518n | N/A | 1.54 | 0.09 |
| 8.91_606.5187m/z | N/A | 0.67 | 0.09 |
| 14.74_558.3148m/z | N/A | 1.17 | 0.06 |
| 3.49_143.0340m/z | N/A | 0.82 | 0.01 |
| 13.68_882.0493m/z | N/A | 1.28 | 0.06 |
| 7.21_187.1264m/z | N/A | 0.92 | 0.10 |
| 11.20_754.9719m/z | N/A | 1.46 | 0.08 |
| 5.97_423.7634m/z | N/A | 0.54 | 0.05 |
| 8.91_794.7967m/z | N/A | 0.61 | 0.04 |
| 15.44_431.8123m/z | N/A | 1.18 | 0.09 |
| 15.57_579.3383m/z | N/A | 1.14 | 0.08 |
| 7.94_694.3458m/z | N/A | 1.45 | 0.09 |
| 13.66_976.1573m/z | N/A | 1.17 | 0.09 |
| 0.61_342.7793m/z | N/A | 0.91 | 0.08 |
| 8.20_827.6441m/z | N/A | 0.79 | 0.08 |
| 6.78_575.9934m/z | N/A | 0.62 | 0.09 |
| 11.76_433.1578n | N/A | 1.59 | 0.07 |
| 4.20_218.0468n | N/A | 0.88 | 0.10 |
| 14.10_966.4965m/z | N/A | 0.83 | 0.10 |
| 8.20_814.1339n | N/A | 0.78 | 0.07 |
| 15.84_820.9780m/z | N/A | 1.17 | 0.08 |
| 0.59_726.6068n | N/A | 0.87 | 0.10 |
| 9.14_792.1997m/z | N/A | 0.74 | 0.09 |
| 11.23_1863.4306n | N/A | 1.34 | 0.09 |
| 3.19_150.0893n | N/A | 0.85 | 0.06 |

^a^Cut-off criterion for the associated peaks/metabolites is p<0.1 without adjusting for covariates; the unidentified or unannotated peaks are listed with retention (RT) and exact mass (m/z or neutral mass). *Metabolites that predict SPTB after adjusting for covariates (p<0.1). ^b^ Ontology levels: OL1, highly confident identification based on matching with In-house physical standard library (IPSL) via retention time (RT, with RT error≤|0.5|), exact mass (MS, with mass error<5ppm), and tandem mass similarity (MS/MS, with similarity ≥30); OL2a, confident identification based on matching with IPSL via MS and RT; OL2b, annotation for the isomer or derivatives of the compound listed but not the compound itself, based on matching with IPSL via MS and MS/MS; PDa, annotation based on matching with public database via MS and experimental MS/MS (could be the listed compound, or the isomer or derivatives of the listed compound); PDb, annotation based on matching with public database via MS and predict MS/MS; PDc, annotation for the listed compound based on matching with public database via MS and isotopic similarity or adducts; PDd annotation for listed compound based on matching with public database via MS; N/A, peak was not identified or annotated. ^c^FC, fold change, the ratio of intensity between the SPTB subjects vs control, based on the mean, indicates the direction and magnitude of change: FC>1.0 indicates increase compared to control and FC<1.0 indicates decrease compared to control. ^d^ *p*-value determined by logistic modeling.

**Table S7. Nuclear magnetic resonance (NMR)-determined bins, library matched to metabolites associated with hypertensive disorders in pregnancy (HDP) (p<0.1), U1, U2 = Unknown**

| **Bin** | **NMR Bin**  **(in ppm)** | p-value | **Fold Change** | **Metabolite Annotations** |
| --- | --- | --- | --- | --- |
|  |  |  | HDP/control |  |
| b_1 | [0.48 .. 0.50] | 0.06 | 0.680 | Albumin |
| b_3 | [0.52 .. 0.55] | 0.046 | 0.707 | Albumin |
| b_4 | [0.55 .. 0.58] | 0.025 | 0.736 | Albumin |
| b_5 | [0.58 .. 0.60] | 0.022 | 0.737 | Albumin |
| b_6 | [0.60 .. 0.62] | 0.033 | 0.746 | Albumin |
| b_7 | [0.62 .. 0.64] | 0.036 | 0.788 | Albumin |
| b_8 | [0.64 .. 0.69] | 0.021 | 0.809 | Cholesterol (in HDL and VLDL) |
| b_10 | [0.71 .. 0.73] | 0.08 | 0.720 | Lipids |
| b_11 | [0.73 .. 0.75] | 0.081 | 0.538 | Lipids |
| b_12 | [0.75 .. 0.77] | 0.08 | 0.424 | Lipids |
| b_16 | [0.87 .. 0.89] | 0.038 | 1.123 | Lipids |
| b_20* | [1.06 .. 1.08]* | 0.01 | 0.877 | Overlapped |
| b_21* | [1.08 .. 1.10]* | 0.006 | 0.800 | Overlapped |
| b_24* | [1.14 .. 1.16]* | 0.008 | 0.730 | U \| Lipids |
| b_25 | [1.16 .. 1.18] | 0.018 | 0.733 | Lipids |
| b_29 | [1.29 .. 1.35] | 0.018 | 1.117 | Lactate \| Lipids (mainly VLDL) \| Threonine |
| b_45 | [1.91 .. 1.93] | 0.028 | 0.804 | Arginine |
| b_51 | [2.13 .. 2.15] | 0.051 | 0.919 | Fatty acids |
| b_61* | [2.54 .. 2.56]* | 0.062 | 0.777 | Overlapped |
| b_64 | [2.61 .. 2.63] | 0.079 | 0.902 | Methionine |
| b_65 | [2.63 .. 2.65] | 0.062 | 0.921 | Methionine \| Aspartate |
| b_66* | [2.65 .. 2.70]* | 0.053 | 0.952 | Aspartate \| Citrate |
| b_70* | [2.84 .. 2.86]* | 0.05 | 0.907 | Asparagine |
| b_71* | [2.86 .. 2.91]* | 0.004 | 0.891 | Asparagine \| N,N-Dimethylglycine \| Trimethylamine |
| b_72* | [2.91 .. 2.93]* | 0.004 | 0.846 | Asparagine |
| b_73* | [2.93 .. 2.95]* | 0.004 | 0.872 | Asparagine \| Albumin (Lysyl) |
| b_74* | [2.95 .. 2.97]* | 0.003 | 0.897 | Asparagine \| Albumin (Lysyl) |
| b_76 | [3.03 .. 3.06] | 0.09 | 0.947 | Creatinine \| Tyrosine |
| b_77* | [3.06 .. 3.12]* | 0.006 | 0.881 | t-Methylhistidine |
| b_79* | [3.15 .. 3.17]* | 0.08 | 0.618 | t-Methylhistidine |
| b_82 | [3.28 .. 3.30] | 0.068 | 0.938 | Proline |
| b_84 | [3.33 .. 3.37] | 0.077 | 0.866 | Methanol \| Proline |
| b_90* | [3.58 .. 3.61]* | 0.042 | 0.935 | Threonine \| Valine |
| b_91* | [3.61 .. 3.63]* | 0.088 | 0.846 | myo-Inositol |
| b_102* | [4.14 .. 4.17]* | 0.044 | 0.881 | 3-Hydroxybutyrate \| O-Phosphocholine |
| b_103* | [4.17 .. 4.19]* | 0.073 | 0.883 | O-Phosphocholine |
| b_104* | [4.19 .. 4.21]* | 0.089 | 0.889 | Sugars \| UDP-Sugars |
| b_121* | [5.27 .. 5.33]* | 0.077 | 1.072 | Unsaturated lipids |
| b_122 | [5.33 .. 5.39] | 0.082 | 1.100 | Unsaturated lipids |
| b_132 | [5.73 .. 5.76] | 0.068 | 0.858 | Urea |
| b_133 | [5.76 .. 5.82] | 0.073 | 0.859 | Urea |
| b_155 | [6.62 .. 6.66] | 0.079 | 0.784 | Below limit of identification |
| b_158* | [6.70 .. 6.75]* | 0.007 | 0.878 | U |
| b_159 | [6.75 .. 6.77] | 0.02 | 0.880 | Overlapped |
| b_160 | [6.77 .. 6.79] | 0.023 | 0.905 | Overlapped |
| b_161* | [6.79 .. 6.81]* | 0.026 | 0.890 | Overlapped |
| b_162* | [6.81 .. 6.85]* | 0.008 | 0.876 | Overlapped |
| b_166* | [6.95 .. 7.00]* | 0.032 | 0.931 | Overlapped |
| b_167 | [7.00 .. 7.03] | 0.038 | 0.925 | t-Methylhistidine |
| b_169* | [7.09 .. 7.11]* | 0.028 | 0.821 | Overlapped |
| b_170* | [7.11 .. 7.16]* | 0.025 | 0.865 | Overlapped |
| b_172 | [7.18 .. 7.22] | 0.065 | 0.936 | Tryptophan \| Tyrosine |
| b_173* | [7.22 .. 7.24] * | 0.023 | 0.900 | Overlapped |
| b_174* | [7.24 .. 7.28]* | 0.039 | 0.930 | Tryptophan |
| b_175 | [7.28 .. 7.30] | 0.046 | 0.942 | Phenylalanine |
| b_179 | [7.46 .. 7.51] | 0.07 | 0.862 | Below limit of identification |
| b_180* | [7.51 .. 7.54]* | 0.014 | 0.808 | Below limit of identification |
| b_181 | [7.54 .. 7.56] | 0.041 | 0.882 | Tryptophan |
| b_183 | [7.58 .. 7.64] | 0.056 | 0.908 | Overlapped |
| b_186* | [7.69 .. 7.71]* | 0.076 | 0.916 | Overlapped |

*Remained significant after adjusting for covariates (p<0.1)

**Table S8. Nuclear magnetic resonance (NMR)-determined bins, library-matched to metabolites associated with gestational hypertension (GH) (p<0.1), U1, U2 = Unknown**

| **Bin** | **NMR Bin**  **(in ppm)** | p-value | **Fold Change** | **Metabolite Annotations** |
| --- | --- | --- | --- | --- |
|  |  |  | GH/control |  |
| b_3 | [0.52 .. 0.55] | 0.096 | 0.708 | Albumin |
| b_4 | [0.55 .. 0.58] | 0.089 | 0.747 | Albumin |
| b_5 | [0.58 .. 0.60] | 0.054 | 0.739 | Albumin |
| b_6 | [0.60 .. 0.62] | 0.089 | 0.753 | Albumin |
| b_7 | [0.62 .. 0.64] | 0.098 | 0.794 | Albumin |
| b_8 | [0.64 .. 0.69] | 0.063 | 0.814 | Cholesterol (in HDL and VLDL) |
| b_12 | [0.75 .. 0.77] | 0.099 | 0.421 | Lipids |
| b_17 | [0.89 .. 0.95] | 0.091 | 1.046 | Cholesterol \| Lipids \| Isoleucine \| Leucine \| Valine |
| b_20 | [1.06 .. 1.08] | 0.067 | 0.889 | Overlapped |
| b_21* | [1.08 .. 1.10]* | 0.041 | 0.805 | Overlapped |
| b_24 | [1.14 .. 1.16] | 0.067 | 0.738 | U \| Lipids |
| b_25 | [1.16 .. 1.18] | 0.074 | 0.736 | Lipids |
| b_29 | [1.29 .. 1.35] | 0.067 | 1.095 | Lactate \| Lipids (mainly VLDL) \| Threonine |
| b_49 | [2.03 .. 2.08] | 0.081 | 1.047 | Glutamate \| Proline |
| b_71 | [2.86 .. 2.91] | 0.058 | 0.921 | Asparagine \| N,N-Dimethylglycine \| Trimethylamine |
| b_72 | [2.91 .. 2.93] | 0.042 | 0.870 | Asparagine |
| b_73 | [2.93 .. 2.95] | 0.039 | 0.893 | Asparagine \| Albumin (Lysyl) |
| b_74 | [2.95 .. 2.97] | 0.037 | 0.919 | Asparagine \| Albumin (Lysyl) |
| b_77 | [3.06 .. 3.12] | 0.07 | 0.908 | t-Methylhistidine |
| b_102* | [4.14 .. 4.17] | 0.059 | 0.866 | 3-Hydroxybutyrate \| O-Phosphocholine |
| b_122 | [5.33 .. 5.39] | 0.094 | 1.111 | Unsaturated lipids |
| b_132 | [5.73 .. 5.76] | 0.093 | 0.846 | Urea |
| b_158 | [6.70 .. 6.75] | 0.054 | 0.902 | U |
| b_159 | [6.75 .. 6.77] | 0.079 | 0.894 | Overlapped |
| b_160 | [6.77 .. 6.79] | 0.063 | 0.909 | Overlapped |
| b_162 | [6.81 .. 6.85] | 0.069 | 0.899 | Overlapped |
| b_170 | [7.11 .. 7.16] | 0.06 | 0.866 | Overlapped |
| b_173 | [7.22 .. 7.24] | 0.089 | 0.911 | Overlapped |
| b_180 | [7.51 .. 7.54] | 0.054 | 0.819 | Below limit of identification |

*Remained significant after adjusting for covariates (p<0.1)

**Table S9. Nuclear magnetic resonance (NMR)-determined bins, library matched to metabolites associated with preeclampsia (PE) (p<0.1), U1, U2 = Unknown**

| **Bin** | **NMR Bin**  **(in ppm)** | p-value | Fold Change | **Metabolite Annotations** |
| --- | --- | --- | --- | --- |
|  |  |  | PE/control |  |
| b_4* | [0.55 .. 0.58]* | 0.076 | 0.715 | Albumin |
| b_8* | [0.64 .. 0.69]* | 0.095 | 0.801 | Cholesterol (in HDL and VLDL) |
| b_16* | [0.87 .. 0.89]* | 0.033 | 1.196 | Lipids |
| b_20* | [1.06 .. 1.08] * | 0.022 | 0.855 | Overlapped |
| b_21* | [1.08 .. 1.10] * | 0.015 | 0.791 | Overlapped |
| b_24* | [1.14 .. 1.16]* | 0.013 | 0.716 | U \| Lipids |
| b_25* | [1.16 .. 1.18]* | 0.053 | 0.726 | Lipids |
| b_28* | [1.23 .. 1.29]* | 0.065 | 1.109 | Fucose \| Isoleucine \| Lipids (mainly LDL) |
| b_29* | [1.29 .. 1.35]* | 0.04 | 1.156 | Lactate \| Lipids (mainly VLDL) \| Threonine |
| b_37* | [1.62 .. 1.65]* | 0.035 | 0.926 | Arginine |
| b_38* | [1.65 .. 1.67]* | 0.02 | 0.930 | Arginine \| Leucine |
| b_43* | [1.83 .. 1.86]* | 0.087 | 0.872 | Citruline |
| b_44* | [1.86 .. 1.91]* | 0.076 | 0.878 | Acetate \| Arginine \| Citruline \| Lysine |
| b_45* | [1.91 .. 1.93]* | 0.024 | 0.778 | Arginine |
| b_46* | [1.93 .. 1.95]* | 0.054 | 0.806 | Proline |
| b_47 | [1.95 .. 1.97] | 0.08 | 0.867 | Lipids \| Proline |
| b_52* | [2.15 .. 2.17]* | 0.067 | 0.821 | Fatty acids |
| b_59* | [2.46 .. 2.48]* | 0.088 | 0.903 | Glutamine |
| b_64* | [2.61 .. 2.63]* | 0.039 | 0.844 | Methionine |
| b_65* | [2.63 .. 2.65]* | 0.083 | 0.891 | Methionine \| Aspartate |
| b_71* | [2.86 .. 2.91]* | 0.005 | 0.835 | Asparagine \| N,N-Dimethylglycine \| Trimethylamine |
| b_72* | [2.91 .. 2.93]* | 0.009 | 0.801 | Asparagine |
| b_73* | [2.93 .. 2.95]* | 0.011 | 0.835 | Asparagine \| Albumin (Lysyl) |
| b_74* | [2.95 .. 2.97]* | 0.007 | 0.856 | Asparagine \| Albumin (Lysyl) |
| b_75* | [2.97 .. 3.03]* | 0.059 | 0.907 | 2-Oxoglutarate \| Albumin (Lysyl) \| Lysine \| Creatine |
| b_76* | [3.03 .. 3.06]* | 0.061 | 0.907 | Creatinine \| Tyrosine |
| b_77* | [3.06 .. 3.12]* | 0.007 | 0.832 | t-Methylhistidine |
| b_79* | [3.15 .. 3.17]* | 0.088 | 0.600 | t-Methylhistidine |
| b_82* | [3.28 .. 3.30]* | 0.006 | 0.857 | Proline |
| b_83* | [3.30 .. 3.33]* | 0.084 | 0.648 | Proline |
| b_84* | [3.33 .. 3.37]* | 0.0007 | 0.735 | Methanol \| Proline |
| b_90* | [3.58 .. 3.61]* | 0.013 | 0.878 | Threonine \| Valine |
| b_91* | [3.61 .. 3.63]* | 0.084 | 0.822 | myo-Inositol |
| b_92* | [3.63 .. 3.68]* | 0.039 | 0.892 | Fucose \| Glycerol \| myo-Inositol |
| b_98* | [3.95 .. 4.01]* | 0.041 | 0.896 | Histidine \| Phenylalanine |
| b_157* | [6.68 .. 6.70]* | 0.042 | 0.836 | U |
| b_158* | [6.70 .. 6.75]* | 0.022 | 0.835 | U |
| b_159* | [6.75 .. 6.77]* | 0.069 | 0.853 | Overlapped |
| b_161* | [6.79 .. 6.81]* | 0.027 | 0.844 | Overlapped |
| b_162* | [6.81 .. 6.85]* | 0.014 | 0.833 | Overlapped |
| b_163* | [6.85 .. 6.88]* | 0.06 | 0.891 | Tyrosine |
| b_166* | [6.95 .. 7.00]* | 0.022 | 0.886 | Overlapped |
| b_167* | [7.00 .. 7.03]* | 0.031 | 0.877 | t-Methylhistidine |
| b_168* | [7.03 .. 7.09]* | 0.094 | 0.895 | Histidine \| t-Methylhistidine |
| b_169* | [7.09 .. 7.11]* | 0.056 | 0.785 | Overlapped |
| b_173* | [7.22 .. 7.24]* | 0.066 | 0.880 | Overlapped |
| b_175* | [7.28 .. 7.30]* | 0.042 | 0.908 | Phenylalanine |
| b_180* | [7.51 .. 7.54]* | 0.062 | 0.788 | Below limit of identification |
| b_183* | [7.58 .. 7.64]* | 0.044 | 0.846 | Overlapped |
| b_186* | [7.69 .. 7.71]* | 0.079 | 0.872 | Overlapped |

*Remained significant after adjusting for covariates (p<0.1)

**Table S10. Nuclear magnetic resonance (NMR)-determined bins, library matched to metabolites associated with preterm birth (PTB) (p<0.1)**

| **Bin** | **NMR Bin**  **(in ppm)** | **p-value** | **Fold Change** | **Metabolite Annotations** |
| --- | --- | --- | --- | --- |
|  |  |  | **(PTB/control)** |  |
| b_18 | [0.95 .. 1.00] | 0.044 | 0.942 | Isoleucine \| Leucine \| Valine |
| b_19* | [1.00 .. 1.06]* | 0.009 | 0.908 | Isoleucine \| Valine \| Isobutyrate |
| b_55* | [2.25 .. 2.31]* | 0.062 | 0.932 | Acetoacetate \| Valine |
| b_59 | [2.46 .. 2.48] | 0.046 | 0.924 | Glutamine |
| b_98* | [3.95 .. 4.01]* | 0.016 | 0.926 | Histidine \| Phenylalanine |
| b_106* | [4.23 .. 4.25]* | 0.026 | 0.941 | Threonine |
| b_108 | [4.31 .. 4.36] | 0.077 | 0.935 | Glyceryl of lipids |
| b_109* | [4.36 .. 4.42]* | 0.07 | 0.891 | UDP-Sugars |
| b_133 | [5.76 .. 5.82] | 0.063 | 0.855 | Urea |
| b_168 | [7.03 .. 7.09] | 0.023 | 0.913 | Histidine \| t-Methylhistidine |
| b_172 | [7.18 .. 7.22] | 0.051 | 0.935 | Tryptophan \| Tyrosine |
| b_189 | [7.75 .. 7.80] | 0.036 | 0.906 | U |
| b_190 | [7.80 .. 7.86] | 0.033 | 0.869 | U |

*Remained significant after adjusting for covariates (p<0.1)

**Table S11. Nuclear magnetic resonance (NMR)-determined bins, library-matched to metabolites associated with spontaneous preterm birth (SPTB) (univariate logistic regression, p<0.1), U = Unknown**

| **Bin** | **NMR Bin**  **(in ppm)** | p-value | **Fold Change** | **Metabolite Annotations** |
| --- | --- | --- | --- | --- |
|  |  |  |  |  |
| b_19* | [1.00 .. 1.06] * | 0.060 | 0.931 | Isoleucine \| Valine \| Isobutyrate |
| b_55* | [2.25 .. 2.31] * | 0.070 | 0.929 | Acetoacetate \| Valine |
| b_90 | [3.58 .. 3.61] | 0.100 | 0.944 | Threonine \| Valine |
| b_98* | [3.95 .. 4.01] * | 0.042 | 0.933 | Histidine \| Phenylalanine |
| b_106* | [4.23 .. 4.25] * | 0.098 | 0.954 | Threonine |
| b_132* | [5.73 .. 5.76] * | 0.056 | 0.840 | Urea |
| b_133* | [5.76 .. 5.82] * | 0.039 | 0.825 | Urea |
| b_168* | [7.03 .. 7.09] * | 0.083 | 0.928 | Histidine \| t-Methylhistidine |
| b_190* | [7.80 .. 7.86]* | 0.076 | 0.881 | U |

*Remained significant after adjusting for Gravidity (p<0.1)

**References**

1. Want EJ, Wilson ID, Gika H, et al. Global metabolic profiling procedures for urine using UPLC-MS. *Nature protocols* 2010; **5**: 1005-18.

2. Dunn WB, Broadhurst D, Begley P, et al. Procedures for large-scale metabolic profiling of serum and plasma using gas chromatography and liquid chromatography coupled to mass spectrometry. *Nature protocols* 2011; **6**: 1060-83.

3. Sandlers Y, Mercier K, Pathmasiri W, et al. Metabolomics Reveals New Mechanisms for Pathogenesis in Barth Syndrome and Introduces Novel Roles for Cardiolipin in Cellular Function. *PLoS One* 2016; **11**: e0151802.

4. Laine JE, Bailey KA, Olshan AF, et al. Neonatal Metabolomic Profiles Related to Prenatal Arsenic Exposure. *Environmental science & technology* 2017; **51**: 625-33.

5. Beckonert O, Keun HC, Ebbels TM, et al. Metabolic profiling, metabolomic and metabonomic procedures for NMR spectroscopy of urine, plasma, serum and tissue extracts. *Nature protocols* 2007; **2**: 2692-703.

6. Kuhn M. Building predictive models in R using the caret package. *Journal of Statistical Software* 2008; **28**.
